# Neonatal Hypoxia Ischaemia: Mechanisms, Models, and Therapeutic Challenges

**DOI:** 10.3389/fncel.2017.00078

**Published:** 2017-05-08

**Authors:** Lancelot J. Millar, Lei Shi, Anna Hoerder-Suabedissen, Zoltán Molnár

**Affiliations:** ^1^Molnár Group, Department of Physiology, Anatomy and Genetics, University of OxfordOxford, UK; ^2^JNU-HKUST Joint Laboratory for Neuroscience and Innovative Drug Research, College of Pharmacy, Jinan UniversityGuangzhou, China

**Keywords:** neonatal, hypoxia-ischemia, encephalopathy, subplate, neurodevelopment, neuroserpin, neuroprotection

## Abstract

Neonatal hypoxia-ischaemia (HI) is the most common cause of death and disability in human neonates, and is often associated with persistent motor, sensory, and cognitive impairment. Improved intensive care technology has increased survival without preventing neurological disorder, increasing morbidity throughout the adult population. Early preventative or neuroprotective interventions have the potential to rescue brain development in neonates, yet only one therapeutic intervention is currently licensed for use in developed countries. Recent investigations of the transient cortical layer known as subplate, especially regarding subplate’s secretory role, opens up a novel set of potential molecular modulators of neonatal HI injury. This review examines the biological mechanisms of human neonatal HI, discusses evidence for the relevance of subplate-secreted molecules to this condition, and evaluates available animal models. Neuroserpin, a neuronally released neuroprotective factor, is discussed as a case study for developing new potential pharmacological interventions for use post-ischaemic injury.

## Introduction: Global Clinical Impact of Neonatal Hypoxia Ischaemia

The clinical definition of neonatal HI injury is “asphyxia of the umbilical blood supply to the human fetus occurring at 36 gestational weeks or later” (Perlman, 1997, 2006; Volpe, 2001, 2012; Shah P.S. et al., 2006). Neonatal HI is synonymous with hypoxic-ischaemic encephalopathy (HIE) occurring in the term infant, where term is defined as 36 gestational weeks or later. This review addresses neonatal HI or HIE, any results concerning perinatal hypoxic-ischaemia injury will be clearly indicated in the text. This disorder encompasses a large range of physiological origins and clinical outcomes (Volpe, 1995). The diagnostic criteria for neonatal HI are based on a set of markers demonstrated to correlate with clinical outcome (Finer et al., 1981; Perlman, 1997; Richer et al., 2001). These include: 5-min Apgar score of less than 5 (Levene et al., 1986; Ruth and Raivio, 1988; Laptook et al., 2009); need for delivery room intubation or CPR (Richardson B.S. et al., 1996; Salhab et al., 2004a; Shalak and Perlman, 2004; Kattwinkel et al., 2010); umbilical cord arterial pH less than 7.00 (Ruth and Raivio, 1988; Perlman and Risser, 1993; Robertson N.J. et al., 2002; Salhab et al., 2004b); and abnormal neurological signs, such as hypotonic muscles or lack of sucking reflex (Levene et al., 1986; Robertson et al., 1989; Richer et al., 2001). Electroencephalography (EEG) has also proved helpful as a predictor of clinical outcome (reviewed in Walsh et al., 2011; van Laerhoven et al., 2013). Amplitude-integrated EEG (aEEG) in particular, a filtered time-compressed continuous one- or two-channel read-out, has been demonstrated a reliable predictor in meta-analyses up to 5 years after birth (al Naqeeb et al., 1999; Sinclair et al., 1999; Toet et al., 1999; Biagioni et al., 2001; Shah D.K. et al., 2006; Nash et al., 2011; Murray et al., 2016; Weeke et al., 2016), however, some report that aEEG remains less reliable than MRI, especially following hypothermia treatment (Doyle et al., 2010; Weeke et al., 2016). The Thompson score, an EEG measure of predictive neurodevelopment, is likely to remain useful to clinicians (Thompson et al., 1997; Horn et al., 2016). This is by no means an exhaustive list of risk factors and signs of clinical concern that can occur during the early postnatal period (Shalak and Perlman, 2004; Hagberg et al., 2015).

**FIGURE 1:**
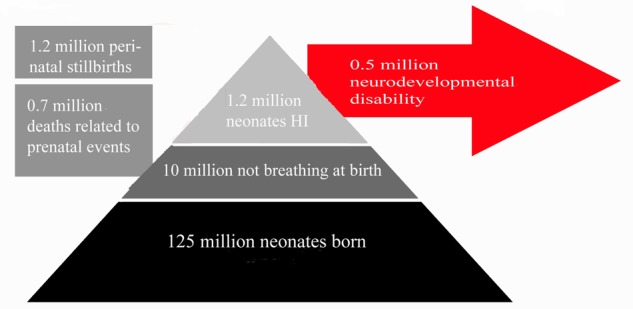
**Summary of Clinical Impact of Neonatal HI.** This figures summarizes the number of neonates affected by neonatal HI annually across the globe relative to the number of live births per annum. Estimated figures for persistent disability associated with neonatal HI are also included. Estimates are based on data from studies references in this review (Hack et al., 1992; Vohr et al., 2000; Dilenge et al., 2001; Volpe, 2001, 2012; Graham et al., 2008; Lee et al., 2013).

Neonatal HI is the most common cause of death and disability in human neonates (Grow and Barks, 2002; Ferriero, 2004; Shalak and Perlman, 2004), accounting for 23% of infant mortality worldwide, and affecting 0.7–1.2 million infants annually (Lawn et al., 2005; see **Figure 1**). In developed countries, incidence of HI injury has not decreased in the past two decades (Himmelmann et al., 2005; Vincer et al., 2006), remaining a significant cause of fatality and disability. The frequency of motor and cognitive disorders linked to perinatal and early postnatal brain injury actually increased during the 1990s, and currently remains stable (Vincer et al., 2006; Robertson and Iwata, 2007; Wilson-Costello et al., 2007). Progress in assisted respiratory and intensive care technology has led to greater than 90% survival of infants born after gestational week 23 (Larroque et al., 2004; Fellman et al., 2010), perhaps accounting for the increased burden of disability within the population as mortality rates have decreased substantially.

Amongst those who survive the initial injury, rates of disability remain high throughout life. Of patients surviving neonatal HI, 5–10% of infants demonstrate persistent motor deficits, and 20–50% display sensory or cognitive abnormalities that persist to adolescence (Hack et al., 1992; Vohr et al., 2000; Volpe, 2001, 2012; Lee et al., 2013). A meta-analysis of seven studies including 386 infant patients investigated the average incidence of mortality and morbidity: 5.9% of all patients across all studies died, 16.3% suffered neonatal seizures, and 17.2% experienced neurological deficits, with 14.2% qualifying for a diagnosis of cerebral palsy (Graham et al., 2008). Long-term outcomes in neonatal asphyxia infants have also been investigated. One meta-analysis found that 1–18% of patients were identified as having severe sensorimotor or learning disorders by the age of 2–5 years, with only 50–60% of patients reported as developmentally normal (Dilenge et al., 2001). This study covered a wide range of injury severities and follow-up ages. Disorders included seizures, hearing and vision loss (Robertson and Finer, 1985), language disorders, microcephaly, and muscle spasticity (Shankaran et al., 1991, 2012). Studies also report more severe neurological signs in patients suffering severe HI compared to those with milder HI injury (Robertson and Finer, 1985). Yet, many individuals who showed abnormal neurological signs at birth were normal at 2-year follow-up (De Souza and Richards, 1978). Clinical features and outcomes of neonatal HI are summarized in **Figure 1**.

Despite the high disability burden associated with surviving neonatal HI patients, there are very few preventative or protective treatments available for infants suspected to have suffered an HI event. The only licensed treatment currently available is hypothermia. This treatment involves subjecting either the infant’s whole body or head-only to temperatures of around 33°C (Choi et al., 2012; Tagin et al., 2012). Hypothermia was first demonstrated to improve survival in cases of cardiac arrest (Bernard et al., 2002; Nikolov and Cunningham, 2003), and has since been applied as a neuroprotective treatment in acute neonatal HI injury patients (Gunn et al., 1997, 2005; Gunn and Gunn, 1998; Shankaran et al., 2002, 2005; Eicher et al., 2005; Gluckman et al., 2005; Jacobs et al., 2005, 2013; Shah et al., 2007; Azzopardi et al., 2009; Shah, 2010). A recent meta-analysis found that hypothermia carried out in over 1,200 infants reduced the rate of death and neurological handicaps at 18 months follow-up across all severity categories of neonatal HI injury (Tagin et al., 2012). However, hypothermia alone is not sufficient to prevent all brain injury or neurological symptoms, highlighting the need for additional therapies to use in conjunction. Xenon gas administration is currently being trialed as an additive therapy alongside hypothermia (Hobbs et al., 2008; Thoresen et al., 2009; Johnston et al., 2011). There are currently no other licensed treatments available for neonatal HI.

Despite the efficiency of hypothermia (Tagin et al., 2012; Jacobs et al., 2013; Pauliah et al., 2013; Srinivasakumar et al., 2013; Kracer et al., 2014; Kali et al., 2015), death and disability remain a common feature of neonatal HI prognosis. Observations concerning global prevalence and poor long-term outcome reiterate the urgency of finding novel neuroprotective treatments for use during and directly following HI injury. This review examines the neuropathology resulting from neonatal HI injury in humans. The review then examines currently available animal models of neonatal HI and summarizes the strengths and weakness of such models for research into this complex human condition. Finally, the review will detail a potential approach toward identifying new pharmacological targets for neonatal HI therapies, focusing on the protein neuroserpin.

## Neurobiology of Neonatal Hypoxia Ischaemia in Humans

There is ample evidence that brain damage occurs in human neonatal HI patients with poor clinical outcomes, documented in both imaging and histopathological studies (Mortola, 1999; Volpe, 2008, 2012; Northington et al., 2011). Neuropathology has been characterized in human post-mortem studies, concluding that most areas of the brain are vulnerable to some extent to neonatal HI injury (Miller et al., 2005; Triulzi et al., 2006; Billiards et al., 2008; Cohen and Scheimberg, 2008; de Vries and Groenendaal, 2010; Volpe, 2012). Gray and white matter lesions have been described at term after HI (Eken et al., 1994; Marin-Padilla, 1996, 1997, 1999). Localization and extent of neuropathology has been shown to be associated with neurodevelopmental symptoms, giving insights into the nature of disability the patients may present with.

### Neuroanatomy: Structural Imaging

**FIGURE 2:**
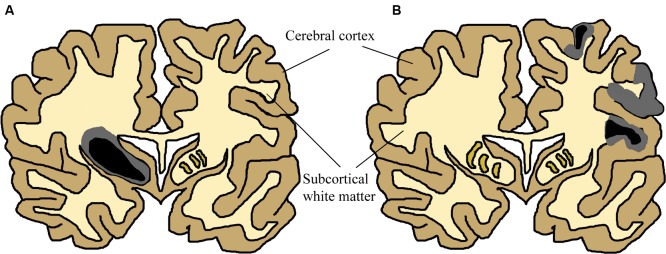
**Simplified Schematic of Brain Damage in Neonatal HI, approximately at the level of primary somatosensory and motor cortex.** The two main patterns of injury, partially overlapping in patients, are shown separately for this schematic (adapted from Budday et al., 2014). Two colours have been used to show that many neonatal HI injuries consist of a centre of necrosis (black) and a penumbra of less acutely damaged tissue (gray). The exact location of these sites will vary depending on the nature of the injury. Black/gray areas represent the potential site of lesions, although those shown in this schematic are severe yet unilateral. **(A)** Primary basal-ganglia and thalamus injury pattern. **(B)** Primary watershed cortex and underlying white matter injury. Injury can primarily occur either to cortical gray matter or subcortical white matter depending on the nature of the injury. Severity also varies substantially between patients and within the brain of individuals. These have been documented in many human structural imaging studies (Barkovich et al., 1995; Cowan et al., 2003; Kaufman et al., 2003; Rutherford et al., 2004; Miller et al., 2005; Chau et al., 2008, 2012, 2013; Li et al., 2009).

Infants who survive the initial HI insult display cerebral damage visible with structural imaging. Magnetic resonance imagining (MRI) studies of term infants with neurological signs and combinations of fetal distress, cord acidemia, and depressed Apgar scores have been carried out in over 1,000 infants (reviewed in Volpe, 2012). These studies report great variation in the anatomical areas involved between individual patients, yet most samples described either patients demonstrating predominant or substantial injury to cerebral cortex (Barkovich et al., 1995; Rutherford et al., 2004; Miller et al., 2005; Chau et al., 2008, 2012, 2013; Li et al., 2009), or basal ganglia and thalamus (Barkovich et al., 1995; Rutherford et al., 1998, 2004; Cowan et al., 2003; Kaufman et al., 2003; Miller et al., 2005; Chau et al., 2008) in partially overlapping subpopulations. These two patterns of injury are shown in **Figure 2**. Cerebral white matter has been described as selectively sensitive to term HI injury (Inder et al., 1999; Craig et al., 2003). Although less common, severe selective involvement of subcortical white matter has been documented (Neil et al., 2002; Vermeulen et al., 2003). One review described the literature concerning structural MRI scans in the acute phase (within 2 weeks after birth): approximately 15–30% of scans were normal, lesions in basal ganglia and thalamus are present in 40–80% of cases, with abnormalities of watershed white matter and cortex present in 40–60% of patients (Volpe, 2012). MRI anatomy has been shown to agree well with post-mortem studies (Cowan et al., 2003). Therefore, no single area of the brain is specifically damaged following neonatal HI. Any future treatments should take this diversity into account and provide neuroprotection to neurons throughout the brain.

The MRI scan is currently the method of choice for investigation of neonatal anatomy in both clinical and experimental circumstances (Perrin et al., 1997; Ment et al., 2002; Li et al., 2009). Diffusion-weighted MRI imaging has greatly improved identification of the time of onset of brain lesions (L’Abee et al., 2005; Chau et al., 2009). A reduced diffusion coefficient can be calculated, showing restricted diffusion during the first few days after the insult, with pseudonormalization by the end of the first week (McKinstry et al., 2002; Malik et al., 2006; Liauw et al., 2009). Sequential imaging has shown that lesions in the basal ganglia may increase in size during the first week after birth (Soul et al., 2001; Barkovich et al., 2006), and asymmetric diffusion within white matter has been correlated with clinical severity of hemiparesis (Glenn et al., 2007). Cranial ultrasound also remains a valuable clinical tool (Daneman et al., 2006). Another technique promising to add to understanding of neonatal HI is magnetic resonance spectroscopy (MRS), which allows brain metabolism to be imaged in real time (Kemp and Radda, 1994; Soares and Law, 2009). Full-term neonates with perinatal asphyxia have been studied, indicating that brain metabolism becomes abnormal after 6 to 12 h only to decrease even further after 24 h (Wyatt et al., 1989; Moorcraft et al., 1991; Roth et al., 1992). This coincided with clinical deterioration such as development of seizures. The concept of a delayed metabolic abnormality or ‘secondary energy failure’ has been elaborated in animal models (Lorek et al., 1994; Penrice et al., 1997; Groenendaal et al., 2006). Using MRS in these animal models, neuroprotective strategies could be tested. By using MRS data as real-time measurements of decreased brain metabolism in brain injury. ^1^H-MRS and ^31^P-MRS studied have demonstrated metabolic abnormalities following HI insult, which may persist for weeks (Groenendaal et al., 1994; Robertson et al., 1999). Unfortunately, with the magnetic field strength of current clinical systems, only large brain areas can be examined, limiting the use of this technique. Infants who have suffered neonatal HI often exhibit abnormal EEG activity (reviewed in Walsh et al., 2011; van Laerhoven et al., 2013). A range of abnormalities have been described, including: low voltage in isoelectric EEG (Finer et al., 1983; Legido et al., 1991), mild voltage depression (Watanabe et al., 1980; Toet et al., 2002; Murray et al., 2010), and asymmetry of trace (Aso et al., 1989; Zeinstra et al., 2001; Murray et al., 2009), although all of these criteria have been differently defined by different analysts (Rennie et al., 2004; Shellhaas et al., 2007). However, imaging techniques are constantly improving.

Lesions occur in many clinical patients, yet the effect on cognitive function is as diverse as the neuroanatomy. The area of cortex and basal ganglia damaged during the initial HI injury is directly predictive of language and motor outcome in childhood (Steinman et al., 2009; Martinez-Biarge et al., 2011). Examination of diffusion-tensor imaging of neonates was predictive of survival and motor outcome (Hunt et al., 2004; Ward et al., 2006). Children with basal-ganglia-thalamus pattern of injury tend to be severely disabled due to dyskinetic cerebral palsy and epilepsy (Himmelmann et al., 2007). Infants with predominant watershed white matter and cortex lesions have more prominent cognitive than motor deficits (Miller et al., 2005; Gonzalez and Miller, 2006; Steinman et al., 2009). Severe motor impairment is uncommon, and this group is often considered to have a normal outcome when seen at 12–18 months, although suboptimal head growth, behavioral problems, epilepsy, and a delay in language emerge during late childhood (Mercuri et al., 2000; Miller et al., 2002; Oguni et al., 2008; Sato et al., 2008; Steinman et al., 2009). Therefore, cortical damage appears to be relevant to functional outcome in surviving neonatal HI patients. This information could be used to predict future susceptibility to disability before it manifests, allowing social and educational supports to be put in place early.

### Molecular Mechanisms of Cell Death

Anatomical studies describe loss of brain volume following moderate and severe neonatal HI. However, the underlying molecular mechanisms responsible for cell death are debated (McLean and Ferriero, 2004; Fatemi et al., 2009; Northington et al., 2011; Baburamani et al., 2012). Many pathways have been implicated in HI injury in the term brain, primarily: excitotoxicity, oxidative stress, and inflammation. Molecular studies have drawn attention to a fact essential for the development of successful new therapies that the neonatal brain and its injury is fundamentally different from that seen in adult HI stroke injury (McLean and Ferriero, 2004; Johnston et al., 2011; Baburamani et al., 2012; Semple et al., 2013).

There are many important differences between neonatal HI and adult ischaemic stroke. For example, severe HI events in the infant brain can lead to liquifactive disintegration, not seen after adult stroke (Larroche, 1977; Rorke, 1992). Newly formed blood vessels are fragile and prone to rupture (Trommer et al., 1987; Volpe, 1989; Ment et al., 1991; Jones et al., 2002), and surrounded by fewer astrocyte end-feet (El-Khoury et al., 2006). Another key site of difference is the BBB. Studies in rodents indicate that the BBB is compromised as a result of neonatal HI (Muramatsu et al., 1997; Svedin et al., 2007; Ferrari et al., 2010; Tu et al., 2011; Yang et al., 2012). Yet the common belief that the neonate BBB is less effective has recently come under revision (Saunders et al., 1999, 2012, 2014; McLean and Ferriero, 2004; Baburamani et al., 2012; Stolp et al., 2016). Tight junctions, the occlusive element of the BBB, are present as soon as embryonic vessels invade the brain (Schulze and Firth, 1992; Bauer et al., 1993; Stewart and Hayakawa, 1994; Kniesel et al., 1996), and are functional (Ek et al., 2003, 2006; Daneman et al., 2010). In a model of hypoxia in newborn piglet, BBB integrity was maintained (Stonestreet et al., 1992), yet other experiments have demonstrated damage to the BBB following neonatal HI (Alvarez-Diaz et al., 2007; Leonardo and Pennypacker, 2009).

Cerebrovascular autoregulation is another factor which must be considered in neonates. The concept that preterm infants have a ‘pressure passive’ cerebral circulation is widely accepted. However, sick term infants demonstrate impaired autoregulation (Pryds et al., 1990; Hardy et al., 1999; Boylan et al., 2000) and the range of blood pressure over which cerebrovascular autoregulation functions expands with maturity (Tuor and Grewal, 1994; Verma et al., 2000). Also, the concentrations and actions of various signaling molecules is different in the developing brain including; caspase-3 (Cheng et al., 1998), VEGF (Carmeliet and Storkebaum, 2002), and HIF-1 (Iyer et al., 1998), among others (reviewed in Baburamani et al., 2012).

One surprising difference is sexual dimorphism in response to neonatal HI. Male babies are at higher risk of cerebral palsy than females (Jarvis et al., 2005). Cognitive and motor outcomes are worse in male than in female low birth weight infants (Johnston and Hagberg, 2007). Quantitative imaging shows that male premature infants are more vulnerable to white matter injury, whereas females are more vulnerable to gray matter injury (Thompson et al., 2007). This sex difference has also been replicated in rodent *in vitro* models of hypoxic cell death (Zhu et al., 2006; Nijboer et al., 2007; Du et al., 2009). Although many molecular mechanisms are currently under investigation, this sexual dimorphism remains largely unexplained (Hill and Fitch, 2012; Chavez-Valdez et al., 2014; Demarest et al., 2016a,b; Waddell et al., 2016). Therefore, the unique state of the developmental brain should always be at the forefront of the researcher’s minds.

Neonatal HI injury evolves over time (McLean and Ferriero, 2004). Injuries seen with MRI scans within the first few hours after asphyxia are subtle, restricted diffusion typically starting as small lesions in the putamen and thalami, progressing over the next 3 to 4 days to involve more extensive areas of the brain (Takeoka et al., 2002). Within the first few hours, regionally specific fluctuations in blood flow trigger excitotoxicity, free radical generation, and edema (Wigglesworth and Pape, 1978; Bennet et al., 1998; Jensen et al., 1999; Shalak and Perlman, 2004; Ferrari et al., 2010). A secondary phase of injury occurs during the following hours and days, resulting in neuroinflammation, mitochondrial permeabilization, and loss of cerebral autoregulation (Inder and Volpe, 2000; Hamrick and Ferriero, 2003; Scheepens et al., 2003; Hagberg et al., 2009; Leonardo and Pennypacker, 2009). A tertiary phase of brain injury has been proposed, which may exacerbate injury through persistent inflammation (Fleiss and Gressens, 2012).

The balance between molecular cell-death processes which cause this damage in neonatal HI remains debated. Early evidence indicates that the majority of cell death in neonatal HI is necrotic, however, all regions also undergo increased apoptotic death (Edwards and Mehmet, 1996; Edwards et al., 1997; Northington et al., 2001). Some studies suggest a more prominent role for apoptosis (Hill et al., 1995; Sidhu et al., 1997; Pulera et al., 1998; Hu et al., 2000; McLean and Ferriero, 2004). Immature neurons *in vitro* are more susceptible to apoptotic death than mature neurons (McDonald et al., 1997). Others report that necrosis is the major cellular pathology in humans and animals (Adamsons and Myers, 1973; Myers, 1975; Towfighi et al., 1995; Northington et al., 2001, 2005, 2011; Folkerth, 2005; Carloni et al., 2007; Stridh et al., 2013). Yet others recognize that both occur. Some report that necrosis predominates in severe cases, whereas apoptosis occurs in milder injury (Stroemer and Rothwell, 1998; Daval and Vert, 2004; Fatemi et al., 2009). Neurons often display morphologic features along an apoptosis-necrosis continuum (Portera-Cailliau et al., 1997a,b; Nakajima et al., 2000; Northington et al., 2007, 2011). In addition to apoptosis and necrosis, some neurons in the neonatal HI brain undergo autophagy (reviewed in Klionsky and Emr, 2000; Northington et al., 2011; Balduini et al., 2012). Neuronal autophagy occurs in rodent neonatal HI models (Lockshin and Zakeri, 1994; Carloni et al., 2008; Ginet et al., 2009). However, there is conflicting evidence as to whether the occurrence of autophagy augments brain damage (Koike et al., 2008; Puyal et al., 2009), or prevents the spread of necrotic cell death (Carloni et al., 2008). Artificially exclusive classification of cell death may hinder research and therapy development.

**FIGURE 3:**
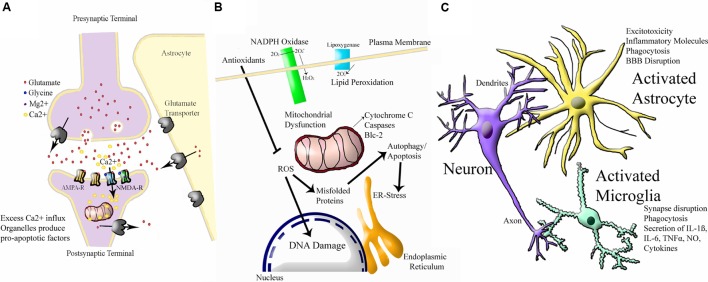
**Simplified Schematic of Major Molecular Injury Cascades involved in Neonatal HI.** A vast number of molecules contribute to neonatal HI injury in the brain and all of these have been investigated in this disorder. These molecular targets can largely be split into the following three cascades. **(A)** Excitotoxicity (adapted from Vandenberg and Ryan, 2013) Ca^2+^ = calcium ion, Mg^2+^ = magnesium ion AMPA-R = α-amino-3-hydroxy-5-methyl-4-isoxazole propionic acid receptor, NMDA-R = *N*-methyl-D-aspartate receptor. **(B)** Oxidative Stress (adapted from http://www.enzolifesciences.com/platforms/cellular-analysis/oxidative-stress/). For simplicity, only free radicals are shown and physiological pathway substrates have been omitted. NADPH + Nicotinamide Adenine Dinucleotide Phosphate Hydrogen, O_2_ = oxygen, O_2_^-^ = negatively-charged oxygen free radiccal, H_2_O_2_ = hydrogen peroxide, ROS = ROS1 receptor tyrosine kinase encoded by the ROS1 gene, Blc = B cell leukaemia protein, ER = endoplasmic reticulum, DNA = deoxyribose nucleic acid. **(C)** Inflammation (adapted from Santiago et al., 2014). BBB = blood brain barrier, IL-6 = interleukin 6, IL-1B = interleukin 1 beta, TNFalpha = tumour necrosis factor alpha, NO = nitric oxide.

To add to this complexity, neonatal HI injury appears to activate several interacting molecular cascades. A simple schematic of the three major cascades is shown in **Figure 3**. The first is excitotoxicity, through which physiological glutamate neurotransmission leads to overactivation of postsynaptic receptors and cell death (reviewed in Hagberg et al., 1987; Choi, 1988, 1992; Hattori and Wasterlain, 1990; Danbolt, 2001). The *N*-methyl-D-aspartate (NMDA) receptor is relatively over-expressed in the developing brain (McDonald et al., 1989a; Represa et al., 1989; Fox et al., 1996). In P6 rats, the NMDA receptor is expressed at 150–200% of adult levels (Tremblay et al., 1988). The predominating combination of NMDA receptor subunits in the perinatal period seems to favor prolonged calcium influx for a given excitation (Danysz and Parsons, 1998). The same NMDA receptor that promotes plasticity can lead to massive Na^+^ and water influx, cellular swelling, pathologically elevated intracellular calcium, and energy failure, leading to a ‘spiral of death’ (Choi, 1988). Oxygen glucose deprivation (OGD) in rat hippocampal neurons leads to a marked reduction in glutamate removal from the synapse (Jabaudon et al., 2000; Tao et al., 2001). Injection of NMDA into rat brain produces more extensive cell death in the neonate than in the adult (McDonald et al., 1988). Elevated glutamate has been documented in the cerebrospinal fluid (CSF) of infants who have suffered severe HI injury (Riikonen et al., 1992; Hagberg et al., 1993; Pu et al., 2008). The neonatal brain is much more prone to seizure activity than the mature brain (Holmes, 1991; Holmes and Ben-Ari, 2001), suggesting a prominent role for neuronal hyperexcitability and excitotoxicity although the molecular mechanisms behind this have not been fully elucidated (reviewed in Rakhade and Jensen, 2009). However, seizure activity could also be explained by paradoxical excitatory activity of the neurotransmitter gamma-amino butyric acid (GABA) in the developing brain (Staley et al., 1995). Drugs that block NMDA receptors are protective against HI injury in neonatal rodent models (McDonald et al., 1989a,b, 1990). Activation of AMPA receptors also contribute to injury (McDonald and Johnston, 1992; Deng et al., 2003; Talos et al., 2006), however, AMPA antagonists are not as protective (Ikonomidou et al., 1999; Noh et al., 2006). These findings are yet to be exploited in human clinical trials, as the integral role of glutamate receptors in healthy neuronal plasticity (Ikonomidou et al., 1999; Failor et al., 2010; Rocha-Ferreira and Hristova, 2015) could be damaged by the use of NMDA and AMPA antagonists at such a sensitive developmental stage.

An integrally linked cascade is that of oxidative stress. Excitotoxicity causes energy depletion, mitochondrial dysfunction, and cytosolic calcium accumulation, which in turn leads to generation of free radicals (Ferriero et al., 1996; Ferriero, 2001). Free radicals alter membrane pump function, allowing more glutamate release and NMDA receptor activation, leading to more excitotoxicity (Schanne et al., 1979; Robertson J.D. et al., 2002; Starkov et al., 2004). Oxidative stress is a general term for the increase in free radical production as a result of oxidative metabolism under pathologic conditions (Inder and Volpe, 2000; Ferriero, 2001). When oxygen floods the microenvironment of cells damaged by hypoxia, mitochondrial oxidative phosphorylation is overwhelmed and reactive oxygen species accumulate (Ferriero, 2001). Fetal life elapses in a low oxygen environment (East et al., 1998). In the first minutes of life, an abrupt increase in O_2_ partial pressure occurs, which creates a pro-oxidant condition (Stiller et al., 2002). During birth asphyxia, excess calcium influx and other factors lead to severe oxidative stress (Forder and Tymianski, 2009). There is accumulation of hydrogen peroxide after HI in neonatal mice but not in adults (Lafemina et al., 2006). Because of its high lipid content, the brain is particularly susceptible to free radical attack (O’Brien and Sampson, 1965; Northington et al., 2001). The polyunsaturated fatty acid content of the brain increases during gestation (Crawford and Sinclair, 1971; Mishra and Delivoria-Papadopoulos, 1989). Lipid peroxidation may be a major factor in the white matter damage (Back et al., 1998; Baud et al., 2004). The developing brain’s immature antioxidant defense also contributes to sensitivity to oxidative stress (Li et al., 1997; Mishra and Delivoria-Papadopoulos, 1999; Li and Jackson, 2002; Felderhoff-Mueser et al., 2002; Vannucci and Hagberg, 2004; Blomgren and Hagberg, 2006; Ikonomidou and Kaindl, 2011; Miller et al., 2012). Adequate stores of antioxidants are necessary to protect against oxidative injury. Specifically, depletion of neuronal reduced glutathione exacerbates oxidative injury (Chen and Liao, 2003; White and Cappai, 2003; Brongholi et al., 2006).

Finally, inflammation is a major component of neonatal HI injury. Low-dose treatment with intrauterine LPS dramatically increases severity of HI injury in neonatal mice, but protects against HI in adult rodents (Wang et al., 2007b). Intracerebral injection of NMDA receptor agonist produces a pattern of white matter injury, in which pretreatment with systemic IL-1ß, IL-6, IL-9, or TNF-α leads to a significant increase in lesion size (Marret et al., 1995; Dommergues et al., 2000). There is now substantial experimental evidence that intrauterine inflammation can exacerbate neonatal HI (Lehnardt et al., 2003; Eklind et al., 2005; Marini et al., 2007), which some have referred to as the “double-hit hypothesis” (reviewed in Agrawal and Hirsch, 2012; Hagberg et al., 2012; Dammann and Leviton, 2014). Microglia, the resident macrophages of the CNS, are among the first cells to become activated after HI (Fujimoto et al., 1989; Tahraoui et al., 2001; Kaur et al., 2007). Activated microglia migrate to damaged regions (Leonardo and Pennypacker, 2009) and produce inflammatory cytokines, glutamate, nitric oxide, and free radicals (Wood, 1995; Kaur and Ling, 2009). Drugs that block microglial activation protect the neonatal brain (Dommergues et al., 2003). Following hypoxia-ischemia, compromise of the BBB allows the entry of macrophages (Alvarez-Diaz et al., 2007; Leonardo and Pennypacker, 2009). Astrocytes also play a role in inflammation (Wang et al., 2003; Girard et al., 2008, 2009). CSF cytokines are elevated in term infants who later develop cerebral palsy (Savman et al., 1998; Dammann and O’Shea, 2008). The diverse network of interacting mechanisms demonstrate the molecular complexity of neonatal HI injury. Potential protective treatments should strive to tackle common mediators of these cascades relevant to all three pathways, otherwise full protection will not be achievable.

## Evaluation of Available Animal Models of Neonatal Hypoxia Ischaemia

**FIGURE 4:**
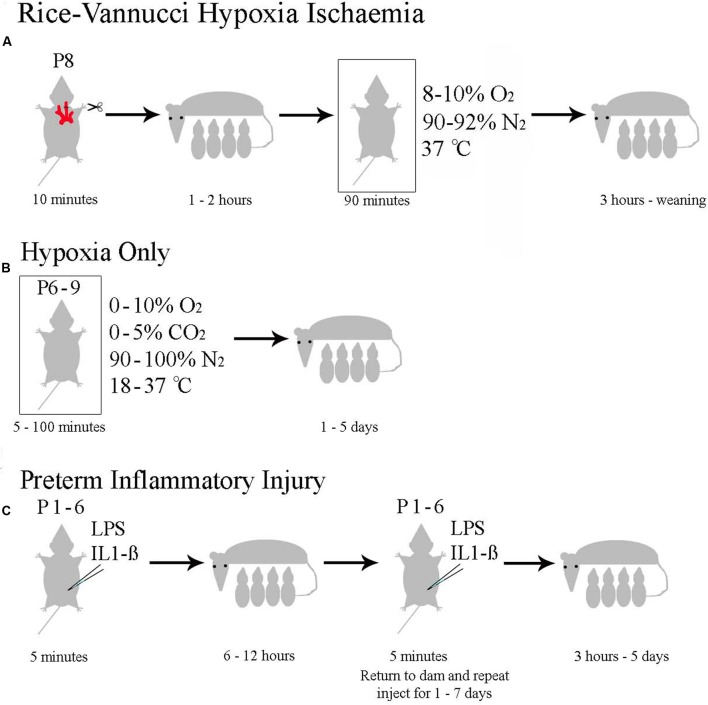
**Currently Used Rodent Models of Neonatal HI.** **(A)** The Rice–Vannucci method as outlined in rats by Rice et al. (1981). **(B)** The general method with specifications taken from the pool of papers summarized in Supplementary Table 1. **(C)** The range of inflammatory models reviewed in Dean et al. (2015).

Neonatal hypoxia ischaemia has been modeled extensively in mice and rats (reviewed in Hagberg et al., 2002; van der Worp et al., 2007; Yager and Ashwal, 2009; Dean et al., 2015), with a minority of researchers also studying larger animals such as pigs, sheep (reviewed in Roohey et al., 1997; Dean et al., 2015) or primates (Fahn et al., 1979; Volpe, 2012). Models intending to replicate the clinical symptoms of neonatal human HI can be roughly divided into the four categories discussed below. All models have distinct advantages and disadvantages. A summary of available rodent models is shown in **Figure 4**.

### Rice–Vannucci Model of Term Hypoxia Ischaemia

Most published studies modeling neonatal HI in animals have employed the Rice–Vannucci model (Rice et al., 1981). This model comprises unilateral carotid artery ligation, recovery with the dam for approximately 1 h, followed by exposure to 8% oxygen for 1–3 h at 37°C. Although, the model was initially described in rat (Rice et al., 1981), it has been successfully adapted for mouse with similar anatomical and behavioral effects (Ditelberg et al., 1996; Ferriero et al., 1996; Sheldon et al., 1998).

Rice’ and Vannucci’s model replicates anatomical damage seen in human neonates. Their initial study showed selective gray-matter sensitivity to neuronal necrosis, with gray matter injury observed in cortex, hippocampus, thalamus, and basal ganglia (Rice et al., 1981; Andine et al., 1990; Towfighi et al., 1995; Vannucci et al., 1999), encompassing the sites damaged in human neonatal HI. Histologically, there is a gradation of injury that correlates with the duration or severity of insult (Towfighi et al., 1991, 1995). White matter lesions have also been described in this model (de Torres et al., 1997; Ness et al., 2001; Liu et al., 2002; Drobyshevsky et al., 2005, 2007b), the extent of which correlate with the duration of exposure to hypoxia (Liu et al., 2002). However, bilateral common carotid artery ligation appears a stronger model of white matter damage (Jelinski et al., 1999; Uehara et al., 1999; Cai et al., 2001). Metabolic alterations in the Rice–Vannucci model include decreased cerebral blood flow (Sakurada et al., 1978; Vannucci et al., 1988), brain acidosis (Welsh et al., 1982; Yager et al., 1991), and decreased cerebral glucose uptake (Vannucci et al., 1989; Sokoloff et al., 1977). An inflammatory response has also been demonstrated (Bona et al., 1998).

Another convincing aspect of the Rice–Vannucci model is its ability to predict the therapeutic effect of hypothermia following the neonatal HI event. Mice treated with hypothermia showed smaller lesion volumes, in addition to better performance on the Morris water maze and circling tests (Yager et al., 1993; Lee et al., 2010; Kida et al., 2013; Lin et al., 2014). Many papers have investigated the behavioral outcomes of Rice–Vannucci injury in adult rodents. This model gives rise to well documented behavioral phenotypes including: impaired spatial learning and memory (Balduini et al., 2000, 2001; Ikeda et al., 2001; Wang et al., 2002; Arteni et al., 2003; Pereira et al., 2007; Cai et al., 2009; Greggio et al., 2011; Hill et al., 2012; Zheng and Weiss, 2013; Alexander et al., 2014; Gillani et al., 2015); impaired motor function as assessed by rotarod test, open field and motor reflexes (Barth and Stanfield, 1990; Jansen and Low, 1996a,b; Jansen et al., 1997; Balduini et al., 2000, 2001; Tomimatsu et al., 2002; Ådén et al., 2003; Lubics et al., 2005; Pazaiti et al., 2009; Im et al., 2010; Nijboer et al., 2010; Karalis et al., 2011; Chen et al., 2012; Ruiz et al., 2012; Sanches et al., 2012; Zheng et al., 2012; Xiong et al., 2013; Alexander et al., 2014; Gillani et al., 2014, 2015; Kim et al., 2014; Zhang Q. et al., 2014; Park D. et al., 2015; Park W.S. et al., 2015); sensory processing abnormalities (Alexander et al., 2014); and other cognitive phenotypes, such as reduced attention (Buwalda et al., 1995; Martin et al., 1997; Sanches et al., 2013; Perera et al., 2014; Miguel et al., 2015). Despite this broad range of documented effects, there are some contradictions between individual investigators (reviewed in Lubics et al., 2005), which suggest that different genetic backgrounds, severity, or experimenters can significantly affect the outcome of Rice–Vannucci model.

The Rice–Vannucci model of neonatal hypoxia ischaemia has several advantages. One is its prevalence, allowing direct comparisons with many other published papers (Vannucci et al., 1993, 2005; Yager and Ashwal, 2009; Dean et al., 2015). Another is that the contralateral hemisphere, exposed to hypoxia in the absence of ischemia, appears normal (Yager et al., 1991, 1992, 1996; Vannucci and Yager, 1992), providing a control hemisphere within the experimental brain. Thorough behavioral characterization (Arteni et al., 2003; Lubics et al., 2005) support the long term consequences of this model mimicking neonatal HI. One significant drawback of this model is the high variability in size and severity of infarct between animals, making comparisons between experimenters difficult (Vannucci and Hagberg, 2004; Vannucci and Vannucci, 2005). Additionally, the invasive nature of severing the common carotid artery does not replicate human injury; such severe vascular abnormalities occur rarely, if at all (Ment et al., 1984; Hill, 1991).

### Hypoxia-Only Models

Some experimenters induce hypoxia in rodents exclusively using an oxygen deprivation chamber, without a preceding ischaemic procedure. These models are not as widely used as the Rice–Vannucci method, but have the potential to describe milder injuries and avoid the unphysiological occlusion of the common carotid artery. There are currently no published reviews or meta-analyses of hypoxia-only investigations, Supplementary Table 1 contains a brief summary of 122 published papers using hypoxia-only methodology compiled from Pubmed search results.

Historically, these methods have been used to investigate hypoxic brain biochemistry. Several studies have documented altered levels of neurotransmitters (Hedner et al., 1980; Kaneko et al., 1985; Yamamoto et al., 1985; Yamamoto and Kato, 1986; Hadjiconstantinou et al., 1990; Seidler and Slotkin, 1990; Dell’Anna et al., 1993; Tanaka et al., 1995; Anju et al., 2010a,b; Anju and Paulose, 2011, 2013). However, there is little consensus over the direction or magnitude of changes (Decker M.J. et al., 2003; Decker et al., 2005). Hypoxia-only models have become an established model used to generate seizures in neonatal rats (Jensen et al., 1995; Applegate et al., 1996; Rodríguez-Alvárez et al., 2015; Sampath et al., 2015). In one such model, P6 rat pups were placed in chambers at 9% O_2_ partial pressure and 20% CO_2_ partial pressure for 60 min. Some pups were immediately restored to room air, whereas others underwent gradual reduction of CO_2_ (Helmy et al., 2011; Tolner et al., 2011). Pups which underwent hypoxia with immediate restoration of CO_2_ had a greater mortality rate and higher seizure frequency. However, subsequent anatomical analysis of these brains at P8 (Boss et al., 2005; Wang et al., unpublished), failed to show any differences in expression of cell death markers or layer-specific markers of healthy cortical neurons. Therefore, the hypoxia-only insult resulting in seizures appears to generate only subtle injury to the brain, far short of that seen in some human patients.

A range of behavioral phenotypes have been reported in hypoxia-only models, including hyperactivity (Shimomura and Ohta, 1988; Dell’Anna et al., 1991; Speiser et al., 1991; Decker et al., 2005); increased aggression (Mikati et al., 2005; Tang et al., 2006), altered ultrasonic vocalization (Venerosi et al., 2006), and disturbed sleep (Decker M.B. et al., 2003). Relatively few investigators have pursued standard tests of spatial memory and locomotor behavior, and have obtained mixed results (Dell’Anna et al., 1991; Speiser et al., 1998; Rotstein et al., 2006; Coq et al., 2008; Raveendran and Skaria, 2013; Wang S. et al., 2015). The majority of hypoxia-only phenotypes are based on custom behavioral tests, making them difficult to compare to other established animal models. Additionally, some studies report no behavioral deficit following neonatal hypoxia alone (Buwalda et al., 1995; Iuvone et al., 1996; Casolini et al., 2005; Blaise et al., 2009; Mikhailenko et al., 2009; Anju et al., 2010c; Wang S. et al., 2015).

Although hypoxia-only models offer the potential to replicate the mechanism of hypoxia without major ischaemia seen in human neonatal HI patients, the models currently available are not ideal. One significant problem is the lack of methodological unity between different experimenters. There is little consensus on age of animal, background strain, oxygen partial pressure, time of exposure to hypoxia, or body temperature (see Supplementary Table 1). One example demonstrating the relevance of close control of these variables is temperature. P0 rat pups exposed to anoxia exhibit behavioral defects when anoxia was conducted at 39°C, yet not at 33 and 36°C (Rogalska et al., 2004, 2009; Caputa et al., 2005; reviewed in Rogalska et al., 2006). Therefore, far more care is needed to justify the design of hypoxia-only experiments before these models can be considered dependable models of human neonatal HI.

### Inflammatory Models of Perinatal Brain Injury

Intrauterine infection is strongly associated with preterm birth and brain injury (Stoll et al., 2004; Mitha et al., 2013; Strunk et al., 2014; Dean et al., 2015). Many models have been described which introduce different inflammation-inducing molecules at different ages (Dean et al., 2015), many of which cause cerebral inflammation and white matter damage seen in human patients.

Administration of live *E. coli* into the uterus of pregnant rats can result in neutrophil infiltration in the fetal brain, increased fetal reabsorption and stillbirth, while surviving pups exhibit increased brain chemokines, cytokines, white matter injury, and behavioral phenotypes (Debillon et al., 2003; Rodts-Palenik et al., 2004; Pang et al., 2005; Yuan et al., 2005; Girard et al., 2009; Bergeron et al., 2013). The effects of bacterial mimetics such as the cell wall component lipopolysaccharide (LPS), have also been investigated. Intracervical injection in embryonic day 15 (E15) mice was associated with mild white matter injury but no behavioral deficits (Bell and Hallenbeck, 2002; Poggi et al., 2005; Wang et al., 2007a), whereas repeated intracervical LPS was associated with delayed neurosensory development (Toso et al., 2005; Rousset et al., 2006, 2013). Other inflammatory models include viral infection simulated by injection of poly(I:C), a synthetic double stranded viral RNA, injection of which is associated with long-term behavioral deficits (Shi et al., 2009; Richetto et al., 2013). Postnatal administration of inflammatory agents is widely used in rodents to model postnatal infection. Subcutaneous injection of live *E. coli* to P3 mouse pups was associated with microgliosis, loss of oligodendrocytes, and impaired motor coordination (Lieblein-Boff et al., 2013). However, most techniques which employ live bacterial injection have very high mortality rates (Rodewald et al., 1992; Tran and Weisman, 2004; Placencia et al., 2009; Loron et al., 2011). Postnatal intraperitoneal injection of LPS can also cause white matter damage and cerebral cytokine response (Brochu et al., 2011; Brehmer et al., 2012; Nobuta et al., 2012; Smith et al., 2014). Repeated daily injection of LPS in mice resulted in elevated serum IL-6, reduced gray matter volume, decreased oligodendrocyte numbers, and decreased myelin staining (Wang et al., 2009; Malaeb et al., 2014). Similarly, repeated IL-1β injection in P1–P5 mice has been associated with impaired oligodendrocyte progenitor maturation, and severe memory deficits (Favrais et al., 2011).

One of the strengths of the inflammation model is that it reflects the exposure to infectious or inflammatory agents present outside of the highly sterile individually ventilated cages where many academic institutions keep experimental animals. Most of the inflammatory risk factors for increased severity of HI injury in human patients, such as maternal infection (Stoll et al., 2004; Girard et al., 2012; Dean et al., 2015), will result in systemic inflammation in addition to CNS-specific recruitment of microglia. However, there is currently debate concerning the relevance of maternal inflammation to fetal brain damage (Leviton et al., 1999; Redline and O’Riordan, 2000; Neufeld et al., 2005). The debate intensifies when fetal systemic inflammation is contrasted with neuroinflammation. Although the majority of publications cited above administer pro-inflammatory agents by intracerebral injection, some studies have administered LPS by intravenous injection (reviewed in Wang et al., 2006). In fetal sheep, intravenous LPS causes white-matter specific damage (Garnier et al., 2001, 2006; Duncan et al., 2002; Mallard et al., 2003; Peebles et al., 2003; Svedin et al., 2005), suggesting that systemic inflammation may be sufficient to trigger brain injury. However, the results of these experiments could be explained by a secondary neuroinflammatory response to systemic inflammation causing the white matter injury. Separating these parameters in future experiments will prove demanding.

Data suggest that gene expression in mouse models of inflammation closely corresponds to that seen in humans (Takao and Miyakawa, 2015), however, postnatal murine response to inflammation is likely different to that experienced by a human fetus. An important limitation of inflammatory studies is impact on cardiovascular function and the potential for secondary cerebral HI (Eklind et al., 2001; Favrais et al., 2011; Wang C.T. et al., 2015). Another is that varying results have been reported. This may be due to differences in LPS used between groups, purification method (Westphal, 1965; Lam et al., 2014), or dose (Fujihara et al., 2003). Alternatively, there is increasing evidence that the considerable individual variability in response to clinical sepsis between patients is related to host genetics (Christaki and Giamarellos-Bourboulis, 2014). Therefore, although inflammation is doubtless an important factor in neonatal brain injury, the variation between different models makes these methods difficult to evaluate.

### Alternative Models

In many ways, non-rodent models are more representative of neonatal HI as it affects human patients. Here follows a brief flavor of techniques. Relatively few studies have investigated neonatal HI in primates. Classical studies asphyxiated term monkey fetuses by covering their heads with a rubber sac and clamping the umbilical cord (Fahn et al., 1979). Fetuses resuscitated after 20 min had extremely high mortality. However, 12 min of asphyxia were required to produce any neuropathologic injury. The fetuses displayed damage predominantly within the brainstem. In a model of partial ischemia (Fahn et al., 1979), pregnant females were rendered hypotensive during the third trimester. When blood oxygen saturation was reduced to 10% for 5 h, fetuses became profoundly acidemic. At birth, these displayed opisthotonus, decerebrate posturing, and convulsions. There is one model of white-matter injury based on baboons delivered prematurely by hysterotomy (Inder et al., 2004, 2005a,b). Premature baboons were treated in an intensive care setting. Approximately half displayed white-matter injury. Analysis of behavior has not been undertaken.

More work has been carried out in fetal sheep. Umbilical cord occlusion and term asphyxia has been carried out (Gunn et al., 1992; Williams et al., 1992; Mallard et al., 1993; Richardson B. et al., 1996; de Haan et al., 1997a,b,c; Richardson and Bocking, 1998; Dalitz et al., 2003), repeated asphyxia at 5-h intervals resulted in injury to the striatum almost exclusively, whereas episodes of asphyxia repeated every 5 min caused diffuse and extensive damage to cortex, thalamus, and cerebellum in 40% of animals, and selective neuronal necrosis in the remainder. White matter injury has also been investigated (Ting et al., 1983), only those fetuses in which the mean arterial blood pressure fell below 30 mmHg exhibit brain damage, irrespective of hypoxia. Neuropathologic injury after carotid artery occlusion for 30 min caused both gray and white matter involvement, with cortical damage and selective neuronal necrosis in thalamus and striatum (Reddy et al., 1998; Petersson et al., 2002). Several laboratories have developed models in sheep after systemic endotoxemia (Duncan et al., 2002; Mallard et al., 2003). Unfortunately, no behavioral outcomes are currently available in sheep.

Finally, other small laboratory animals have been investigated. Preterm rabbit fetuses exposed to sustained placental insufficiency via intrauterine occlusion of the descending aorta displayed significant alterations in motor responses to olfactory stimuli, coordination of suck and swallow, and marked hypertonia, reminiscent of spastic quadriplegia (Yoon et al., 1997; Derrick et al., 2004, 2007; Saadani-Makki et al., 2008). Diffusion-weighted imaging detected a threshold in white matter loss below which all rabbit kits developed hypertonia, (Drobyshevsky et al., 2005, 2007a). Another promising model is that of the spiny mouse, a rodent which shows a similar level of brain development to a human neonate at birth (Brunjes, 1985, 1989, 1990; Gozzo et al., 1985; D’Udine and Alleva, 1988; Brunjes et al., 1989), which also shows varied motor deficits (Ireland et al., 2008, 2009, 2010) and neuroanatomical pathology (Hutton et al., 2009; O’Connell et al., 2013) following neonatal HI.

In brief, large animals can better replicate the conditions of a single human fetus exposed to a non-sterile environment. However, none of these models have access to the same extent of transgenic manipulation or validated behavioral tests that are possible in rodent studies. These models are invaluable for aiding comparison of brain development at birth which occur between species (Clancy et al., 2007), whereas the relative immaturity of mouse and rat brains at birth introduce an unwelcome variable into these experiments. For the foreseeable future, insights from both rodent work and larger animals will be important for better understanding neonatal HI.

## Subplate: A Region of Hyper-Sensitivity to Neonatal HI?

One brain region exemplifying how much remains unknown about the developing brain’s response to HI, and the uncertainties involved in interpreting results from animal studies, is subplate. The subplate is an early-born transitory neuronal layer of cerebral cortex which serves an important role in the establishment of thalamocortical connectivity during development (reviewed in Judas et al., 2010; Kanold and Luhmann, 2010; Kostovic and Judas, 2010; Hoerder-Suabedissen and Molnár, 2015) (see **Figure 4**). Thalamocortical axons associate with and grow alongside subplate neurons in the developing cortex (Herrmann et al., 1994; Molnár et al., 1998; Kostovic and Judas, 2006), and selective excitotoxic ablation of subplate disrupts thalamocortical connectivity in animal models (Ghosh et al., 1990; Ghosh and Shatz, 1992; Lein et al., 1999; Kanold et al., 2003; Kanold and Shatz, 2006; Magnani et al., 2013).

Subplate has been identified in a range of mammals by conserved molecular markers (Hoerder-Suabedissen et al., 2009; Hoerder-Suabedissen and Molnár, 2012; Oeschger et al., 2012; Pedraza et al., 2014). Neuronal death occurs in infant subplate during normal development (Chun et al., 1987; Al-Ghoul and Miller, 1989; Hamre et al., 1989; Kostovic and Rakic, 1990; Woo et al., 1991; Price et al., 1997). However, the molecular mechanisms behind this programmed cell-death remain unknown (McQuillen and Ferriero, 2005; Hoerder-Suabedissen and Molnár, 2015). Functional roles for subplate in thalamocortical (Allendoerfer and Shatz, 1994; Kanold and Luhmann, 2010) and corticothalamic (Grant et al., 2016) development have been described, demonstrating lasting significance of this transient population.

The susceptibility of subplate neurons to neonatal HI remains little understood. Early studies claimed subplate is selectively vulnerable. Cultured rat subplate neurons were more vulnerable to OGD compared to mixed cortical neurons (Nguyen and McQuillen, 2010). In rats exposed to systemic HI at E18, a significant decrease in Nurr1-expressing subplate neurons was documented by P2 (Jantzie et al., 2014, 2015a,b,c) and similarly in fetal sheep (Dean et al., 2011). However, Nurr1-expressing Layer VI neurons were also decreased. It is also possible that HI induced differential marker expression in subplate neurons, rather than cell death. *In vitro* electrophysiological recordings in neocortical slices from newborn rats have demonstrated a pronounced functional impairment of subplate neurons following OGD (Albrecht et al., 2005). Another influentiential study that claimed selective vulnerability of subplate to hypoxia used immunohistochemistry to detect cell-death in neurons labeled with BrdU at E10.5 following a modified Rice–Vannucci model in P1 rats (McQuillen et al., 2003; McQuillen and Ferriero, 2004, 2005). However, Layer VI neurons born at this date also expressed cell-death markers, suggesting subplate may not be selectively susceptible to HI injury. Conversely, a systematic quantification of cell death markers throughout subplate compared with other cortical layers was conducted using immunohistochemistry (Okusa et al., 2014). Three subplate-specific marker positive populations showed little co-staining with caspase-3 in brains with mild to moderate HI lesions. In severely damaged cases, caspase-3 staining was found throughout the cortex. Therefore, layer-specific sensitivity of cortical neurons to HI remains debated. A double birthdating study, targeting subplate and layer V or VI combined with HI could resolve this issue. However, such an experiment has not yet been performed.

**FIGURE 5:**
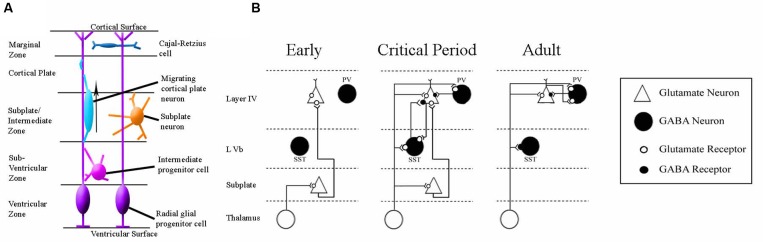
**Schematics of the anatomy and physiology of human subplate development.** **(A)** Schematic coronal section showing the major cellular compartments within the developing human cortex at 26 post-conception weeks, reviewed in Hoerder-Suabedissen and Molnár (2015). The germinal zone (site of cell division) consists of the ventricular zone and subventricular zone. The subplate and intermediate zone lie between the germinal zone and the cortical plate (the site into which the permanent layers will migrate). The outermost layer is the early-born marginal zone. **(B)** Classical schematic of subplate architecture throughout development. In the earliest phase of thalamocortical circuit establishment subplate neurons (white) receive inputs from thalamus, and project axons to layer 4. At the onset of critical period, both subplate neurons and thalamus project to layer 4. In adult, subplate neurons have been eliminated by programmed cell death and layer 4 neurons receive inputs directly from thalamus. Adapted from Kanold (2009). This classical view has been augmented by the inclusion of GABA-ergic layer Vb interneurons which have been demonstrated to contribute to thalamocortical circuitry development in somatosensory mouse cortex (Marques-Smith et al., 2016).

Human literature supports the proposition that interstitial white matter, the equivalent of subplate in the postnatal human brain (Clancy et al., 2009; Suarez-Sola et al., 2009; Garcia-Marin et al., 2010; Hoerder-Suabedissen and Molnár, 2015), shows structural damage as a result of neonatal injury. Tissue from preterm human infants with periventricular leukomalacia showed a deficit in the number of MAP-2 expressing neurons throughout the interstitial white matter (Kinney et al., 2012). Scarring has been observed in interstitial white matter, alongside the expression of cell death markers, in brain tissue from human infants (Pogledic et al., 2014). The number of interstitial neurons present in white matter is known to peak at the developmental stage most sensitive to white matter injury (Kostovic et al., 2002; Kostovic and Judas, 2010). Immunohistochemical analysis on neonatal telencephalon samples obtained post-mortem from infants born at 25–32 weeks gestation showed a significant loss of GABA-ergic subplate neurons (Robinson et al., 2006). Many reviews have highlighted the interstitial white matter as a site which should be more thoroughly investigated in neonatal brain injury (Volpe et al., 1996; Leviton and Gressens, 2007; Volpe, 2009; Kostovic et al., 2011). Several of these reviews propose a role for subplate in resultant cognitive and behavioral deficits (Volpe, 2009, 1977; Kostovic et al., 2011), based on the established role of subplate in thalamocortical development. Unfortunately, post-mortem studies are unable to convey whether subplate pathology is responsible for any of the clinical outcomes in patients. The response of both human and rodent subplate to neonatal HI remains unclear, despite assertions of interest from the field. A systematic study of the subplate *in vitro* and *in vivo* following HI injury would provide valuable information. As subplate transiently integrates within the developing cortical circuitry (see **Figure 5**) interactions between subplate neurons and other cortical neurons, such as key interneuron populations, should also be further investigated (Marques-Smith et al., 2016).

There are several reasons for believing that subplate could be critical in neonatal HI. One is the proposed transient secretory function. Subplate is enriched in CSPGs, whereas the adjacent intermediate zone is not (Kostovic et al., 2014). Chondroitin sulfate proteoglycans (CSPGs) are generally secreted from cells, and the subplate transcriptome catalogs a plethora of genes involved in production of extracellular matrix and proteoglycans (Belgard et al., 2011; Hoerder-Suabedissen et al., 2013). Additional genes with subplate-restricted expression in the cortex encode secreted proteins, including neuroserpin (*Serpini1*), neuronal pentraxin 1 (*Nptx1*), and insulin-like growth factor binding protein 5 (*Igfbp5*) (Hoerder-Suabedissen et al., 2013), several of which have been validated by immunohistochemistry (Kondo et al., 2015). Connective tissue growth factor (CTGF), secreted extracellular matrix-associated protein involved in regulation of cellular adhesion, migration, mitogenesis, differentiation and survival (Brigstock, 1999; Stritt et al., 2009), is also detectable in the subplate at E18, increasing in intensity at P3 and P8 (Hoerder-Suabedissen et al., 2009, 2013). It is unknown whether subplate-secreted proteins serve a protective function in the developing brain or not. Related to this secretory function, subplate neurons express relatively mature rER (Kondo et al., 2015), an organelle essential for production of secreted proteins (reviewed in Novikoff, 1976; Pfeffer and Rothman, 1987; Lodish, 1988; Hurtley and Helenius, 1989; Pelham, 1989). As a result of the cellular pressures of high protein production, cells activate a series of mechanisms referred to as the ER stress response (Schroder and Kaufman, 2005; Wu and Kaufman, 2006; Kondo et al., 2011). Nissl staining demonstrates enriched protein production in subplate at P8 in mouse, although staining is much fainter in adult (Kondo et al., 2015). Morphology of subplate neurons is similar to that of rER-rich plasma cells under EM (Bloom, 1968; Kondo et al., 2015), and immunohistochemistry for ER stress marker binding immunoglobulin protein (BiP) confirmed that ER stress occurs in developing subplate (Okiyoneda et al., 2004; Kondo et al., 2005, 2012, 2015). BiP protein synthesis is up-regulated in whole brain under stress conditions, such as glucose deprivation, hypoxia, or the presence of toxins (Lee, 1987, 2001, 2005). It is not known exactly which proteins are secreted throughout development by the subplate, but this enhanced metabolic stress during hypoxia and the potential for neuroprotective secretion reinforce the value of further study of subplate in neonatal HI models and human patients.

## Neuroserpin: A Case for A Novel Neuroprotective Treatment

The development of novel treatments to supplement the sole currently licensed therapy, hypothermia (Jacobs et al., 2005, 2013; Shah et al., 2007; Tagin et al., 2012), is imperative. Since the discovery of this groundbreaking treatment, little progress has been made in identifying additive pharmacological therapies. Few potential treatments have translated to human clinical trials. Resuscitation at room temperature (Vento et al., 2001a,b; Rabi et al., 2007) and xenon gas administration alongside hypothermia (Hobbs et al., 2008; Thoresen et al., 2009) are the only therapies shown to have any additive effect. However, more recent publications have questioned the efficacy of xenon as an additive therapy alongside hypothermia. For example, randomized clinical trials have demonstrated that although xenon gas is a safe treatment, there is little or no therapeutic effect of combined hypothermia and xenon gas in moderate and severe cases of neonatal HI at 18 months follow-up (Azzopardi et al., 2013, 2015; Dingley et al., 2014). A similar experiment in rats found that xenon treatment made no difference to lesion size or neuronal cell numbers in cases of severe HI (Sabir et al., 2016). Barbiturate anticonvulsants have no effect on long-term neurological development when given following neonatal HI (reviewed in Goldberg et al., 1986; Hall et al., 1998; Singh et al., 2004; Vargas-Origel et al., 2004; Evans et al., 2007). Recent clinical studies suggest that high dose erythropoietin (EPo) treatment in term HI neonates reduces disability (Strunk et al., 2008; Zhu et al., 2009; McPherson and Juul, 2010). However, even proponents of this potential treatment advise caution in interpreting these early results, and the therapeutic effect by no means completely prevents disability. Combination therapy of *N*-acetylcysteine, a free radical scavenger, and systemic hypothermia reduces infarct volume after focal HI injury (Jatana et al., 2006). Another free radical scavenger, allopurinol, reduces cerebral edema and neuropathological damage (Palmer et al., 1990). However, these treatments have only been trialed in animals, and the field is still awaiting a candidate neuroprotectice molecule which is both safe and effective in neonatal humans (Lai and Yang, 2011; Pazos et al., 2012).

Another therapeutic approach under development for neonatal HI is stem cell therapy (reviewed in Parolini et al., 2010; Zhang X. et al., 2014; Douglas-Escobar and Weiss, 2015; González-Portillo et al., 2015; Ruiz et al., 2017). These therapies make use of evidence that transplantation of human bone-marrow derived stem cells into the lesion can assist brain plasticity (Bonifacio et al., 2011; Tajiri et al., 2013). Results of this therapy have been promising in animal models (Jendelová et al., 2004; Daadi et al., 2009a,b), however, the technique awaits validation in a clinical setting. Additionally, the use of stem cell therapy for cerebral palsy in human patients has produced mixed results (Li et al., 2012; Liao et al., 2013; Sharma, 2014).

Therefore, new approaches are required to identify potential neuroprotective molecules as treatments for neonatal HI (Tuor et al., 1995; Ferriero, 2004; Fan et al., 2010). Drawing on our detailed yet incomplete knowledge of the physiology of neonatal HI, several factors must be satisfied in a new potential therapy. All potential treatments should be safe for vulnerable neonates and not interfere with essential developmental milestones. This challenges the NMDA-inhibition strategies (Meldrum, 1990; Johnston, 2001, 2005), as the glutamate system is essential for setting up normal synaptic plasticity within the developing brain (Hattori and Wasterlain, 1990; Ikonomidou et al., 1999; Johnston, 2009). Treatments should also be specific, to avoid extreme adverse effects in these vulnerable infants. The ideal treatment would target molecules common to the excitotoxicity, oxidative stress and inflammation pathways (Chaudhari et al., 2014; Fischer and Maier, 2015; Chamorro et al., 2016; Burd et al., 2016). Targeting these common mediators would allow a single therapy to be efficacious against multiple mediators of brain damage, instead of merely eliciting a reshuffle of pathways to favor a different method of cell death. Here follows an outline of one potential therapy under development which may satisfy these theoretical restraints as a model to incite debate concerning how future therapies are identified. Enter: neuroserpin.

### Neuroserpin’s Molecular Pedigree

Neuroserpin is a neuronally secreted serine-protease inhibitor enzyme with roles in cell death, neural plasticity, and microglial activation. Neuroserpin was first identified in the secreted product of cultured neuronal axons (Osterwalder et al., 1996). Protein expression is primarily localized to neurons (Osterwalder et al., 1996; Hastings et al., 1997; Lee et al., 2015b). The protein neuroserpin is encoded in mouse and human by the gene *Serpini1. Serpini1* mRNA expression is specific to the CNS, both in mouse (Krueger et al., 1997) and human (Teesalu et al., 2004). *In situ* hybridization studies have demonstrated expression of *Serpini1* throughout cortex, hippocampus, and olfactory bulbs, with scattered expression in cerebellum, pons, and thalamus from birth until adulthood (Krueger et al., 1997). High *Serpini1* expression has been identified in subplate relative to Layer VI of cortex in P8 mouse brain by microarray (Hoerder-Suabedissen et al., 2009), confirmed by immunohistochemistry (Kondo et al., 2015). Although *Serpini1* expression has been demonstrated in human brain tissue (Teesalu et al., 2004), detailed maps of expression are currently missing. This highly specific localisation suggests that *Serpini1*/neuroserpin modulatory treatments may not create dangerous side effects in other vulnerable organs such as the cardiovascular and pulmonary systems.

**FIGURE 6:**
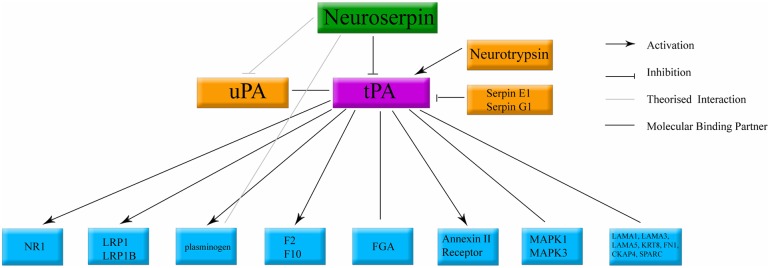
**Classical binding cascade for neuroserpin protein.** The main molecular target of neuroserpin is inhibition of tPA. Confirmed molecular binding partners are shown with a black line. Arrows denote confirmed activation. Orthogonal lines denote confirmed inactivation. Lines without caps denote confirmed binding with no documented physiological data. tPA, tissue plasminogen activator; uPA, urokinase plasminogen activator. All other abbreviations denote standard gene names.

Neuroserpin is an inhibitory enzyme. Its primary target is the adult human stroke treatment, tissue plasminogen activator (tPA) (Osterwalder et al., 1996, 1998; Barker-Carlson et al., 2002; Yepes and Lawrence, 2004a; Miranda and Lomas, 2006; Ricagno et al., 2009, 2010). Neuroserpin has also been shown to exhibit weak inhibition of plasmin and urokinase plasminogen activator (Osterwalder et al., 1998). This function is conserved across species (Schrimpf et al., 1997; Hill et al., 2001; Ellisdon et al., 2014). Through inhibition of tPA, neuroserpin has the potential to influence many distinct molecular cascades (Yepes and Lawrence, 2004b,c; Benarroch, 2007; Yepes, 2015). tPA, also secreted by neurons (Krystosek and Seeds, 1981a,b; Lochner et al., 2006), has been shown to interact with a large number of pathways; activating NMDA receptors (Qian et al., 1993; Nicole et al., 2001), activating microglia (Benchenane et al., 2004), and recruitment of cell death related cascades (Yepes and Lawrence, 2004a; Zhao et al., 2007), in addition to its function of cleaving vascular thrombosis (Rijken et al., 1979, 1980). The known components of the neuroserpin/tPA molecular cascade are summarized in **Figure 6**.

Other, tPA-independent enzymatic pathways for neuroserpin have also been described (Ma et al., 2012a). Cell lines over-expressing neuroserpin demonstrate an increase in *N*-cadherin protein expression and related cell adhesion, maintained when the tPA binding site of neuroserpin is mutated (Lee et al., 2008). *In vitro*, neuroserpin has been shown to prevent excitotoxic neuronal death induced by plasmin and kainic acid (Wu et al., 2010), and the 20 methionine residues present within neuroserpin have been claimed to convey protection against oxidative stress (Mohsenifar et al., 2007), and exogenous neuroserpin has been shown to possess anti-inflammatory properties (Munuswamy-Ramanujam et al., 2010). The molecular cascades behind these tPA independent functions are not well understood (Ma et al., 2012a). Therefore, neuroserpin has the pedigree to target multiple cell death pathways occurring in the brain during neonatal HI.

### Cell Death, Inflammation, and Plasticity

Neuroserpin’s physiological actions can be broadly categorized into three major pathways: cell death, neuronal plasticity, and immune cell activation. These closely follow the major classes of molecular change proposed by pathological theories of neonatal HI, suggesting that neuroserpin offers the potential to target multiple brain-damage pathways.

Neuroserpin has been implicated in protection against neuronal death (Galliciotti and Sonderegger, 2006). Exogenous neuroserpin administration *in vitro* decreases apoptosis caused by tPA, NMDA, kainic acid, and OGD (Lebeurrier et al., 2005a,b; Mohsenifar et al., 2007; Lee et al., 2008; Wu et al., 2010; Rodríguez-González et al., 2011a; Ma et al., 2012b). In addition, aberrant initiation of neuroserpin expression in cancer cells preserves the tumor and has been linked with increased treatment-resistance in prostate cancer (Chang et al., 2000; Hasumi et al., 2005; Valiente et al., 2014). Exogenous neuroserpin reduces spread of kainate-induced seizures in mouse, and decreases the expression of cell death markers (Yepes et al., 2002).

Of the three classes of neuroserpin function, its role in inflammation is the least well understood. When applied to vascular plaques *in vitro*, recombinant neuroserpin reduced T-Cell lymphocyte invasion (Munuswamy-Ramanujam et al., 2010). Administration of intracerebral neuroserpin directly following adult HI stroke in mouse, showed a qualitative decrease in the volume of infarct infiltrated by activated microglia (Yepes et al., 2000), and lower microglial inflammatory marker expression (Gelderblom et al., 2013). In human adult stroke patients, higher levels of neuroserpin in blood samples correlate with lower levels of immune marker proteins (Rodríguez-González et al., 2011c). There is considerable debate whether microglial activation is beneficial or detrimental in neonatal HI (McRae et al., 1995; Mallard et al., 2013; Kaur et al., 2013), so understanding the role of neuroserpin in balancing this cascade could provide important data. The lack of a complete molecular network explaining these anti-inflammatory effects highlights the complexity of the pathways involved in cell death and inflammation. Understanding the underlying explanation for these changes will be an important preliminary to administration in human infants who do not live in the highly sterile environments maintained for experimental animals.

In addition, neuroserpin is involved in neuronal plasticity. This could be problematic in the development of neuroserpin as a neonatal HI treatment for human patients, as the neonatal human brain circuitry is undergoing a great deal of developmental plasticity modifications (reviewed in Aoki and Siekevitz, 1988; Partanen et al., 2013; Simpson et al., 2014). The first evidence that neuroserpin may have a role in neuronal plasticity originated from studies of endocrine cell lines, which grow neurite-like processes when neuroserpin is administered to their media (Hill et al., 2001; Jacovina et al., 2001; Borges et al., 2010). At the molecular level, neuroserpin secretion from dense core vesicles is enhanced by depolarisation in cultured neurons (Berger et al., 1999; Ishigami et al., 2007). Monocular enucleation at P11 in mouse led to decreased *Serpini1* mRNA expression in contralateral primary visual cortex compared to the control hemisphere (Wannier-Morino et al., 2003). The relative importance of this molecular cascade for human plasticity is as yet unclear.

### Neuroserpin in *In Vitro* and Adult Hypoxia Ischaemia

Neuroserpin has been studied extensively in cell culture models of HI stroke. Administration of neuroserpin protects mouse cortical neurons *in vitro* against NMDA-induced excitotoxicity, but not AMPA-induced excitotoxicity (Lebeurrier et al., 2005a,b; Ma et al., 2012b). Further, neuroserpin is protective in an *in vitro* model of HI, OGD (Wu et al., 2010; Rodríguez-González et al., 2011a; Ma et al., 2012b). Both neurons and astrocytes cultured in OGD undergo less apoptosis and do not exhibit damaged neural processes when exogenous neuroserpin is administered following OGD (Rodríguez-González et al., 2011a; Ma et al., 2012b). Hippocampal neurons from both wild-type and tPA knockout mice were protected from OGD, plasmin-mediated cell death and kainic acid by neuroserpin administration (Wu et al., 2010), demonstrating that neuroserpin’s neuroprotective role is not exclusively dependent on tPA inhibition.

Neuroserpin is also neuroprotective *in vivo*. The major model used to simulate hypoxic-ischaemic stroke in adult rodents is analogous to the Rice–Vannucci model described above for neonates (Eklöf and Siesjö, 1973; Rice et al., 1981; Bederson et al., 1986). Adult rats injected with neuroserpin before left common carotid artery ligation followed by acute hypoxia showed a decrease in infarct volume compared to controls, in addition to a decrease in expression of apoptosis markers (Yepes et al., 2000). Neuroserpin knockout mice show increased lesion volumes following adult HI (Cinelli et al., 2001; Gelderblom et al., 2013), whereas transgenic mice expressing six-times normal neuroserpin expression demonstrate smaller infarct volumes (Cinelli et al., 2001). Exogenous neuroserpin injection is also protective against NMDA injection in live adult rat (Lebeurrier et al., 2005a). Currently, the only evidence for this has come from adults models of HI stroke, far from conclusive evidence of neonatal protection due to the differences in neuroanatomy and neurochemistry between the developing and adult brain. However, this work remains of utility, as at the very least investigating the neuroserpin-tPA system in neonatal models will add to understanding of the differences between neonatal and adult response to HI.

Neuroserpin administration in rats increases the window for effective tPA treatment in a model of adult stroke (Zhang et al., 2002). Increased tPA expression is found in neuroserpin knockout mice undergoing adult HI injury compared to controls (Cinelli et al., 2001; Gelderblom et al., 2013). However, exogenous neuroserpin is neuroprotective in tPA knockout mice (Wu et al., 2010). Ischaemic-reperfusion-induced injury in the retina of tPA knockout mice demonstrated rescued retinal apoptotic marker expression when injected with neuroserpin (Gu et al., 2015). Therefore, the neuroprotective properties of neuroserpin in adult HI appear to involve both neuroserpin’s tPA-dependent and tPA-independent molecular cascades.

A role for neuroserpin in human adult HI stroke has also been described. Correlative studies have shown that high concentrations of neuroserpin in blood samples from human patients are associated with better functional outcome (Rodríguez-González et al., 2011b) and reduced inflammation (Rodríguez-González et al., 2011c). However, attempts to find a relationship between neuroserpin polymorphisms in humans and likelihood of stroke have produced little evidence of protective neuroserpin variants (Cole et al., 2007; Tjärnlund-Wolf et al., 2011). Mutations in the neuroserpin gene have been associated with two rare hereditary disorders (Galliciotti and Sonderegger, 2006); individuals present either with epilepsy (Yepes et al., 2002; Coutelier et al., 2008) or dementia with neuroserpin inclusion bodies (Davis et al., 1999a,b, 2002; Yazaki et al., 2001; Ricagno et al., 2010). It is not currently known whether these disorders are caused by novel functions of the mutated protein or by loss of physiological functions (Lee et al., 2015a).

### Neuroserpin in Neonatal Hypoxia-Ischaemia

The evidence above from adult models of HI stroke is far from conclusive evidence of neonatal protection due to differences between the developing and adult brain. However, this work remains of utility, as at the very least investigating neuroserpin in neonatal models will add to understanding of the differences between neonatal and adult response to HI.

Evidence for a role of neuroserpin in neonatal HI is not entirely absent. A patent application published online claims that neuroserpin injection 4 h after neonatal HI in rat pups reduced infarct volume, but had no effect on LPS-sensitized HI (Kuan et al., 2010). However, the small number of published rodent microarrays following neonatal HI have failed to detect significant changes in *Serpini1* expression (Carmel et al., 2004; Hedtjarn et al., 2004a,b; Juul et al., 2009; Nagel et al., 2012; Rognlien et al., 2014). This could be explained by the delay between HI injury and sample collection, 6 h to 7 days post injury in this sample, which may be too early or too late to expect to see genomic changes in *Serpini1* expression. Also, microarray studies cannot reveal whether neonatal HI causes differences in neuroserpin translation or secretion, neither of which have yet been studied in neonatal HI.

Despite the lack of evidence for neuroserpin’s direct role in neonatal HI, substantially more has been published documenting effects of its primary molecular target, tPA, in neonatal HI. tPA is broadly considered to be deleterious to cerebral recovery from neonatal HI (Adhami et al., 2008; Omouendze et al., 2013). In P7 rats which underwent unilateral common carotid artery ligation, tPA treatment was found to impair blood flow throughout the brain, an effect which was rescued by injection of the tPA inhibitor α2-antiplasmin (Adhami et al., 2008). Release of tPA by the cerebral microvasculature, as seen following neonatal HI, has also been shown to compromise the glucose content of the extracellular medium in neonatal mouse cortical culture (Henry et al., 2013). Administration of tPA inhibitor Plasminogen-activator-inhibitor 1 (PAI1) reduces locomotor disorder and white matter damage in LPS-sensitized neonatal HI rats (Yang et al., 2013a,b; Yang and Kuan, 2015). This pathway is relatively understudied in neonatal HI research, despite the potential to target a multitude of injury-relevant molecules. Drawing attention to molecules like neuroserpin and tPA, potential common moderators of injury pathways, will be essential to the development of new efficacious pharmacological treatments.

## Conclusion

Neonatal HI remains the most common cause of infant death and disability globally. Reducing the burden of morbidity should be a high priority for biomedical research. A dearth of treatments are currently available postnatally for these vulnerable infants, restricted to only hypothermia, a breakthrough treatment which is not fully effective in all cases, with no currently licensed additive pharmacological treatments. A complex network of interacting molecular cascades, including excitotoxicity, oxidative stress, and inflammation contribute to the gradually developing pattern of neuronal cell death seen in asphyxiated neonates. Targeting common mediators of these pathways with specific targets linked to far-reaching effects offers a potential approach to generating new pharmacological treatments to exact neuroprotection in these neonatal patients.

## Author Contributions

All authors listed have made substantial, direct, and intellectual contribution to this work, and approved its final version for publication. LM, LS, AH-S, and ZM: conceived the review focus, conducted literature review, evaluated the literature. LM: reviewed literature, wrote first draft, critically revised the first draft, and finalized the manuscript. LS, AH-S, and ZM: gave feedback, and finalized the manuscript. All authors approved final version of manuscript.

## Conflict of Interest Statement

The authors declare that the research was conducted in the absence of any commercial or financial relationships that could be construed as a potential conflict of interest.

## Abbreviations

AMPA: alpha-amino-3-hydroxy-5-methyl-4-isoxazole propionic acid
BBB: blood brain barrier
CPR: cardiopulmonary resuscitation
CNS: central nervous system
CSF: cortico-spinal fluid
CSPG: chondroitin sulfate proteoglycan
CTGF: connective tissue growth factor
E(18): embryonic day (18)
EM: electron microscopy
GABA: γ-aminobutyric acid
HI: hypoxia-ischaemia
HIF-1: hypoxia inducible factor 1
IL-1ß: interleukin-1ß
IL-6: interleukin-6
IL-9: interleukin-9
LPS: lipopolysaccharide
MRI: magnetic resonance imaging
MRS: magnetic resonance spectroscopy
NMDA: *N*-methyl-D-aspartate
OGD: oxygen glucose deprivation
P(8): postnatal day (8)
poly(I:C): polyinosinic-polycytidylic acid
rER: rough endoplasmic reticulum
TNF-α: tumour-necrosis factor-α
tPA: tissue plasminogen activator
VEGF: vascular endothelial growth factor

## References

[B1] Adamsons K. Myers R. E. (1973). Perinatal asphyxia, causes, detection and neurologic sequelae. *Pediatr. Clin. North Am.* 20 465–480. 10.1016/S0031-3955(16)32855-3 4633766

[B2] Ådén U. Halldner L. Lagercrantz H. Dalmau I. Ledent C. Fredholm B. B. (2003). Aggravated brain damage after hypoxic ischemia in immature adenosine A2A knockout mice. *Stroke* 34 739–744. 10.1161/01.STR.0000060204.67672.8B 12624301

[B3] Adhami F. Yu D. Yin W. Schloemer A. Burns K. A. Liao G. (2008). Deleterious effects of plasminogen activators in neonatal cerebral hypoxia-ischemia. *Am. J. Pathol.* 172 1704–1716. 10.2353/ajpath.2008.070979 18467699 PMC2408429

[B4] Agrawal V. Hirsch E. (2012). Intrauterine infection and preterm labor. *Semin. Fetal Neonatal Med.* 17 12–19. 10.1016/j.siny.2011.09.001 21944863 PMC3242863

[B5] al Naqeeb N. Edwards A. D. Cowan F. M. Azzopardi D. (1999). Assessment of neonatal encephalopathy by amplitude-integrated electroencephalography. *Pediatrics* 103 1263–1271. 10.1542/peds.103.6.1263 10353940

[B6] Albrecht J. Hanganu I. L. Heck N. Luhmann H. J. (2005). Oxygen and glucose deprivation induces major dysfunction in the somatosensory cortex of the newborn rat. *Eur. J. Neurosci.* 22 2295–2305. 10.1111/j.1460-9568.2005.04398.x 16262667

[B7] Alexander M. Garbus H. Smith A. Rosenkrantz T. Fitch R. (2014). Behavioral and histological outcomes following neonatal HI injury in a preterm (P3) and term (P7) rodent model. *Behav. Brain Res.* 259 85–96. 10.1016/j.bbr.2013.10.038 24185032 PMC3906857

[B8] Al-Ghoul W. M. Miller M. W. (1989). Transient expression of Alz-50 immunoreactivity in developing rat neocortex: a marker for naturally occurring neuronal death? *Brain Res.* 481 361–367. 10.1016/0006-8993(89)90815-9 2720388

[B9] Allendoerfer K. L. Shatz C. J. (1994). The subplate, a transient neocortical structure: its role in the development of connections between thalamus and cortex. *Annu. Rev. Neurosci.* 17 185–218. 10.1146/annurev.ne.17.030194.001153 8210173

[B10] Alsharnoubi J. M. Odland H. H. Saugstad O. D. (2012). Nicotine does not influence NF-kappaB activity in neonatal mice reoxygenated with room-air or 100% oxygen. *J. Mater. Fetal Neonatal Med.* 25 2102–2105. 10.3109/14767058.2012.678434 22489644

[B11] Alvarez-Diaz A. Hilario E. de Cerio F. G. Valls-i-Soler A. Alvarez-Diaz F. J. (2007). Hypoxic-ischemic injury in the immature brain–key vascular and cellular players. *Neonatology* 92 227–235. 10.1159/000103741 17556841

[B12] Andine P. Thordstein M. Kjellmer I. Nordborg C. Thiringer K. Wennberg E. (1990). Evaluation of brain damage in a rat model of neonatal hypoxic-ischemia. *J. Neurosci. Methods* 35 253–260. 10.1016/0165-0270(90)90131-X 2084395

[B13] Anju T. R. Abraham P. M. Antony S. Paulose C. S. (2010a). Alterations in cortical GABAB receptors in neonatal rats exposed to hypoxic stress: role of glucose, oxygen, and epinephrine resuscitation. *Mol. Cell. Biochem.* 343 1–11. 10.1007/s11010-010-0491-9 20473556

[B14] Anju T. R. Ajayan M. S. Paulose C. S. (2013a). Disruption of cerebellar cholinergic system in hypoxic neonatal rats and its regulation with glucose, oxygen and epinephrine resuscitations. *Neuroscience* 236 253–261. 10.1016/j.neuroscience.2012.12.056 23376739

[B15] Anju T. R. Binoy J. Anitha M. Paulose C. S. (2012a). Striatal GABA receptor alterations in hypoxic neonatal rats: role of glucose, oxygen and epinephrine treatment. *Neurochem. Res.* 37 629–638. 10.1007/s11064-011-0654-4 22089934

[B16] Anju T. R. Jayanarayanan S. Paulose C. S. (2011a). Decreased GABAB receptor function in the cerebellum and brain stem of hypoxic neonatal rats: role of glucose, oxygen and epinephrine resuscitation. *J. Biomed. Sci.* 18:31 10.1186/1423-0127-18-31 PMC3114712 21569387

[B17] Anju T. R. Korah P. K. Jayanarayanan S. Paulose C. S. (2011b). Enhanced brain stem 5HT(2)A receptor function under neonatal hypoxic insult: role of glucose, oxygen, and epinephrine resuscitation. *Mol. Cell. Biochem.* 354 151–160. 10.1007/s11010-011-0814-5 21484469

[B18] Anju T. R. Mathew J. Jayanarayanan S. Paulose C. S. (2010b). Cerebellar 5HT2A receptor function under hypoxia in neonatal rats: role of glucose, oxygen, and epinephrine resuscitation. *Respir. Physiol. Neurobiol.* 172 147–153. 10.1016/j.resp.2010.05.009 20471502

[B19] Anju T. R. Naijil G. Shilpa J. Roshni T. Paulose C. S. (2013b). Neonatal hypoxic insult-mediated cholinergic disturbances in the brain stem: effect of glucose, oxygen and epinephrine resuscitation. *Neurol. Sci.* 34 287–296. 10.1007/s10072-012-0989-x 22395945

[B20] Anju T. R. Nandhu M. S. Jes P. Paulose C. S. (2011c). Endocrine regulation of neonatal hypoxia: role of glucose, oxygen, and epinephrine supplementation. *Fetal Pediatr. Pathol.* 30 338–349. 10.3109/15513815.2011.587498 21846315

[B21] Anju T. R. Peeyush Kumar T. Paulose C. S. (2010c). Decreased GABAA receptors functional regulation in the cerebral cortex and brainstem of hypoxic neonatal rats: effect of glucose and oxygen supplementation. *Cell Mol. Neurobiol.* 30 599–606. 10.1007/s10571-009-9485-0 20033840 PMC11498792

[B22] Anju T. R. Smijin S. Chinthu R. Paulose C. S. (2012b). Decreased cholinergic function in the cerebral cortex of hypoxic neonatal rats: role of glucose, oxygen and epinephrine resuscitation. *Respir. Physiol. Neurobiol.* 180 8–13. 10.1016/j.resp.2011.08.013 21907834

[B23] Anju T. R. Smijin S. Korah P. K. Paulose C. S. (2011d). Cortical 5HT 2A receptor function under hypoxia in neonatal rats: role of glucose, oxygen, and epinephrine resuscitation. *J. Mol. Neurosci.* 43 350–357. 10.1007/s12031-010-9449-3 20857344

[B24] Anju T. R. Paulose C. S. (2011). Amelioration of hypoxia-induced striatal 5-HT(2A) receptor, 5-HT transporter and HIF1 alterations by glucose, oxygen and epinephrine in neonatal rats. *Neurosci. Lett.* 502 129–132. 10.1016/j.neulet.2011.05.236 21683764

[B25] Anju T. R. Paulose C. S. (2013). Striatal cholinergic functional alterations in hypoxic neonatal rats: role of glucose, oxygen, and epinephrine resuscitation. *Biochem. Cell Biol.* 91 350–356. 10.1139/bcb-2012-0102 24032686

[B26] Aoki C. Siekevitz P. (1988). Plasticity in brain development. *Sci. Am.* 259 56–64. 10.1038/scientificamerican1288-56 2849807 PMC2841150

[B27] Applegate C. D. Jensen F. Burchfiel J. L. Lombroso C. (1996). The effects of neonatal hypoxia on kindled seizure development and electroconvulsive shock profiles. *Epilepsia* 37 723–727. 10.1111/j.1528-1157.1996.tb00642.x 8764809

[B28] Arteni N. S. Salgueiro J. Torres I. Achaval M. Netto C. A. (2003). Neonatal cerebral hypoxia–ischemia causes lateralized memory impairments in the adult rat. *Brain Res.* 973 171–178. 10.1016/S0006-8993(03)02436-3 12738060

[B29] Aso K. Scher M. S. Barmada M. A. (1989). Neonatal electroencephalography and neuropathology. *J. Clin. Neurophysiol.* 6 103–123. 10.1097/00004691-198904000-00001 2708514

[B30] Aujla P. K. Fetell M. R. Jensen F. E. (2009). Talampanel suppresses the acute and chronic effects of seizures in a rodent neonatal seizure model. *Epilepsia* 50 694–701. 10.1111/j.1528-1167.2008.01947.x 19220413 PMC2672962

[B31] Autheman D. Sheldon R. A. Chaudhuri N. von Arx S. Siegenthaler C. Ferriero D. M. (2012). Glutathione peroxidase overexpression causes aberrant ERK activation in neonatal mouse cortex after hypoxic preconditioning. *Pediatr. Res.* 72 568–575. 10.1038/pr.2012.124 23007029 PMC3529181

[B32] Azzopardi D. Robertson N. J. Bainbridge A. Cady E. Charles-Edwards G. Deierl A. (2015). Moderate hypothermia within 6 h of birth plus inhaled xenon versus moderate hypothermia alone after birth asphyxia (TOBY-Xe): a proof-of-concept, open-label, randomised controlled trial. *Lancet Neurol.* 10.1016/S1474-4422(15)00347-6 [Epub ahead of print]. PMC4710577 26708675

[B33] Azzopardi D. Robertson N. J. Kapetanakis A. Griffiths J. Rennie J. M. Mathieson S. R. (2013). Anticonvulsant effect of xenon on neonatal asphyxial seizures. *Arch. Dis. Child. Fetal Neonatal Ed.* 98 F437–F439. 10.1136/archdischild-2013-303786 23572341

[B34] Azzopardi D. V. Strohm B. Edwards A. D. Dyet L. Halliday H. L. Juszczak E. (2009). Moderate hypothermia to treat perinatal asphyxial encephalopathy. *N. Engl. J. Med.* 361 1349–1358. 10.1056/NEJMoa0900854 19797281

[B35] Baburamani A. A. Ek C. J. Walker D. W. Castillo-Melendez M. (2012). Vulnerability of the developing brain to hypoxic-ischemic damage: contribution of the cerebral vasculature to injury and repair? *Front. Physiol.* 3:424 10.3389/fphys.2012.00424 PMC3493883 23162470

[B36] Back S. A. Gan X. Li Y. Rosenberg P. A. Volpe J. J. (1998). Maturation-dependent vulnerability of oligodendrocytes to oxidative stress-induced death caused by glutathione depletion. *J. Neurosci.* 18 6241–6253. 9698317 10.1523/JNEUROSCI.18-16-06241.1998 PMC6793198

[B37] Balduini W. Carloni S. Buonocore G. (2012). Autophagy in hypoxia-ischemia induced brain injury. *J. Matern. Fetal Neonatal Med.* 25(Suppl. 1), 30–34. 10.3109/14767058.2012.663176 22385271

[B38] Balduini W. De Angelis V. Mazzoni E. Cimino M. (2000). Long-lasting behavioral alterations following a hypoxic/ischemic brain injury in neonatal rats. *Brain Res.* 859 318–325. 10.1016/S0006-8993(00)01997-1 10719080

[B39] Balduini W. De Angelis V. Mazzoni E. Cimino M. (2001). Simvastatin protects against long-lasting behavioral and morphological consequences of neonatal hypoxic/ischemic brain injury. *Stroke* 32 2185–2191. 10.1161/hs0901.094287 11546915

[B40] Barker-Carlson K. Lawrence D. A. Schwartz B. S. (2002). Acyl-enzyme complexes between tissue-type plasminogen activator and neuroserpin are short-lived in vitro. *J. Biol. Chem.* 277 46852–46857. 10.1074/jbc.M207740200 12228252

[B41] Barkovich A. J. Miller S. P. Bartha A. Newton N. Hamrick S. E. Mukherjee P. (2006). MR imaging, MR spectroscopy, and diffusion tensor imaging of sequential studies in neonates with encephalopathy. *AJNR Am. J. Neuroradiolo.* 27 533–547. PMC7976955 16551990

[B42] Barkovich A. J. Westmark K. Partridge C. Sola A. Ferriero D. M. (1995). Perinatal asphyxia: MR findings in the first 10 days. *AJNR Am. J. Neuroradiol.* 16 427–438. 7793360 PMC8337672

[B43] Barth T. M. Stanfield B. B. (1990). The recovery of forelimb-placing behavior in rats with neonatal unilateral cortical damage involves the remaining hemisphere. *J. Neurosci.* 10 3449–3459. 2213147 10.1523/JNEUROSCI.10-10-03449.1990 PMC6570173

[B44] Baud O. Daire J. L. Dalmaz Y. Fontaine R. H. Krueger R. C. Sebag G. (2004). Gestational hypoxia induces white matter damage in neonatal rats: a new model of periventricular leukomalacia. *Brain Pathol.* 14 1–10. 10.1111/j.1750-3639.2004.tb00492.x 14997932 PMC8095946

[B45] Bauer H. C. Bauer H. Lametschwandtner A. Amberger A. Ruiz P. Steiner M. (1993). Neovascularization and the appearance of morphological characteristics of the blood-brain barrier in the embryonic mouse central nervous system. *Brain Res. Dev. Brain Res.* 75 269–278. 10.1016/0165-3806(93)90031-5 8261616

[B46] Bederson J. B. Pitts L. H. Tsuji M. Nishimura M. C. Davis R. L. Bartkowski H. (1986). Rat middle cerebral artery occlusion: evaluation of the model and development of a neurologic examination. *Stroke* 17 472–476. 10.1161/01.STR.17.3.472 3715945

[B47] Belgard T. G. Marques A. C. Oliver P. L. Abaan H. O. Sirey T. M. Hoerder-Suabedissen A. (2011). A transcriptomic atlas of mouse neocortical layers. *Neuron* 71 605–616. 10.1016/j.neuron.2011.06.039 21867878 PMC3163272

[B48] Bell M. J. Hallenbeck J. M. (2002). Effects of intrauterine inflammation on developing rat brain. *J. Neurosci. Res.* 70 570–579. 10.1002/jnr.10423 12404511

[B49] Benarroch E. E. (2007). Tissue plasminogen activator: beyond thrombolysis. *Neurology* 69 799–802. 10.1212/01.wnl.0000269668.08747.78 17709713

[B50] Benchenane K. López-Atalaya J. P. Fernández-Monreal M. Touzani O. Vivien D. (2004). Equivocal roles of tissue-type plasminogen activator in stroke-induced injury. *Trends Neurosci.* 27 155–160. 10.1016/j.tins.2003.12.011 15036881

[B51] Bennet L. Peebles D. M. Edwards A. D. Rios A. Hanson M. A. (1998). The cerebral hemodynamic response to asphyxia and hypoxia in the near-term fetal sheep as measured by near infrared spectroscopy. *Pediatr. Res.* 44 951–957. 10.1203/00006450-199812000-00022 9853934

[B52] Berger P. Kozlov S. V. Cinelli P. Krüger S. R. Vogt L. Sonderegger P. (1999). Neuronal depolarization enhances the transcription of the neuronal serine protease inhibitor neuroserpin. *Mol. Cell. Neurosci.* 14 455–467. 10.1006/mcne.1999.0804 10656253

[B53] Bergeron J. D. Deslauriers J. Grignon S. Fortier L. C. Lepage M. Stroh T. (2013). White matter injury and autistic-like behavior predominantly affecting male rat offspring exposed to group B streptococcal maternal inflammation. *Dev. Neurosci.* 35 504–515. 10.1159/000355656 24246964

[B54] Bernard S. A. Gray T. W. Buist M. D. Jones B. M. Silvester W. Gutteridge G. (2002). Treatment of comatose survivors of out-of-hospital cardiac arrest with induced hypothermia. *N. Engl. J. Med.* 346 557–563. 10.1056/NEJMoa003289 11856794

[B55] Biagioni E. Mercuri E. Rutherford M. Cowan F. Azzopardi D. Frisone M. F. (2001). Combined use of electroencephalogram and magnetic resonance imaging in full-term neonates with acute encephalopathy. *Pediatrics* 107 461–468. 10.1542/peds.107.3.461 11230583

[B56] Billiards S. S. Haynes R. L. Folkerth R. D. Borenstein N. S. Trachtenberg F. L. Rowitch D. H. (2008). Myelin abnormalities without oligodendrocyte loss in periventricular leukomalacia. *Brain Pathol.* 18 153–163. 10.1111/j.1750-3639.2007.00107.x 18177464 PMC2770329

[B57] Blaise S. A. Nedelec E. Alberto J. M. Schroeder H. Audonnet S. Bossenmeyer-Pourie C. (2009). Short hypoxia could attenuate the adverse effects of hyperhomocysteinemia on the developing rat brain by inducing neurogenesis. *Exp. Neurol.* 216 231–238. 10.1016/j.expneurol.2008.11.020 19124018

[B58] Blomgren K. Hagberg H. (2006). Free radicals, mitochondria, and hypoxia-ischemia in the developing brain. *Free Radic. Biol. Med.* 40 388–397. 10.1016/j.freeradbiomed.2005.08.040 16443153

[B59] Bloom W. (1968). *DW Fawcett A Textbook of Histology.* Amsterdam: Saunders, 582.

[B60] Boksa P. Zhang Y. Nouel D. (2015). Maternal oxytocin administration before birth influences the effects of birth anoxia on the neonatal rat brain. *Neurochem. Res.* 40 1631–1643. 10.1007/s11064-015-1645-7 26108713

[B61] Bona E. Hagberg H. Løberg E. M. Bågenholm R. Thoresen M. (1998). Protective effects of moderate hypothermia after neonatal hypoxia-ischemia: short-and long-term outcome. *Pediatr. Res.* 43 738–745. 10.1203/00006450-199806000-00005 9621982

[B62] Bonifacio S. L. Glass H. C. Peloquin S. Ferriero D. M. (2011). A new neurological focus in neonatal intensive care. *Nat. Rev. Neurol.* 7 485–494. 10.1038/nrneurol.2011.119 21808297

[B63] Borges V. M. Lee T. W. Christie D. L. Birch N. P. (2010). Neuroserpin regulates the density of dendritic protrusions and dendritic spine shape in cultured hippocampal neurons. *J. Neurosci. Res.* 88 2610–2617. 10.1002/jnr.22428 20648651

[B64] Boss V. Sola A. Wen T. C. Decker M. J. (2005). Mild intermittent hypoxia does not induce stress responses in the neonatal rat brain. *Biol. Neonate* 88 313–320. 10.1159/000087629 16113526

[B65] Boylan G. B. Young K. Panerai R. B. Rennie J. M. Evans D. H. (2000). Dynamic cerebral autoregulation in sick newborn infants. *Pediatr. Res.* 48 12–17. 10.1203/00006450-200007000-00005 10879794

[B66] Brackmann F. A. Link A. S. Jung S. Richter M. Zoglauer D. Walkinshaw G. (2013). Activin A regulation under global hypoxia in developing mouse brain. *Brain Res.* 1531 65–74. 10.1016/j.brainres.2013.07.039 23916668

[B67] Brehmer F. Bendix I. Prager S. van de Looij Y. Reinboth B. S. Zimmermanns J. (2012). Interaction of inflammation and hyperoxia in a rat model of neonatal white matter damage. *PLoS ONE* 7:e49023 10.1371/journal.pone.0049023 PMC3498343 23155446

[B68] Brigstock D. R. (1999). The Connective Tissue Growth Factor/Cysteine-Rich 61/Nephroblastoma Overexpressed (CCN) Family 1. *Endocr. Rev.* 20 189–206. 10.1210/er.20.2.189 10204117

[B69] Brochu M. E. Girard S. Lavoie K. Sebire G. (2011). Developmental regulation of the neuroinflammatory responses to LPS and/or hypoxia-ischemia between preterm and term neonates: an experimental study. *J. Neuroinflamm.* 20 55 10.1186/1742-2094-8-55 PMC3121616 21599903

[B70] Brongholi K. Souza D. G. Bainy A. C. Dafre A. L. Tasca C. I. (2006). Oxygen-glucose deprivation decreases glutathione levels and glutamate uptake in rat hippocampal slices. *Brain Res.* 1083 211–218. 10.1016/j.brainres.2006.02.003 16530736

[B71] Brooks K. J. Clark J. B. Bates T. E. (1996). Assessment of energy metabolism in the developing brain following aglycemic hypoxia by1H and31P NMR. *Neurochem. Res.* 21 1089–1095. 10.1007/BF02532419 8897472

[B72] Brunjes P. C. (1985). A stereological study of neocortical maturation in the precocial mouse, *Acomys cahirinus*. *Brain Res.* 351 279–287. 10.1016/0165-3806(85)90199-3 3995352

[B73] Brunjes P. C. (1989). A comparative study of prenatal development in the olfactory bulb, neocortex and hippocampal region of the precocial mouse *Acomys cahirinus* and rat. *Brain Res. Dev. Brain Res.* 49 7–25. 10.1016/0165-3806(89)90055-2 2791267

[B74] Brunjes P. C. (1990). The precocial mouse, *Acomys cahirinus*. *Psychobiology* 18 339–350.

[B75] Brunjes P. C. Korol D. L. Stern K. G. (1989). Prenatal neurogenesis in the telencephalon of the precocial mouse *Acomys cahirinus*. *Neurosci. Lett.* 107 114–119. 10.1016/0304-3940(89)90801-x 2616023

[B76] Budday S. Raybaud C. Kuhl E. (2014). A mechanical model predicts morphological abnormalities in the developing human brain. *Sci. Rep.* 4:5644 10.1038/srep05644 PMC4090617 25008163

[B77] Burd I. Welling J. Kannan G. Johnston M. V. (2016). Chapter five-excitotoxicity as a common mechanism for fetal neuronal injury with hypoxia and intrauterine inflammation. *Adv. Pharmacol.* 76 85–101. 10.1016/bs.apha.2016.02.003 27288075

[B78] Bustamante D. Morales P. Pereyra J. T. Goiny M. Herrera-Marschitz M. (2007). Nicotinamide prevents the effect of perinatal asphyxia on dopamine release evaluated with in vivo microdialysis 3 months after birth. *Exp. Brain Res.* 177 358–369. 10.1007/s00221-006-0679-0 17051386

[B79] Buwalda B. Nyakas C. Vosselman H. J. Luiten P. G. (1995). Effects of early postnatal anoxia on adult learning and emotion in rats. *Behav. Brain Res.* 67 85–90. 10.1016/0166-4328(94)00108-R 7748505

[B80] Cai J. Kang Z. Liu K. Liu W. Li R. Zhang J. H. (2009). Neuroprotective effects of hydrogen saline in neonatal hypoxia–ischemia rat model. *Brain Res.* 1256 129–137. 10.1016/j.brainres.2008.11.048 19063869

[B81] Cai Z. Pang Y. Xiao F. Rhodes P. G. (2001). Chronic ischemia preferentially causes white matter injury in the neonatal rat brain. *Brain Res.* 898 126–135. 10.1016/S0006-8993(01)02180-1 11292456

[B82] Caputa M. Rogalska J. Wentowska K. Nowakowska A. (2005). Perinatal asphyxia, hyperthermia and hyperferremia as factors inducing behavioural disturbances in adulthood: a rat model. *Behav. Brain Res.* 163 246–256. 10.1016/j.bbr.2005.05.015 16038989

[B83] Carloni S. Buonocore G. Balduini W. (2008). Protective role of autophagy in neonatal hypoxia-ischemia induced brain injury. *Neurobiol. Dis.* 32 329–339. 10.1016/j.nbd.2008.07.022 18760364

[B84] Carloni S. Carnevali A. Cimino M. Balduini W. (2007). Extended role of necrotic cell death after hypoxia-ischemia-induced neurodegeneration in the neonatal rat. *Neurobiol. Dis.* 27 354–361. 10.1016/j.nbd.2007.06.009 17681771

[B85] Carlos R. Seidler F. J. Lappi S. Slotkin T. A. (1991a). Fetal dexamethasone exposure affects basal ornithine decarboxylase activity in developing rat brain regions and alters acute responses to hypoxia and maternal separation. *Neonatology* 59 69–77. 10.1159/000243325 2036470

[B86] Carlos R. Seidler F. J. Slotkin T. A. (1991b). Fetal dexamethasone exposure sensitizes neonatal rat brain to hypoxia: effects on protein and DNA synthesis. *Dev. Brain Res.* 64 161–166. 1786639 10.1016/0165-3806(91)90220-d

[B87] Carmel J. B. Kakinohana O. Mestril R. Young W. Marsala M. Hart R. P. (2004). Mediators of ischemic preconditioning identified by microarray analysis of rat spinal cord. *Exp. Neurol.* 185 81–96. 10.1016/j.expneurol.2003.09.007 14697320

[B88] Carmeliet P. Storkebaum E. (2002). Vascular and neuronal effects of VEGF in the nervous system: implications for neurological disorders. *Semin. Cell Dev. Biol.* 13 39–53. 10.1006/scdb.2001.0290 11969370

[B89] Casolini P. Zuena A. R. Cinque C. Matteucci P. Alema G. S. Adriani W. (2005). Sub-neurotoxic neonatal anoxia induces subtle behavioural changes and specific abnormalities in brain group-I metabotropic glutamate receptors in rats. *J. Neurochem.* 95 137–145. 10.1111/j.1471-4159.2005.03349.x 16181418

[B90] Chamorro A. Dirnagl U. Urra X. Planas A. M. (2016). Neuroprotection in acute stroke: targeting excitotoxicity, oxidative and nitrosative stress, and inflammation. *Lancet Neurol.* 15 869–881. 10.1016/S1474-4422(16)00114-9 27180033

[B91] Chang W. W. Chang N. Lin S. Wu C. Wu F. Y. (2000). Tissue-specific cancer-related serpin gene cluster at human chromosome band 3q26. *Genes Chromosomes Cancer* 29 240–255. 10.1002/1098-2264(2000)9999:9999<::AID-GCC1029>3.0.CO;2-A 10992299

[B92] Chau V. Clément J. Robitaille Y. D’Anjou G. Vanasse M. (2008). Congenital axonal neuropathy and encephalopathy. *Pediatr. Neurol.* 38 261–266. 10.1016/j.pediatrneurol.2007.11.005 18358405

[B93] Chau V. Poskitt K. J. Miller S. P. (2012). Magnetic resonance imaging in hypoxic-ischemic encephalopathy: still a cool test. *Arch. Pediatr. Adolesc. Med.* 166 669–671. 10.1001/archpediatrics.2012.579 22751888

[B94] Chau V. Poskitt K. J. Sargent M. A. Lupton B. A. Hill A. Roland E. (2009). Comparison of computer tomography and magnetic resonance imaging scans on the third day of life in term newborns with neonatal encephalopathy. *Pediatrics* 123 319–326. 10.1542/peds.2008-0283 19117898

[B95] Chau V. Synnes A. Grunau R. E. Poskitt K. J. Brant R. Miller S. P. (2013). Abnormal brain maturation in preterm neonates associated with adverse developmental outcomes. *Neurology* 81 2082–2089. 10.1212/01.wnl.0000437298.43688.b9 24212394 PMC3863348

[B96] Chaudhari P. Ye Z. Jang Y. Y. (2014). Roles of reactive oxygen species in the fate of stem cells. *Antioxid. Redox Signal.* 20 1881–1890. 10.1089/ars.2012.4963 23066813 PMC3967382

[B97] Chavez-Valdez R. Martin L. J. Razdan S. Gauda E. B. Northington F. J. (2014). Sexual dimorphism in BDNF signaling after neonatal hypoxia-ischemia and treatment with necrostatin-1. *Neuroscience* 260 106–119. 10.1016/j.neuroscience.2013.12.023 24361177 PMC3950408

[B98] Chen C. J. Liao S. L. (2003). Zinc toxicity on neonatal cortical neurons: involvement of glutathione chelation. *J. Neurochem.* 85 443–453. 10.1046/j.1471-4159.2003.01691.x 12675920

[B99] Chen H. Spagnoli F. Burris M. Rolland W. B. Fajilan A. Dou H. (2012). Nanoerythropoietin is 10-times more effective than regular erythropoietin in neuroprotection in a neonatal rat model of hypoxia and ischemia. *Stroke* 43 884–887. 10.1161/STROKEAHA.111.637090 22156696

[B100] Cheng Y. Deshmukh M. D’Costa A. Demaro J. A. Gidday J. M. Shah A. (1998). Caspase inhibitor affords neuroprotection with delayed administration in a rat model of neonatal hypoxic-ischemic brain injury. *J. Clin. Invest.* 101 1992–1999. 10.1172/JCI2169 9576764 PMC508786

[B101] Chiappe-Gutierrez M. Kitzmueller E. Labudova O. Fuerst G. Hoeger H. Hardmeier R. (1998). mRNA levels of the hypoxia inducible factor (HIF-1) and DNA repair genes in perinatal asphyxia of the rat. *Life Sci.* 63 1157–1167. 10.1016/S0024-3205(98)00377-4 9763211

[B102] Choi D. W. (1988). Calcium-mediated neurotoxicity: relationship to specific channel types and role in ischemic damage. *Trends Neurosci.* 11 465–469. 10.1016/0166-2236(88)90200-7 2469166

[B103] Choi D. W. (1992). Excitotoxic cell death. *J. Neurobiol.* 23 1261–1276. 10.1002/neu.480230915 1361523

[B104] Choi H. A. Badjatia N. Mayer S. A. (2012). Hypothermia for acute brain injury—mechanisms and practical aspects. *Nat. Rev. Neurol.* 8 214–222. 10.1038/nrneurol.2012.21 22371279

[B105] Christaki E. Giamarellos-Bourboulis E. J. (2014). The beginning of personalized medicine in sepsis: small steps to a bright future. *Clin. Genet.* 86 56–61. 10.1111/cge.12368 24579691

[B106] Chun J. J. Nakamura M. J. Shatz C. J. (1987). Transient cells of the developing mammalian telencephalon are peptide-immunoreactive neurons. *Nature* 325 617–620. 10.1038/325617a0 3543691

[B107] Cinelli P. Madani R. Tsuzuki N. Vallet P. Arras M. Zhao C. N. (2001). Neuroserpin, a neuroprotective factor in focal ischemic stroke. *Mol. Cell. Neurosci.* 18 443–457. 10.1006/mcne.2001.1028 11922137

[B108] Clancy B. Finlay B. L. Darlington R. B. Anand K. J. (2007). Extrapolating brain development from experimental species to humans. *Neurotoxicology* 28 931–937. 10.1016/j.neuro.2007.01.014 17368774 PMC2077812

[B109] Clancy B. Teague-Ross T. J. Nagarajan R. (2009). Cross-species analyses of the cortical GABAergic and subplate neural populations. *Front. Neuroanat.* 3:20 10.3389/neuro.05.020.2009 PMC2779099 19936319

[B110] Cleary R. T. Sun H. Huynh T. Manning S. M. Li Y. Rotenberg A. (2013). Bumetanide enhances phenobarbital efficacy in a rat model of hypoxic neonatal seizures. *PLoS ONE* 8:e57148 10.1371/journal.pone.0057148 PMC3594228 23536761

[B111] Cohen M. C. Scheimberg I. (2008). Evidence of occurrence of intradural and subdural hemorrhage in the perinatal and neonatal period in the context of hypoxic ischemic encephalopathy: an observational study from two referral institutions in the United Kingdom. *Pediatr. Dev. Pathol.* 12 169–176. 10.2350/08-08-0509.1 19007301

[B112] Cole J. W. Naj A. C. O’Connell J. R. Stine O. C. Sorkin J. D. Wozniak M. A. (2007). Neuroserpin polymorphisms and stroke risk in a biracial population: the stroke prevention in young women study. *BMC Neurol.* 7:1 10.1186/1471-2377-7-37 PMC2169251 17961231

[B113] Coq J. O. Strata F. Russier M. Safadi F. F. Merzenich M. M. Byl N. N. (2008). Impact of neonatal asphyxia and hind limb immobilization on musculoskeletal tissues and S1 map organization: implications for cerebral palsy. *Exp. Neurol.* 210 95–108. 10.1016/j.expneurol.2007.10.006 18061167

[B114] Coutelier M. Andries S. Ghariani S. Dan B. Duyckaerts C. van Rijckevorsel K. (2008). Neuroserpin mutation causes electrical status epilepticus of slow-wave sleep. *Neurology* 71 64–66. 10.1212/01.wnl.0000316306.08751.28 18591508

[B115] Cowan F. Rutherford M. Groenendaal F. Eken P. Mercuri E. Bydder G. M. (2003). Origin and timing of brain lesions in term infants with neonatal encephalopathy. *Lancet* 361 736–742. 10.1016/S0140-6736(03)12658-X 12620738

[B116] Craig A. Ling Luo N. Beardsley D. J. Wingate-Pearse N. Walker D. W. Hohimer A. R. (2003). Quantitative analysis of perinatal rodent oligodendrocyte lineage progression and its correlation with human. *Exp. Neurol.* 181 231–240. 10.1016/S0014-4886(03)00032-3 12781996

[B117] Crawford M. A. Sinclair A. J. (1971). “Nutritional influences in the evolution of mammalian brain,” in *Proceedings of the Lipids, Malnutrition & the Developing Brain. Ciba Foundation Symposium*, Amsterdam, 267–292. 10.1002/9780470719862.ch16 4949878

[B118] Daadi M. M. Lee S. H. Arac A. Grueter B. A. Bhatnagar R. Maag A. L. (2009a). Functional engraftment of the medial ganglionic eminence cells in experimental stroke model. *Cell Transplant.* 18 815–826. 10.3727/096368909X470829 19500468

[B119] Daadi M. M. Li Z. Arac A. Grueter B. A. Sofilos M. Malenka R. C. (2009b). Molecular and magnetic resonance imaging of human embryonic stem cell-derived neural stem cell grafts in ischemic rat brain. *Mol. Ther.* 17 1282–1291. 10.1038/mt.2009.104 19436269 PMC2835224

[B120] Dalitz P. Harding R. Rees S. M. Cock M. L. (2003). Prolonged reductions in placental blood flow and cerebral oxygen delivery in preterm fetal sheep exposed to endotoxin: possible factors in white matter injury after acute infection. *J. Soc. Gynecol. Investig.* 10 283–290. 10.1016/S1071-5576(03)00090-X 12853089

[B121] Dammann O. Leviton A. (2014). Intermittent or sustained systemic inflammation and the preterm brain. *Pediatr. Res.* 75 376–380. 10.1038/pr.2013.238 24429547 PMC3943674

[B122] Dammann O. O’Shea T. M. (2008). Cytokines and perinatal brain damage. *Clin. Perinatol.* 35 643–663. 10.1016/j.clp.2008.07.011 19026332 PMC3657129

[B123] Danbolt N. C. (2001). Glutamate uptake. *Prog. Neurobiol.* 65 1–105. 10.1016/S0301-0082(00)00067-8 11369436

[B124] Daneman A. Epelman M. Blaser S. Jarrin J. R. (2006). Imaging of the brain in full-term neonates: does sonography still play a role? *Pediatr. Radiol.* 36 636–646. 10.1007/s00247-006-0201-7 16770668

[B125] Daneman A. Navarro O. M. Somers G. R. Mohanta A. Jarrín J. R. Traubici J. (2010). Renal pyramids: focused sonography of normal and pathologic processes 1. *Radiographics* 30 1287–1307. 10.1148/rg.305095222 20833851

[B126] Danysz W. Parsons C. G. (1998). Glycine and N-methyl-D-aspartate receptors: physiological significance and possible therapeutic applications. *Pharmacol. Rev.* 50 597–664. 9860805

[B127] Daval J. L. Pourie G. Grojean S. Lievre V. Strazielle C. Blaise S. (2004). Neonatal hypoxia triggers transient apoptosis followed by neurogenesis in the rat CA1 hippocampus. *Pediatr. Res.* 55 561–567. 10.1203/01.PDR.0000113771.51317.37 14739363

[B128] Daval J. L. Vert P. (2004). Apoptosis and neurogenesis after transient hypoxia in the developing rat brain. *Semin. Perinatol.* 28 257–263. 10.1053/j.semperi.2004.08.002 15565785

[B129] Davis R. L. Holohan P. D. Shrimpton A. E. Tatum A. H. Daucher J. Collins G. H. (1999a). Familial encephalopathy with neuroserpin inclusion bodies. *Am. J. Pathol.* 155 1901–1913. 10.1016/S0002-9440(10)65510-1 10595921 PMC3277299

[B130] Davis R. L. Shrimpton A. E. Holohan P. D. Bradshaw C. Feiglin D. Collins G. H. (1999b). Familial dementia caused by polymerization of mutant neuroserpin. *Nature* 401 376–379. 10517635 10.1038/43894

[B131] Davis R. L. Shrimpton A. E. Carrell R. W. Lomas D. A. Gerhard L. Baumann B. (2002). Association between conformational mutations in neuroserpin and onset and severity of dementia. *Lancet* 359 2242–2247. 10.1016/S0140-6736(02)09293-0 12103288

[B132] de Haan H. H. Gunn A. J. Gluckman P. D. (1997a). Fetal heart rate changes do not reflect cardiovascular deterioration during brief repeated umbilical cord occlusions in near-term fetal lambs. *Am. J. Obstet. Gynecol.* 176(1 Pt 1), 8–17. 10.1016/S0002-9378(97)80004-X 9024082

[B133] de Haan H. H. Gunn A. J. Williams C. E. Gluckman P. D. (1997b). Brief repeated umbilical cord occlusions cause sustained cytotoxic cerebral edema and focal infarcts in near-term fetal lambs. *Pediatr. Res.* 41 96–104. 8979296 10.1203/00006450-199701000-00015

[B134] de Haan H. H. Gunn A. J. Williams C. E. Heymann M. A. Gluckman P. D. (1997c). Magnesium sulfate therapy during asphyxia in near-term fetal lambs does not compromise the fetus but does not reduce cerebral injury. *Am. J. Obstet. Gynecol.* 176(1 Pt 1), 18–27. 9024083 10.1016/s0002-9378(97)80005-1

[B135] De Souza S. W. Richards B. (1978). Neurological sequelae in newborn babies after perinatal asphyxia. *Arch. Dis. Child.* 53 564–569. 10.1136/adc.53.7.564 686792 PMC1544975

[B136] de Torres C. Munell F. Ferrer I. Reventós J. Macaya A. (1997). Identification of necrotic cell death by the TUNEL assay in the hypoxic-ischemic neonatal rat brain. *Neurosci. Lett.* 230 1–4. 10.1016/S0304-3940(97)00445-X 9259449

[B137] de Vries L. S. Groenendaal F. (2010). Patterns of neonatal hypoxic-ischaemic brain injury. *Neuroradiology* 52 555–566. 10.1007/s00234-010-0674-9 20390260 PMC2872019

[B138] Dean J. M. Shi Z. Fleiss B. Gunn K. C. Groenendaal F. van Bel F. (2015). A critical review of models of perinatal infection. *Dev. Neurosci.* 37 289–304. 10.1159/000370309 25720344

[B139] Dean J. M. van de Looij Y. Sizonenko S. V. Lodygensky G. A. Lazeyras F. Bolouri H. (2011). Delayed cortical impairment following lipopolysaccharide exposure in preterm fetal sheep. *Ann. Neurol.* 70 846–856. 10.1002/ana.22480 22002627

[B140] Debillon T. Gras-Leguen C. Leroy S. Caillon J. Roze J. C. Gressens P. (2003). Patterns of cerebral inflammatory response in a rabbit model of intrauterine infection-mediated brain lesion. *Brain Res. Dev. Brain Res.* 145 39–48. 10.1016/S0165-3806(03)00193-7 14519492

[B141] Decker M. B. Breitburg D. L. Marcus N. H. (2003). Geographical differences in behavioral responses to hypoxia: local adaptation to an anthropogenic stressor? *Ecol. Appl.* 13 1104–1109. 10.1890/1051-0761(2003)13[1104:gdibrt]2.0.co;2

[B142] Decker M. J. Hue G. Caudle W. Miller G. Keating G. Rye D. (2003). Episodic neonatal hypoxia evokes executive dysfunction and regionally specific alterations in markers of dopamine signaling. *Neuroscience* 117 417–425. 10.1016/S0306-4522(02)00805-9 12614682

[B143] Decker M. J. Jones K. A. Solomon I. G. Keating G. L. Rye D. B. (2005). Reduced extracellular dopamine and increased responsiveness to novelty: neurochemical and behavioral sequelae of intermittent hypoxia. *Sleep* 28 169–176. 16171240 10.1093/sleep/28.2.169

[B144] Dell’Anna E. Geloso M. C. Magarelli M. Molinari M. (1996). Development of GABA and calcium binding proteins immunoreactivity in the rat hippocampus following neonatal anoxia. *Neurosci. Lett.* 211 93–96. 10.1016/0304-3940(96)12733-6 8830852

[B145] Dell’Anna M. Calzolari S. Molinari M. Iuvone L. Calimici R. (1991). Neonatal anoxia induces transitory hyperactivity, permanent spatial memory deficits and CA1 cell density reduction in developing rats. *Behav. Brain Res.* 45 125–134. 10.1016/S0166-4328(05)80078-6 1789921

[B146] Dell’Anna M. E. Geloso M. C. Draisci G. Luthman J. (1995). Transient changes in Fos and GFAP immunoreactivity precede neuronal loss in the rat hippocampus following neonatal anoxia. *Exp. Neurol.* 131 144–156. 10.1016/0014-4886(95)90016-0 7895808

[B147] Dell’Anna M. E. Luthman J. Lindqvist E. Olson L. (1993). Development of monoamine systems after neonatal anoxia in rats. *Brain Res. Bull.* 32 159–170. 10.1016/0361-9230(93)90070-R 8348340

[B148] Demarest T. G. Schuh R. A. Waite E. L. Waddell J. McKenna M. C. Fiskum G. (2016a). Sex dependent alterations in mitochondrial eectron transport chain proteins following neonatal rat cerebral hypoxic-ischemia. *J. Bioenerg. Biomembr.* 48 591–598. 10.1007/s10863-016-9678-4 27683241

[B149] Demarest T. G. Waite E. L. Kristian T. Puche A. C. Waddell J. McKenna M. C. (2016b). Sex-dependent mitophagy and neuronal death following rat neonatal hypoxia-ischemia. *Neuroscience* 335 103–113. 10.1016/j.neuroscience.2016.08.026 27555552 PMC5580242

[B150] Deng W. Rosenberg P. A. Volpe J. J. Jensen F. E. (2003). Calcium-permeable AMPA/kainate receptors mediate toxicity and preconditioning by oxygen-glucose deprivation in oligodendrocyte precursors. *Proc. Natl. Acad. Sci. U.S.A.* 100 6801–6806. 10.1073/pnas.1136624100 12743362 PMC164527

[B151] Deng Y. Y. Lu J. Ling E. A. Kaur C. (2009). Monocyte chemoattractant protein-1 (MCP-1) produced via NF-kappaB signaling pathway mediates migration of amoeboid microglia in the periventricular white matter in hypoxic neonatal rats. *Glia* 57 604–621. 10.1002/glia.20790 18942743

[B152] Derrick M. Drobyshevsky A. Ji X. Tan S. (2007). A model of cerebral palsy from fetal hypoxia-ischemia. *Stroke* 38(Suppl.), 731–735. 10.1161/01.STR.0000251445.94697.64 17261727

[B153] Derrick M. Luo N. L. Bregman J. C. Jilling T. Ji X. Fisher K. (2004). Preterm fetal hypoxia-ischemia causes hypertonia and motor deficits in the neonatal rabbit: a model for human cerebral palsy? *J. Neurosci.* 24 24–34. 10.1523/JNEUROSCI.2816-03.2004 14715934 PMC6729589

[B154] Dilenge M. E. Majnemer A. Shevell M. I. (2001). Long-term developmental outcome of asphyxiated term neonates. *J. Child Neurol.* 16 781–792. 10.1177/08830738010160110201 11732762

[B155] Dingley J. Tooley J. Liu X. Scull-Brown E. Elstad M. Chakkarapani E. (2014). Xenon ventilation during therapeutic hypothermia in neonatal encephalopathy: a feasibility study. *Pediatrics* 133 809–818. 10.1542/peds.2013-0787 24777219

[B156] Ditelberg J. S. Sheldon R. A. Epstein C. J. Ferriero D. M. (1996). Brain injury after perinatal hypoxia-ischemia is exacerbated in copper/zinc superoxide dismutase transgenic mice. *Pediatr. Res.* 39 204–208. 10.1203/00006450-199602000-00003 8825788

[B157] Dommergues M. Plaisant F. Verney C. Gressens P. (2003). Early microglial activation following neonatal excitotoxic brain damage in mice: a potential target for neuroprotection. *Neuroscience* 121 619–628. 10.1016/S0306-4522(03)00558-X 14568022

[B158] Dommergues M. A. Patkai J. Renauld J. C. Evrard P. Gressens P. (2000). Proinflammatory cytokines and interleukin-9 exacerbate excitotoxic lesions of the newborn murine neopallium. *Ann. Neurol.* 47 54–63. 10.1002/1531-8249(200001)47:1<54::AID-ANA10>3.0.CO;2-Y 10632101

[B159] Douglas-Escobar M. Weiss M. D. (2015). Hypoxic-ischemic encephalopathy: a review for the clinician. *JAMA Pediatr.* 169 397–403. 10.1001/jamapediatrics.2014.3269 25685948

[B160] Doyle O. Temko A. Murray D. Lightbody G. Marnane W. Boylan G. (2010). “Predicting the neurodevelopmental outcome in newborns with hypoxic-ischaemic injury,” in *Proceedings of the Engineering in Medicine and Biology Society (EMBC), 2010 Annual International Conference of the IEEE*, Buenos Aires, 1370–1373. 10.1109/IEMBS.2010.5626736 21096334

[B161] Drobyshevsky A. Derrick M. Prasad P. V. Ji X. Englof I. Tan S. (2007a). Fetal brain magnetic resonance imaging response acutely to hypoxia-ischemia predicts postnatal outcome. *Ann. Neurol.* 61 307–314. 17444507 10.1002/ana.21095

[B162] Drobyshevsky A. Derrick M. Wyrwicz A. M. Ji X. Englof I. Ullman L. M. (2007b). White matter injury correlates with hypertonia in an animal model of cerebral palsy. *J. Cereb. Blood Flow Metab.* 27 270–281. 16736047 10.1038/sj.jcbfm.9600333

[B163] Drobyshevsky A. Song S. K. Gamkrelidze G. Wyrwicz A. M. Derrick M. Meng F. (2005). Developmental changes in diffusion anisotropy coincide with immature oligodendrocyte progression and maturation of compound action potential. *J. Neurosci.* 25 5988–5997. 10.1523/JNEUROSCI.4983-04.2005 15976088 PMC6724805

[B164] Du L. Hickey R. W. Bayir H. Watkins S. C. Tyurin V. A. Guo F. (2009). Starving neurons show sex difference in autophagy. *J. Biol. Chem.* 284 2383–2396. 10.1074/jbc.M804396200 19036730 PMC2629091

[B165] D’Udine B. Alleva E. (1988). The *Acomys cahirinus* (spiny mouse) as a new model for biological and neurobehavioural studies. *Pol. J. Pharmacol. Pharm.* 40 525–534. 3075753

[B166] Duncan J. R. Cock M. L. Scheerlinck J. P. Westcott K. T. McLean C. Harding R. (2002). White matter injury after repeated endotoxin exposure in the preterm ovine fetus. *Pediatr. Res.* 52 941–949. 10.1203/00006450-200212000-00021 12438674

[B167] East C. E. Dunster K. R. Colditz P. B. (1998). Fetal oxygen saturation and uterine contractions during labor. *Am. J. Perinatol.* 15 345–349. 10.1055/s-2007-993955 9722053

[B168] Edwards A. Mehmet H. (1996). Apoptosis in perinatal hypoxic-ischaemic cerebral damage. *Neuropathol. Appl. Neurobiol.* 22 494–498. 10.1111/j.1365-2990.1996.tb01122.x 9004235

[B169] Edwards A. Yue X. Cox P. Hope P. Azzopardi D. Squier M. (1997). Apoptosis in the brains of infants suffering intrauterine cerebral injury. *Pediatr. Res.* 42 684–689. 10.1203/00006450-199711000-00022 9357944

[B170] Eicher D. J. Wagner C. L. Katikaneni L. P. Hulsey T. C. Bass W. T. Kaufman D. A. (2005). Moderate hypothermia in neonatal encephalopathy: efficacy outcomes. *Pediatr. Neurol.* 32 11–17. 10.1016/j.pediatrneurol.2004.06.014 15607598

[B171] Ek C. J. Dziegielewska K. M. Stolp H. Saunders N. R. (2006). Functional effectiveness of the blood-brain barrier to small water-soluble molecules in developing and adult opossum (*Monodelphis domestica*). *J. Comp. Neurol.* 496 13–26. 10.1002/cne.20885 16528724 PMC2634607

[B172] Ek C. J. Habgood M. D. Dziegielewska K. M. Saunders N. R. (2003). Structural characteristics and barrier properties of the choroid plexuses in developing brain of the opossum (*Monodelphis domestica*). *J. Comp. Neurol.* 460 451–464. 10.1002/cne.10661 12717706

[B173] Eken P. Jansen G. H. Groenendaal F. Rademaker K. J. de Vries L. S. (1994). Intracranial lesions in the fullterm infant with hypoxic ischaemic encephalopathy: ultrasound and autopsy correlation. *Neuropediatrics* 25 301–307. 10.1055/s-2008-1073044 7770127

[B174] Eklind S. Mallard C. Arvidsson P. Hagberg H. (2005). Lipopolysaccharide induces both a primary and a secondary phase of sensitization in the developing rat brain. *Pediatr. Res.* 58 112–116. 10.1203/01.PDR.0000163513.03619.8D 15879289

[B175] Eklind S. Mallard C. Leverin A. L. Gilland E. Blomgren K. Mattsby-Baltzer I. (2001). Bacterial endotoxin sensitizes the immature brain to hypoxic–ischaemic injury. *Eur. J. Neurosci.* 13 1101–1106. 10.1046/j.0953-816x.2001.01474.x 11285007

[B176] Eklöf B. Siesjö B. K. (1973). Cerebral blood flow in ischemia caused by carotid artery ligation in the rat. *Acta Physiol. Scand.* 87 69–77. 10.1111/j.1748-1716.1973.tb05367.x 4687343

[B177] El-Khoury N. Braun A. Hu F. Pandey M. Nedergaard M. Lagamma E. F. (2006). Astrocyte end-feet in germinal matrix, cerebral cortex, and white matter in developing infants. *Pediatr. Res.* 59 673–679. 10.1203/01.pdr.0000214975.85311.9c 16627880

[B178] Ellisdon A. M. Zhang Q. Henstridge M. A. Johnson T. K. Warr C. G. Law R. H. (2014). High resolution structure of cleaved Serpin 42 Da from *Drosophila melanogaster*. *BMC Struct. Biol.* 14:1 10.1186/1472-6807-14-14 PMC4006314 24758516

[B179] Eskiocak S. Tutunculer F. Basaran U. N. Taskiran A. Cakir E. (2007). The effect of melatonin on protein oxidation and nitric oxide in the brain tissue of hypoxic neonatal rats. *Brain Dev.* 29 19–24. 10.1016/j.braindev.2006.05.007 16843629

[B180] Evans D. J. Levene M. I. Tsakmakis M. (2007). Anticonvulsants for preventing mortality and morbidity in full term newborns with perinatal asphyxia. *Cochrane Database Syst. Rev.* 3:CD001240 10.1002/14651858.CD001240.pub2 17636659

[B181] Ezquer M. E. Valdez S. R. Seltzer A. M. (2006). Inflammatory responses of the substantia nigra after acute hypoxia in neonatal rats. *Exp. Neurol.* 197 391–398. 10.1016/j.expneurol.2005.10.015 16293246

[B182] Ezquer M. E. Valdez S. R. Seltzer A. M. Jahn G. A. (2008). Advancement of reproductive senescence and changes in the early expression of estrogen, progesterone and micro-opioid receptors induced by neonatal hypoxia in the female rat. *Brain Res.* 1214 73–83. 10.1016/j.brainres.2008.03.029 18457817

[B183] Fahn S. Davis J. N. Rowland L. P. (1979). *Cerebral Hypoxia and Its Consequences.* New York, NY: Raven Press.

[B184] Failor S. Nguyen V. Darcy D. P. Cang J. Wendland M. F. Stryker M. P. (2010). Neonatal cerebral hypoxia-ischemia impairs plasticity in rat visual cortex. *J. Neurosci.* 30 81–92. 10.1523/JNEUROSCI.5656-08.2010 20053890 PMC2822440

[B185] Fan X. Kavelaars A. Heijnen C. J. Groenendaal F. van Bel F. (2010). Pharmacological neuroprotection after perinatal hypoxic-ischemic brain injury. *Curr. Neuropharmacol.* 8 324–334. 10.2174/157015910793358150 21629441 PMC3080590

[B186] Fatemi A. Wilson M. A. Johnston M. V. (2009). Hypoxic-ischemic encephalopathy in the term infant. *Clin. Perinatol.* 36 835–vii 10.1016/j.clp.2009.07.011 PMC2849741 19944838

[B187] Favrais G. van de Looij Y. Fleiss B. Ramanantsoa N. Bonnin P. Stoltenburg-Didinger G. (2011). Systemic inflammation disrupts the developmental program of white matter. *Ann. Neurol.* 70 550–565. 10.1002/ana.22489 21796662

[B188] Felderhoff-Mueser U. Sifringer M. Pesditschek S. Kuckuck H. Moysich A. Bittigau P. (2002). Pathways leading to apoptotic neurodegeneration following trauma to the developing rat brain. *Neurobiol. Dis.* 11 231–245. 10.1006/nbdi.2002.0521 12505417

[B189] Fellman V. Hellström-Westas L. Norman M. Westgren M. Källén K. Lagercrantz H. (2010). One-year survival of extremely preterm infants after active perinatal care in Sweden. *Obstet. Anesth. Dig.* 30 22–23. 10.1097/01.aoa.0000367003.25266.35 19491184

[B190] Ferrari D. C. Nesic O. Perez-Polo J. R. (2010). Perspectives on neonatal hypoxia/ischemia-induced edema formation. *Neurochem. Res.* 35 1957–1965. 10.1007/s11064-010-0308-y 21136160

[B191] Ferriero D. M. (2001). Oxidant mechanisms in neonatal hypoxia-ischemia. *Dev. Neurosci.* 23 198–202. 10.1159/000046143 11598320

[B192] Ferriero D. M. (2004). Neonatal brain injury. *N. Engl. J. Med.* 351 1985–1995. 10.1056/NEJMra041996 15525724

[B193] Ferriero D. M. Holtzman D. M. Black S. M. Sheldon R. A. (1996). Neonatal mice lacking neuronal nitric oxide synthase are less vulnerable to hypoxic–ischemic injury. *Neurobiol. Dis.* 3 64–71. 10.1006/nbdi.1996.0006 9173913

[B194] Finer N. Robertson C. Richards R. Pinnell L. Peters K. (1981). Hypoxic-ischemic encephalopathy in term neonates: perinatal factors and outcome. *J. Pediatr.* 98 112–117. 10.1016/S0022-3476(81)80555-0 7452386

[B195] Finer N. N. Robertson C. M. Peters K. L. Coward J. H. (1983). Factors affecting outcome in hypoxic-ischemic encephalopathy in term infants. *Am. J. Dis. Child.* 137 21–25. 10.1001/archpedi.1983.02140270017006 6847953

[B196] Fischer R. Maier O. (2015). Interrelation of oxidative stress and inflammation in neurodegenerative disease: role of TNF. *Oxid. Med. Cell. Longev.* 2015:610813 10.1155/2015/610813 PMC4365363 25834699

[B197] Fleiss B. Gressens P. (2012). Tertiary mechanisms of brain damage: a new hope for treatment of cerebral palsy? *Lancet Neurol.* 11 556–566. 10.1016/S1474-4422(12)70058-3 22608669

[B198] Folkerth R. D. (2005). Neuropathologic substrate of cerebral palsy. *J. Child Neurol.* 20 940–949. 10.1177/08830738050200120301 16417840

[B199] Forder J. P. Tymianski M. (2009). Postsynaptic mechanisms of excitotoxicity: involvement of postsynaptic density proteins, radicals, and oxidant molecules. *Neuroscience* 158 293–300. 10.1016/j.neuroscience.2008.10.021 19041375

[B200] Fox K. Schlaggar B. L. Glazewski S. O’Leary D. D. (1996). Glutamate receptor blockade at cortical synapses disrupts development of thalamocortical and columnar organization in somatosensory cortex. *Proc. Natl. Acad. Sci. U.S.A.* 93 5584–5589. 10.1073/pnas.93.11.5584 8643619 PMC39290

[B201] Fujihara M. Muroi M. Tanamoto K. Suzuki T. Azuma H. Ikeda H. (2003). Molecular mechanisms of macrophage activation and deactivation by lipopolysaccharide: roles of the receptor complex. *Pharmacol. Ther.* 100 171–194. 10.1016/j.pharmthera.2003.08.003 14609719

[B202] Fujimoto E. Miki A. Mizoguti H. (1989). Histochemical study of the differentiation of microglial cells in the developing human cerebral hemispheres. *J. Anat.* 166 253–264. 2621143 PMC1256758

[B203] Galliciotti G. Sonderegger P. (2006). Neuroserpin. *Front. Biosci.* 11: 33–45. 10.2741/1778 16146712

[B204] Garcia-Marin V. Blazquez-Llorca L. Rodriguez J. R. Gonzalez-Soriano J. DeFelipe J. (2010). Differential distribution of neurons in the gyral white matter of the human cerebral cortex. *J. Comp. Neurol.* 518 4740–4759. 10.1002/cne.22485 20963826

[B205] Garnier Y. Berger R. Alm S. von Duering M. U. Coumans A. B. Michetti F. (2006). Systemic endotoxin administration results in increased S100B protein blood levels and periventricular brain white matter injury in the preterm fetal sheep. *Eur. J. Obstet. Gynecol. Reprod. Biol.* 124 15–22. 10.1016/j.ejogrb.2005.05.014 16386654

[B206] Garnier Y. Coumans A. Berger R. Jensen A. Hasaart T. H. (2001). Endotoxemia severely affects circulation during normoxia and asphyxia in immature fetal sheep. *J. Soc. Gynecol. Investig.* 8 134–142. 10.1016/S1071-5576(01)00101-0 11390247

[B207] Gelderblom M. Neumann M. Ludewig P. Bernreuther C. Krasemann S. Arunachalam P. (2013). Deficiency in serine protease inhibitor neuroserpin exacerbates ischemic brain injury by increased postischemic inflammation. *PLoS ONE* 8:e63118 10.1371/journal.pone.0063118 PMC3643909 23658802

[B208] Ghosh A. Antonini A. McConnell S. K. Shatz C. J. (1990). Requirement for subplate neurons in the formation of thalamocortical connections. *Nature* 347 179–181. 10.1038/347179a0 2395469

[B209] Ghosh A. Shatz C. J. (1992). Involvement of subplate neurons in the formation of ocular dominance columns. *Science* 255 1441–1443. 10.1126/science.1542795 1542795

[B210] Gillani Q. A. Akbar A. Ali M. Iqbal F. (2015). Gender-specific effects of CGP55845, GABAB receptor antagonist, on neuromuscular coordination, learning and memory formation in albino mouse following neonatalhypoxia–ischemia insult. *Neurol. Sci.* 36 961–969. 10.1007/s10072-015-2205-2 25847084

[B211] Gillani Q. A. Iqbal S. Arfa F. Khakwani S. Akbar A. Ullah A. (2014). Effect of GABA B receptor antagonist (CGP35348) on learning and memory in albino mice. *Sci. World J.* 2014:983651 10.1155/2014/983651 PMC3916030 24574938

[B212] Ginet V. Puyal J. Clarke P. G. Truttmann A. C. (2009). Enhancement of autophagic flux after neonatal cerebral hypoxia-ischemia and its region-specific relationship to apoptotic mechanisms. *Am. J. Pathol.* 175 1962–1974. 10.2353/ajpath.2009.090463 19815706 PMC2774060

[B213] Girard S. Kadhim H. Beaudet N. Sarret P. Sebire G. (2009). Developmental motor deficits induced by combined fetal exposure to lipopolysaccharide and early neonatal hypoxia/ischemia: a novel animal model for cerebral palsy in very premature infants. *Neuroscience* 158 673–682. 10.1016/j.neuroscience.2008.10.032 19010395

[B214] Girard S. Kadhim H. Larouche A. Roy M. Gobeil F. Sebire G. (2008). Pro-inflammatory disequilibrium of the IL-1 beta/IL-1ra ratio in an experimental model of perinatal brain damages induced by lipopolysaccharide and hypoxia-ischemia. *Cytokine* 43 54–62. 10.1016/j.cyto.2008.04.007 18511291

[B215] Girard S. Sebire H. Brochu M. E. Briota S. Sarret P. Sebire G. (2012). Postnatal administration of IL-1Ra exerts neuroprotective effects following perinatal inflammation and/or hypoxic-ischemic injuries. *Brain Behav. Immun.* 26 1331–1339. 10.1016/j.bbi.2012.09.001 22982341 PMC5023428

[B216] Glenn O. A. Ludeman N. A. Berman J. I. Wu Y. W. Lu Y. Bartha A. I. (2007). Diffusion tensor MR imaging tractography of the pyramidal tracts correlates with clinical motor function in children with congenital hemiparesis. *AJNR. Am. J. Neuroradiol.* 28 1796–1802. 10.3174/ajnr.A0676 17893220 PMC8134215

[B217] Gluckman P. D. Wyatt J. S. Azzopardi D. Ballard R. Edwards A. D. Ferriero D. M. (2005). Selective head cooling with mild systemic hypothermia after neonatal encephalopathy: multicentre randomised trial. *Lancet* 365 663–670. 10.1016/S0140-6736(05)17946-X 15721471

[B218] Goldberg R. N. Moscoso P. Bauer C. R. Bloom F. L. Curless R. G. Burke B. (1986). Use of barbiturate therapy in severe perinatal asphyxia: a randomized controlled trial. *J. Pediatr.* 109 851–856. 10.1016/S0022-3476(86)80713-2 3534201

[B219] Gonzalez F. F. Miller S. P. (2006). Does perinatal asphyxia impair cognitive function without cerebral palsy? *Arch. Dis. Child. Fetal Neonatal Ed.* 91 F454–F459. 17056843 10.1136/adc.2005.092445 PMC2672766

[B220] González-Portillo A. Domínguez-Antolínez I. Muniategui-Azkona E. (2015). Crisis of the welfare state: an analysis of the responses from social work. *Rev. Cercetare Interventie Soc.* 49 173–186.

[B221] Gozzo S. Dimitrieva N. Iacopino C. D’Udine B. (1985). A comparative study of mossy fiber distribution in the brain of the precocial *Acomys cahirinus* and of the altricial *Rattus norvegicus*: neuroanatomical bases and behavioral correlates. *Int. J. Neurosci.* 28 163–172. 10.3109/00207458508985387 2419270

[B222] Graham E. M. Ruis K. A. Hartman A. L. Northington F. J. Fox H. E. (2008). A systematic review of the role of intrapartum hypoxia-ischemia in the causation of neonatal encephalopathy. *Am. J. Obstet. Gynecol.* 199 587–595. 10.1016/j.ajog.2008.06.094 19084096

[B223] Grant E. Hoerder-Suabedissen A. Molnár Z. (2016). The regulation of corticofugal fiber targeting by retinal inputs. *Cereb. Cortex* 26 1336–1348. 10.1093/cercor/bhv315 26744542 PMC4737616

[B224] Greggio S. de Paula S. de Oliveira I. M. Trindade C. Rosa R. M. Henriques J. A. (2011). NAP prevents acute cerebral oxidative stress and protects against long-term brain injury and cognitive impairment in a model of neonatal hypoxia–ischemia. *Neurobiol. Dis.* 44 152–159. 10.1016/j.nbd.2011.06.018 21757007

[B225] Greggio S. Rosa R. M. Dolganov A. de Oliveira I. M. Menegat F. D. Henriques J. A. (2009). NAP prevents hippocampal oxidative damage in neonatal rats subjected to hypoxia-induced seizures. *Neurobiol. Dis.* 36 435–444. 10.1016/j.nbd.2009.08.008 19703564

[B226] Groenendaal F. de Vries L. S. van Bel F. (2006). Favourable results with surgical treatment in 43 children with hypoplastic left-heart syndrome or similar disorders, 1999-2005. *Ned. Tijdschr. Geneeskd.* 150 2731–2732. 17195321

[B227] Groenendaal F. Meiners L. C. Gooskens R. de Vries L. S. (1994). Cerebral proton magnetic resonance spectroscopic imaging in a neonate with tuberous sclerosis. *Neuropediatrics* 25 154–157. 10.1055/s-2008-1073014 7969798

[B228] Grow J. Barks J. D. (2002). Pathogenesis of hypoxic-ischemic cerebral injury in the term infant: current concepts. *Clin. Perinatol.* 29 585–602. 10.1016/S0095-5108(02)00059-3 12516737

[B229] Gu R. Fu L. Jiang C. Xu Y. Wang X. Yu J. (2015). Retina is protected by neuroserpin from ischemic/reperfusion-induced injury independent of tissue-type plasminogen activator. *PLoS ONE* 10:e0130440 10.1371/journal.pone.0130440 PMC4503687 26176694

[B230] Gunn A. J. Battin M. Gluckman P. D. Gunn T. R. Bennet L. (2005). Therapeutic hypothermia: from lab to NICU. *J. Perinat. Med.* 33 340–346. 10.1515/JPM.2005.061 16207121

[B231] Gunn A. J. Gunn T. R. (1998). Thepharmacology’of neuronal rescue with cerebral hypothermia. *Early Hum. Dev.* 53 19–35. 10.1016/S0378-3782(98)00033-4 10193924

[B232] Gunn A. J. Gunn T. R. de Haan H. H. Williams C. E. Gluckman P. D. (1997). Dramatic neuronal rescue with prolonged selective head cooling after ischemia in fetal lambs. *J. Clin. Invest.* 99 248–256. 10.1172/JCI119153 9005993 PMC507792

[B233] Gunn A. J. Parer J. T. Mallard E. C. Williams C. E. Gluckman P. D. (1992). Cerebral histologic and electrocorticographic changes after asphyxia in fetal sheep. *Pediatr. Res.* 31 486–491. 10.1203/00006450-199205000-00016 1603625

[B234] Hack M. Breslau N. Aram D. Weissman B. Klein N. Borawski-Clark E. (1992). The effect of very low birth weight and social risk on neurocognitive abilities at school age. *J. Dev. Behav. Pediatr.* 13 412–420. 10.1097/00004703-199212000-00005 1469109

[B235] Hadjiconstantinou M. Yates A. J. Neff N. H. (1990). Hypoxia-induced neurotransmitter deficits in neonatal rats are partially corrected by exogenous GM1 ganglioside. *J. Neurochem.* 55 864–869. 10.1111/j.1471-4159.1990.tb04571.x 1696622

[B236] Hagberg H. Andersson P. Kjellmer I. Thiringer K. Thordstein M. (1987). Extracellular overflow of glutamate, aspartate, GABA and taurine in the cortex and basal ganglia of fetal lambs during hypoxia-ischemia. *Neurosci. Lett.* 78 311–317. 10.1016/0304-3940(87)90379-X 2888062

[B237] Hagberg H. Gressens P. Mallard C. (2012). Inflammation during fetal and neonatal life: implications for neurologic and neuropsychiatric disease in children and adults. *Ann. Neurol.* 71 444–457. 10.1002/ana.22620 22334391

[B238] Hagberg H. Mallard C. Ferriero D. M. Vannucci S. J. Levison S. W. Vexler Z. S. (2015). The role of inflammation in perinatal brain injury. *Nat. Rev. Neurol.* 11 192–208. 10.1038/nrneurol.2015.13 25686754 PMC4664161

[B239] Hagberg H. Mallard C. Rousset C. I. Xiaoyang W. (2009). Apoptotic mechanisms in the immature brain: involvement of mitochondria. *J. Child Neurol.* 24 1141–1146. 10.1177/0883073809338212 19574577 PMC3674552

[B240] Hagberg H. Peebles D. Mallard C. (2002). Models of white matter injury: comparison of infectious, hypoxic-ischemic, and excitotoxic insults. *Ment. Retard. Dev. Disabil. Res. Rev.* 8 30–38. 10.1002/mrdd.10007 11921384

[B241] Hagberg H. Thornberg E. Blennow M. Kjellmer I. Lagercrantz H. Thiringer K. (1993). Excitatory amino acids in the cerebrospinal fluid of asphyxiated infants: relationship to hypoxic-ischemic encephalopathy. *Acta Paediatr.* 82 925–929. 10.1111/j.1651-2227.1993.tb12601.x 7906573

[B242] Hall R. T. Hall F. K. Daily D. K. (1998). High-dose phenobarbital therapy in term newborn infants with severe perinatal asphyxia: a randomized, prospective study with three-year follow-up. *J. Pediatr.* 132 345–348. 10.1016/S0022-3476(98)70458-5 9506654

[B243] Hamre K. M. Hyman B. T. Goodlett C. R. West J. R. Van Hoesen G. W. (1989). Alz-50 immunoreactivity in the neonatal rat: changes in development and co-distribution with MAP-2 immunoreactivity. *Neurosci. Lett.* 98 264–271. 10.1016/0304-3940(89)90411-4 2657503

[B244] Hamrick S. E. Ferriero D. M. (2003). The injury response in the term newborn brain: can we neuroprotect? *Curr. Opin. Neurol.* 16 147–154. 10.1097/00019052-200304000-00005 12644741

[B245] Hardy P. Nuyt A. M. Dumont I. Peri K. G. Hou X. Varma D. R. (1999). Developmentally increased cerebrovascular NO in newborn pigs curtails cerebral blood flow autoregulation. *Pediatr. Res.* 46 375–382. 10.1203/00006450-199910000-00004 10509356

[B246] Hasegawa K. Yoshioka H. Sawada T. Nishikawa H. (1991). Lipid peroxidation in neonatal mouse brain subjected to two different types of hypoxia. *Brain Dev.* 13 101–103. 10.1016/S0387-7604(12)80115-X 1909842

[B247] Hasegawa K. Yoshioka H. Sawada T. Nishikawa H. (1993). Direct measurement of free radicals in the neonatal mouse brain subjected to hypoxia: an electron spin resonance spectroscopic study. *Brain Res.* 607 161–166. 10.1016/0006-8993(93)91502-J 8386973

[B248] Hastings G. A. Coleman T. A. Haudenschild C. C. Stefansson S. Smith E. P. Barthlow R. (1997). Neuroserpin, a brain-associated inhibitor of tissue plasminogen activator is localized primarily in neurons. Implications for the regulation of motor learning and neuronal survival. *J. Biol. Chem.* 272 33062–33067. 10.1074/jbc.272.52.33062 9407089

[B249] Hasumi H. Ishiguro H. Nakamura M. Sugiura S. Osada Y. Miyoshi Y. (2005). Neuroserpin (PI-12) is upregulated in high-grade prostate cancer and is associated with survival. *Int. J. Cancer* 115 911–916. 10.1002/ijc.20967 15723353

[B250] Hattori H. Wasterlain C. G. (1990). Excitatory amino acids in the developing brain: ontogeny, plasticity, and excitotoxicity. *Pediatr. Neurol.* 6 219–228. 10.1016/0887-8994(90)90111-D 2169749

[B251] Hedner T. Bergman B. Holmgren M. (1980). Adrenal catecholamines during and following hypoxia in neonatal rats. *Med. Biol.* 58 228–231. 7266082

[B252] Hedner T. Lundborg P. (1980a). 5-HIAA levels in brain and cerebrospinal fluid of the neonatal rat during oxygen deprivation. *Neurosci. Lett.* 19 315–318. 6189029 10.1016/0304-3940(80)90280-3

[B253] Hedner T. Lundborg P. (1980b). Catecholamine metabolism in neonatal rat brain during asphyxia and recovery. *Acta Physiol. Scand.* 109 169–175. 10.1111/j.1748-1716.1980.tb06583.x 6775492

[B254] Hedner T. Lundborg P. (1980c). Serotonin metabolism in neonatal rat brain during asphyxia and recovery. *Acta Physiol. Scand.* 109 163–168. 6968497 10.1111/j.1748-1716.1980.tb06582.x

[B255] Hedtjarn M. Mallard C. Eklind S. Gustafson-Brywe K. Hagberg H. (2004a). Global gene expression in the immature brain after hypoxia-ischemia. *J. Cereb. Blood Flow Metab.* 24 1317–1332. 10.1097/01.WCB.0000141558.40491.75 15625407

[B256] Hedtjarn M. Mallard C. Hagberg H. (2004b). Inflammatory gene profiling in the developing mouse brain after hypoxia-ischemia. *J. Cereb. Blood Flow Metab.* 24 1333–1351. 15625408 10.1097/01.WCB.0000141559.17620.36

[B257] Helmy M. M. Tolner E. A. Vanhatalo S. Voipio J. Kaila K. (2011). Brain alkalosis causes birth asphyxia seizures, suggesting therapeutic strategy. *Ann. Neurol.* 69 493–500. 10.1002/ana.22223 21337602

[B258] Henry V. J. Lecointre M. Laudenbach V. Ali C. Macrez R. Jullienne A. (2013). High tPA release by neonate brain microvascular endothelial cells under glutamate exposure affects neuronal fate. *Neurobiol. Dis.* 50 201–208. 10.1016/j.nbd.2012.10.020 23103420

[B259] Herrmann K. Antonini A. Shatz C. J. (1994). Ultrastructural evidence for synaptic interactions between thalamocortical axons and subplate neurons. *Eur. J. Neurosci.* 6 1729–1742. 10.1111/j.1460-9568.1994.tb00565.x 7874312

[B260] Hida K. Suzuki N. Kwee I. L. Nakada T. (1991). pH-lactate dissociation in neonatal anoxia: proton and 31P NMR spectroscopic studies in rat pups. *Magn. Reson. Med.* 22 128–132. 10.1002/mrm.1910220113 1798387

[B261] Hill A. (1991). Current concepts of hypoxic-ischemic cerebral injury in the term newborn. *Pediatr. Neurol.* 7 317–325. 10.1016/0887-8994(91)90060-X 1764132

[B262] Hill C. A. Alexander M. L. McCullough L. D. Fitch R. H. (2012). Inhibition of X-linked inhibitor of apoptosis with embelin differentially affects male versus female behavioral outcome following neonatal hypoxia-ischemia in rats. *Dev. Neurosci.* 33 494–504. 10.1159/000331651 PMC3357172 22041713

[B263] Hill C. A. Fitch R. H. (2012). Sex differences in mechanisms and outcome of neonatal hypoxia-ischemia in rodent models: implications for sex-specific neuroprotection in clinical neonatal practice. *Neurol. Res. Int.* 2012:867531 10.1155/2012/867531 PMC3306914 22474588

[B264] Hill I. E. MacManus J. P. Rasquinha I. Tuor U. I. (1995). DNA fragmentation indicative of apoptosis following unilateral cerebral hypoxia-ischemia in the neonatal rat. *Brain Res.* 676 398–403. 10.1016/0006-8993(95)00145-G 7614012

[B265] Hill R. M. Brennan S. O. Birch N. P. (2001). Expression, purification, and functional characterization of the serine protease inhibitor neuroserpin expressed in Drosophila S2 cells. *Protein Expr. Purif.* 22 406–413. 10.1006/prep.2001.1463 11483002

[B266] Himmelmann K. Hagberg G. Beckung E. Hagberg B. Uvebrant P. (2005). The changing panorama of cerebral palsy in Sweden. IX. Prevalence and origin in the birth-year period 1995–1998. *Acta Paediatr.* 94 287–294. 10.1111/j.1651-2227.2005.tb03071.x 16028646

[B267] Himmelmann K. Hagberg G. Wiklund L. M. Eek M. N. Uvebrant P. (2007). Dyskinetic cerebral palsy: a population-based study of children born between 1991 and 1998. *Dev. Med. Child Neurol.* 49 246–251. 10.1111/j.1469-8749.2007.00246.x 17376133

[B268] Hisanaga K. Onodera H. Kogure K. (1986). Changes in levels of purine and pyrimidine nucleotides during acute hypoxia and recovery in neonatal rat brain. *J. Neurochem.* 47 1344–1350. 10.1111/j.1471-4159.1986.tb00763.x 3020172

[B269] Hobbs C. Thoresen M. Tucker A. Aquilina K. Chakkarapani E. Dingley J. (2008). Xenon and hypothermia combine additively, offering long-term functional and histopathologic neuroprotection after neonatal hypoxia/ischemia. *Stroke* 39 1307–1313. 10.1161/STROKEAHA.107.499822 18309163

[B270] Hoerder-Suabedissen A. Molnár Z. (2012). Morphology of mouse subplate cells with identified projection targets changes with age. *J. Comp. Neurol.* 520 174–185. 10.1002/cne.22725 21800308

[B271] Hoerder-Suabedissen A. Molnár Z. (2015). Development, evolution and pathology of neocortical subplate neurons. *Nat. Rev. Neurosci.* 16 133–146. 10.1038/nrn3915 25697157

[B272] Hoerder-Suabedissen A. Oeschger F. M. Krishnan M. L. Belgard T. G. Wang W. Z. Lee S. (2013). Expression profiling of mouse subplate reveals a dynamic gene network and disease association with autism and schizophrenia. *Proc. Natl. Acad. Sci. U.S.A.* 110 3555–3560. 10.1073/pnas.1218510110 23401504 PMC3587197

[B273] Hoerder-Suabedissen A. Wang W. Z. Lee S. Davies K. E. Goffinet A. M. Rakic S. (2009). Novel markers reveal subpopulations of subplate neurons in the murine cerebral cortex. *Cereb. Cortex* 19 1738–1750. 10.1093/cercor/bhn195 19008461

[B274] Holmes G. L. (1991). The long-term effects of seizures on the developing brain: clinical and laboratory issues. *Brain Dev.* 13 393–409. 10.1016/S0387-7604(12)80037-4 1810153

[B275] Holmes G. L. Ben-Ari Y. (2001). The neurobiology and consequences of epilepsy in the developing brain. *Pediatr. Res.* 49 320–325. 10.1203/00006450-200103000-00004 11228256

[B276] Horn E. P. Bein B. Broch O. Iden T. Bohm R. Latz S. K. (2016). Warming before and after epidural block before general anaesthesia for major abdominal surgery prevents perioperative hypothermia: a randomised controlled trial. *Eur. J. Anaesthesiol.* 33 334–340. 10.1097/EJA.0000000000000369 26555870

[B277] Hou S. T. Jiang S. X. Huang D. Desbois A. (2007). A novel adenoviral vector-mediated neuronal selective gene expression in neonatal mouse brain in response to hypoxia. *Neurosci. Lett.* 419 23–27. 10.1016/j.neulet.2007.03.059 17418946

[B278] Hou X. Ding H. Teng Y. Zhou C. Tang X. Li S. (2007). Research on the relationship between brain anoxia at different regional oxygen saturations and brain damage using near-infrared spectroscopy. *Physiol. Meas.* 28 1251–1265. 10.1088/0967-3334/28/10/010 17906392

[B279] Hou X. L. Ding H. Y. Zhou C. L. Tang X. Y. Ding H. S. Teng Y. C. (2007). Correlation of brain hypoxia at different degrees with brain function and brain damage investigated using near infrared spectroscopy. *Zhonghua Er Ke Za Zhi* 45 523–528. 17953810

[B280] Hu B. R. Liu C. L. Ouyang Y. Blomgren K. Siesjo B. K. (2000). Involvement of caspase-3 in cell death after hypoxia-ischemia declines during brain maturation. *J. Cerebral Blood Flow Metab.* 20 1294–1300. 10.1097/00004647-200009000-00003 10994850

[B281] Hu C. J. Wang L. Y. Chodosh L. A. Keith B. Simon M. C. (2003). Differential roles of hypoxia-inducible factor 1alpha (HIF-1alpha) and HIF-2alpha in hypoxic gene regulation. *Mol. Cell. Biol.* 23 9361–9374. 10.1128/MCB.23.24.9361-9374.2003 14645546 PMC309606

[B282] Hu X. Qiu J. Grafe M. R. Rea H. C. Rassin D. K. Perez-Polo J. R. (2003). Bcl-2 family members make different contributions to cell death in hypoxia and/or hyperoxia in rat cerebral cortex. *Int. J. Dev. Neurosci.* 21 371–377. 10.1016/S0736-5748(03)00089-3 14599483

[B283] Hunt R. W. Neil J. J. Coleman L. T. Kean M. J. Inder T. E. (2004). Apparent diffusion coefficient in the posterior limb of the internal capsule predicts outcome after perinatal asphyxia. *Pediatrics* 114 999–1003. 10.1542/peds.2003-0935-L 15466097

[B284] Hurtley S. M. Helenius A. (1989). Protein oligomerization in the endoplasmic reticulum. *Annu. Rev. Cell Biol.* 5 277–307. 10.1146/annurev.cb.05.110189.001425 2688707

[B285] Hutton L. C. Abbass M. Dickinson H. Ireland Z. Walker D. W. (2009). Neuroprotective properties of melatonin in a model of birth asphyxia in the spiny mouse (*Acomys cahirinus*). *Dev. Neurosci.* 31 437–451. 10.1159/000232562 19684403

[B286] Ikeda K. Tanihara H. Honda Y. Tatsuno T. Noguchi H. Nakayama C. (1999). BDNF attenuates retinal cell death caused by chemically induced hypoxia in rats. *Invest. Ophthalmol. Vis. Sci.* 40 2130–2140. 10440270

[B287] Ikeda T. Mishima K. Yoshikawa T. Iwasaki K. Fujiwara M. Xia Y. X. (2001). Selective and long-term learning impairment following neonatal hypoxic-ischemic brain insult in rats. *Behav. Brain Res.* 118 17–25. 10.1016/S0166-4328(00)00287-4 11163630

[B288] Ikonomidou C. Bosch F. Miksa M. Bittigau P. Vockler J. Dikranian K. (1999). Blockade of NMDA receptors and apoptotic neurodegeneration in the developing brain. *Science* 283 70–74. 10.1126/science.283.5398.70 9872743

[B289] Ikonomidou C. Kaindl A. M. (2011). Neuronal death and oxidative stress in the developing brain. *Antioxid. Redox Signal.* 14 1535–1550. 10.1089/ars.2010.3581 20919934

[B290] Im S. Yu J. Park E. Lee J. Kim H. Park K. (2010). Induction of striatal neurogenesis enhances functional recovery in an adult animal model of neonatal hypoxic-ischemic brain injury. *Neuroscience* 169 259–268. 10.1016/j.neuroscience.2010.04.038 20610036

[B291] Inder T. Neil J. Kroenke C. Dieni S. Yoder B. Rees S. (2005a). Investigation of cerebral development and injury in the prematurely born primate by magnetic resonance imaging and histopathology. *Dev. Neurosci.* 27 100–111. 16046843 10.1159/000085981

[B292] Inder T. Neil J. Yoder B. Rees S. (2005b). Patterns of cerebral injury in a primate model of preterm birth and neonatal intensive care. *J. Child Neurol.* 20 965–967. 16417843 10.1177/08830738050200120601

[B293] Inder T. E. Hunt R. W. Morley C. J. Coleman L. Stewart M. Doyle L. W. (2004). Randomized trial of systemic hypothermia selectively protects the cortex on MRI in term hypoxic-ischemic encephalopathy. *J. Pediatr.* 145 835–837. 10.1016/j.jpeds.2004.07.034 15580212

[B294] Inder T. E. Huppi P. S. Warfield S. Kikinis R. Zientara G. P. Barnes P. D. (1999). Periventricular white matter injury in the premature infant is followed by reduced cerebral cortical gray matter volume at term. *Ann. Neurol.* 46 755–760. 10.1002/1531-8249(199911)46:5<755::AID-ANA11>3.0.CO;2-0 10553993

[B295] Inder T. E. Volpe J. J. (2000). Mechanisms of perinatal brain injury. *Semin. Neonatol.* 5 3–16. 10.1053/siny.1999.0112 10802746

[B296] Ireland Z. Dickinson H. Fleiss B. Hutton L. C. Walker D. W. (2010). Behavioural effects of near-term acute fetal hypoxia in a small precocial animal, the spiny mouse (*Acomys cahirinus*). *Neonatology* 97 45–51. 10.1159/000227293 19590246

[B297] Ireland Z. Dickinson H. Snow R. Walker D. W. (2008). Maternal creatine: does it reach the fetus and improve survival after an acute hypoxic episode in the spiny mouse (*Acomys cahirinus*)? *Am. J. Obstet. Gynecol.* 198 431 e1–6. 10.1016/j.ajog.2007.10.790 18295173

[B298] Ireland Z. Russell A. P. Wallimann T. Walker D. W. Snow R. (2009). Developmental changes in the expression of creatine synthesizing enzymes and creatine transporter in a precocial rodent, the spiny mouse. *BMC Dev. Biol.* 9:39 10.1186/1471-213X-9-39 PMC2713216 19570237

[B299] Ishigami S. Sandkvist M. Tsui F. Moore E. Coleman T. A. Lawrence D. A. (2007). Identification of a novel targeting sequence for regulated secretion in the serine protease inhibitor neuroserpin. *Biochem. J.* 402 25–34. 10.1042/BJ20061170 17040209 PMC1783992

[B300] Iuvone L. Geloso M. C. Dell’Anna E. (1996). Changes in open field behavior, spatial memory, and hippocampal parvalbumin immunoreactivity following enrichment in rats exposed to neonatal anoxia. *Exp. Neurol.* 139 25–33. 10.1006/exnr.1996.0077 8635565

[B301] Iyer N. V. Leung S. W. Semenza G. L. (1998). The human hypoxia-inducible factor 1alpha gene: HIF1A structure and evolutionary conservation. *Genomics* 52 159–165. 10.1006/geno.1998.5416 9782081

[B302] Jabaudon D. Scanziani M. Gahwiler B. H. Gerber U. (2000). Acute decrease in net glutamate uptake during energy deprivation. *Proc. Natl. Acad. Sci. U.S.A.* 97 5610–5615. 10.1073/pnas.97.10.5610 10805815 PMC25876

[B303] Jacobs S. Hunt R. Tarnow-Mordi W. Inder T. Davis P. (2005). 181 cooling for newborns with hypoxic ischaemic encephalopathy. *Pediatr. Res.* 58 385–385. 10.1016/j.arcped.2012.11.007

[B304] Jacobs S. E. Berg M. Hunt R. Tarnow-Mordi W. O. Inder T. E. Davis P. G. (2013). Cooling for newborns with hypoxic ischaemic encephalopathy. *Cochrane Database Syst. Rev.* 4 CD003311 10.1002/14651858.CD003311.pub3 14583966

[B305] Jacovina A. T. Zhong F. Khazanova E. Lev E. Deora A. B. Hajjar K. A. (2001). Neuritogenesis and the nerve growth factor-induced differentiation of PC-12 cells requires annexin II-mediated plasmin generation. *J. Biol. Chem.* 276 49350–49358. 10.1074/jbc.M106289200 11679580

[B306] Jansen E. M. Low W. C. (1996a). Long-term effects of neonatal ischemic-hypoxic brain injury on sensorimotor and locomotor tasks in rats. *Behav. Brain Res.* 78 189–194. 8864051 10.1016/0166-4328(95)00248-0

[B307] Jansen E. M. Low W. C. (1996b). Quantitative analysis of contralateral hemisphere hypertrophy and sensorimotor performance in adult rats following unilateral neonatal ischemic-hypoxic brain injury. *Brain Res.* 708 93–99. 8720863 10.1016/0006-8993(95)01288-5

[B308] Jansen E. M. Solberg L. Underhill S. Wilson S. Cozzari C. Hartman B. K. (1997). Transplantation of fetal neocortex ameliorates sensorimotor and locomotor deficits following neonatal ischemic–hypoxic brain injury in rats. *Exp. Neurol.* 147 487–497. 10.1006/exnr.1997.6596 9344572

[B309] Jantzie L. L. Corbett C. J. Berglass J. Firl D. J. Flores J. Mannix R. (2014). Complex pattern of interaction between in utero hypoxia-ischemia and intra-amniotic inflammation disrupts brain development and motor function. *J. Neuroinflammation* 11:131 10.1186/1742-2094-11-131 PMC4128546 25082427

[B310] Jantzie L. L. Corbett C. J. Firl D. J. Robinson S. (2015a). Postnatal erythropoietin mitigates impaired cerebral cortical development following subplate loss from prenatal hypoxia-ischemia. *Cereb.Cortex* 25 2683–2695. 10.1093/cercor/bhu066 24722771 PMC4537428

[B311] Jantzie L. L. Hu M. Y. Park H. K. Jackson M. C. Yu J. Maxwell J. R. (2015b). Chloride cotransporter NKCC1 inhibitor bumetanide protects against white matter injury in a rodent model of periventricular leukomalacia. *Pediatr. Res.* 77 554–562. 10.1038/pr.2015.9 25585037

[B312] Jantzie L. L. Winer J. L. Maxwell J. R. Chan L. A. Robinson S. (2015c). Modeling encephalopathy of prematurity using prenatal Hypoxia-ischemia with Intra-amniotic lipopolysaccharide in rats. *J. Vis. Exp.* 105:e53196 10.3791/53196 PMC4692750 26649874

[B313] Jarvis S. Glinianaia S. V. Arnaud C. Fauconnier J. Johnson A. McManus V. (2005). Case gender and severity in cerebral palsy varies with intrauterine growth. *Arch. Dis. Child.* 90 474–479. 10.1136/adc.2004.052670 15851428 PMC1720399

[B314] Jatana M. Singh I. Singh A. K. Jenkins D. (2006). Combination of systemic hypothermia and N-acetylcysteine attenuates hypoxic-ischemic brain injury in neonatal rats. *Pediatr. Res.* 59 684–689. 10.1203/01.pdr.0000215045.91122.44 16627882

[B315] Jelinski S. E. Yager J. Y. Juurlink B. H. (1999). Preferential injury of oligodendroblasts by a short hypoxic–ischemic insult. *Brain Res.* 815 150–153. 10.1016/S0006-8993(98)01053-1 9974135

[B316] Jendelová P. Herynek V. Urdzikova L. Glogarová K. Kroupová J. Andersson B. (2004). Magnetic resonance tracking of transplanted bone marrow and embryonic stem cells labeled by iron oxide nanoparticles in rat brain and spinal cord. *J. Neurosci. Res.* 76 232–243. 10.1002/jnr.20041 15048921

[B317] Jensen A. Garnier Y. Berger R. (1999). Dynamics of fetal circulatory responses to hypoxia and asphyxia. *Eur. J. Obstet. Gynecol. Reprod. Biol.* 84 155–172. 10.1016/S0301-2115(98)00325-X 10428339

[B318] Jensen F. (1995). An animal model of hypoxia-induced perinatal seizures. *Ital. J. Neurol. Sci.* 16 59–68. 10.1007/BF02229075 7642353

[B319] Jensen F. Blume H. Alvarado S. Firkusny I. Geary C. (1995). NBQX blocks acute and late epileptogenic effects of perinatal hypoxia. *Epilepsia* 36 966–972. 10.1111/j.1528-1157.1995.tb00954.x 7555960

[B320] Johnston M. V. (2001). Excitotoxicity in neonatal hypoxia. *Ment. Retard. Dev. Disabil. Res. Rev.* 7 229–234. 10.1002/mrdd.1032 11754516

[B321] Johnston M. V. (2005). Excitotoxicity in perinatal brain injury. *Brain Pathol.* 15 234–240. 10.1111/j.1750-3639.2005.tb00526.x 16196390 PMC8095755

[B322] Johnston M. V. (2009). Plasticity in the developing brain: implications for rehabilitation. *Dev. Disab. Res. Rev.* 15 94–101. 10.1002/ddrr.64 19489084

[B323] Johnston M. V. Fatemi A. Wilson M. A. Northington F. (2011). Treatment advances in neonatal neuroprotection and neurointensive care. *Lancet Neurol.* 10 372–382. 10.1016/S1474-4422(11)70016-3 21435600 PMC3757153

[B324] Johnston M. V. Hagberg H. (2007). Sex and the pathogenesis of cerebral palsy. *Dev. Med. Child Neurol.* 49 74–78. 10.1017/S0012162207000199.x 17209983

[B325] Jones M. K. Szabo I. L. Kawanaka H. Husain S. S. Tarnawski A. S. (2002). von Hippel Lindau tumor suppressor and HIF-1alpha: new targets of NSAIDs inhibition of hypoxia-induced angiogenesis. *FASEB J.* 16 264–266. 11772947 10.1096/fj.01-0589fje

[B326] Joseph B. Nandhu M. S. Paulose C. S. (2010). Dopamine D1 and D2 receptor functional down regulation in the cerebellum of hypoxic neonatal rats: neuroprotective role of glucose and oxygen, epinephrine resuscitation. *Pharmacol. Res.* 61 136–141. 10.1016/j.phrs.2009.08.007 19720148

[B327] Judas M. Sedmak G. Pletikos M. Jovanov-Milosevic N. (2010). Populations of subplate and interstitial neurons in fetal and adult human telencephalon. *J. Anat.* 217 381–399. 10.1111/j.1469-7580.2010.01284.x 20979586 PMC2992415

[B328] Juul S. E. Beyer R. P. Bammler T. K. McPherson R. J. Wilkerson J. Farin F. M. (2009). Microarray analysis of high-dose recombinant erythropoietin treatment of unilateral brain injury in neonatal mouse hippocampus. *Pediatr. Res.* 65 485–492. 10.1203/PDR.0b013e31819d90c8 19190543

[B329] Kali G. T. Martinez-Biarge M. Van Zyl J. Smith J. Rutherford M. (2015). Management of therapeutic hypothermia for neonatal hypoxic ischaemic encephalopathy in a tertiary centre in South Africa. *Arch. Dis. Child. Fetal Neonatal. Ed.* 100 F519–F523. 10.1136/archdischild-2015-308398 26126846

[B330] Kaneko K. (1985a). The effect of perinatal anoxia on amino acid metabolism in the developing brain. Part I: the effect of experimental anoxia on the free amino acid patterns in the brain of neonatal rats. *Brain Dev.* 7 392–399. 4061775 10.1016/s0387-7604(85)80136-4

[B331] Kaneko K. (1985b). The effect of perinatal anoxia on amino acid metabolism in the developing brain. Part II: the effect of perinatal anoxia on the free amino acid patterns in CSF of infants and children. *Brain Dev.* 7 400–407. 4061776 10.1016/s0387-7604(85)80137-6

[B332] Kaneko T. Sawada T. Kinugawa H. Kuriyama Y. Naritomi H. (1985). A case of inverse ocular bobbing associated with cluster respiration in hypoxic encephalopathy. *Rinsho shinkeigaku* 25 1192–1195. 4092394

[B333] Kanold P. O. (2009). Subplate neurons: crucial regulators of cortical development and plasticity. *Front. Neuroanat.* 3:16 10.3389/neuro.05.016.2009 PMC2737439 19738926

[B334] Kanold P. O. Kara P. Reid R. C. Shatz C. J. (2003). Role of subplate neurons in functional maturation of visual cortical columns. *Science* 301 521–525. 10.1126/science.1084152 12881571

[B335] Kanold P. O. Luhmann H. J. (2010). The subplate and early cortical circuits. *Annu. Rev. Neurosci.* 33 23–48. 10.1146/annurev-neuro-060909-153244 20201645

[B336] Kanold P. O. Shatz C. J. (2006). Subplate neurons regulate maturation of cortical inhibition and outcome of ocular dominance plasticity. *Neuron* 51 627–638. 10.1016/j.neuron.2006.07.008 16950160

[B337] Karalis F. Soubasi V. Georgiou T. Nakas C. T. Simeonidou C. Guiba-Tziampiri O. (2011). Resveratrol ameliorates hypoxia/ischemia-induced behavioral deficits and brain injury in the neonatal rat brain. *Brain Res.* 1425 98–110. 10.1016/j.brainres.2011.09.044 22018692

[B338] Kattwinkel J. Perlman J. M. Aziz K. Colby C. Fairchild K. Gallagher J. (2010). Part 15: neonatal resuscitation: 2010 American heart association guidelines for cardiopulmonary resuscitation and emergency cardiovascular care. *Circulation* 122(18 Suppl. 3), S909–S919. 10.1161/CIRCULATIONAHA.110.971119 20956231

[B339] Kaufman S. A. Miller S. P. Ferriero D. M. Glidden D. H. Barkovich A. J. Partridge J. C. (2003). Encephalopathy as a predictor of magnetic resonance imaging abnormalities in asphyxiated newborns. *Pediatr. Neurol.* 28 342–346. 10.1016/S0887-8994(03)00015-8 12878294

[B340] Kaur C. Dheen S. T. Ling E. A. (2007). From blood to brain: amoeboid microglial cell, a nascent macrophage and its functions in developing brain. *Acta Pharmacol. Sin.* 28 1087–1096. 10.1111/j.1745-7254.2007.00625.x 17640468

[B341] Kaur C. Ling E. A. (2009). Periventricular white matter damage in the hypoxic neonatal brain: role of microglial cells. *Prog. Neurobiol.* 87 264–280. 10.1016/j.pneurobio.2009.01.003 19428957

[B342] Kaur C. Rathnasamy G. Ling E. (2013). Roles of activated microglia in hypoxia induced neuroinflammation in the developing brain and the retina. *J. Neuroimmune Pharmacol.* 8 66–78. 10.1007/s11481-012-9347-2 22367679

[B343] Kemp G. J. Radda G. K. (1994). Quantitative interpretation of bioenergetic data from 31P and 1H magnetic resonance spectroscopic studies of skeletal muscle: an analytical review. *Magn. Reson. Q.* 10 43–63. 8161485

[B344] Kida H. Nomura S. Shinoyama M. Ideguchi M. Owada Y. Suzuki M. (2013). The effect of hypothermia therapy on cortical laminar disruption following ischemic injury in neonatal mice. *PLoS ONE* 8:e68877 10.1371/journal.pone.0068877 PMC3720877 23894362

[B345] Kim G. S. Cho S. Nelson J. W. Zipfel G. J. Han B. H. (2014). TrkB agonist antibody pretreatment enhances neuronal survival and long-term sensory motor function following hypoxic ischemic injury in neonatal rats. *PLoS ONE* 9:e88962 10.1371/journal.pone.0088962 PMC3925177 24551199

[B346] Kinney H. C. Haynes R. L. Xu G. Andiman S. E. Folkerth R. D. Sleeper L. A. (2012). Neuron deficit in the white matter and subplate in periventricular leukomalacia. *Ann. Neurol.* 71 397–406. 10.1002/ana.22612 22451205 PMC3315053

[B347] Kletkiewicz H. Nowakowska A. Siejka A. Mila-Kierzenkowska C. Wozniak A. Caputa M. (2016). Deferoxamine prevents cerebral glutathione and vitamin E depletions in asphyxiated neonatal rats: role of body temperature. *Int. J. Hyperthermia* 32 211–220. 10.3109/02656736.2015.1125955 26794834

[B348] Klionsky D. J. Emr S. D. (2000). Autophagy as a regulated pathway of cellular degradation. *Science* 290 1717–1721. 10.1126/science.290.5497.1717 11099404 PMC2732363

[B349] Kniesel U. Risau W. Wolburg H. (1996). Development of blood-brain barrier tight junctions in the rat cortex. *Brain Res. Dev. Brain Res.* 96 229–240. 10.1016/0165-3806(96)00117-4 8922685

[B350] Koh S. Jensen F. E. (2001). Topiramate blocks perinatal hypoxia-induced seizures in rat pups. *Ann. Neurol.* 50 366–372. 10.1002/ana.1122 11558793

[B351] Koike M. Shibata M. Tadakoshi M. Gotoh K. Komatsu M. Waguri S. (2008). Inhibition of autophagy prevents hippocampal pyramidal neuron death after hypoxic-ischemic injury. *Am. J. Pathol.* 172 454–469. 10.2353/ajpath.2008.070876 18187572 PMC2312361

[B352] Kondo S. Al-Hasani H. Hoerder-Suabedissen A. Wang W. Z. Molnár Z. (2015). Secretory function in subplate neurons during cortical development. *Front. Neurosci.* 9:100 10.3389/fnins.2015.00100 PMC4374456 25859180

[B353] Kondo S. Hino S. I. Saito A. Kanemoto S. Kawasaki N. Asada R. (2012). Activation of OASIS family, ER stress transducers, is dependent on its stabilization. *Cell Death Differ.* 19 1939–1949. 10.1038/cdd.2012.77 22705851 PMC3504707

[B354] Kondo S. Murakami T. Tatsumi K. Ogata M. Kanemoto S. Otori K. (2005). OASIS, a CREB/ATF-family member, modulates UPR signalling in astrocytes. *Nat. Cell Biol.* 7 186–194. 10.1038/ncb1213 15665855

[B355] Kondo S. Saito A. Asada R. Kanemoto S. Imaizumi K. (2011). Physiological unfolded protein response regulated by OASIS family members, transmembrane bZIP transcription factors. *IUBMB Life* 63 233–239. 10.1002/iub.433 21438114

[B356] Kostovic I. Jovanov-Milosevic N. Rados M. Sedmak G. Benjak V. Kostovic-Srzentic M. (2014). Perinatal and early postnatal reorganization of the subplate and related cellular compartments in the human cerebral wall as revealed by histological and MRI approaches. *Brain Struct. Funct.* 219 231–253. 10.1007/s00429-012-0496-0 23250390

[B357] Kostovic I. Judas M. (2006). Prolonged coexistence of transient and permanent circuitry elements in the developing cerebral cortex of fetuses and preterm infants. *Dev. Med. Child Neurol.* 48 388–393. 10.1017/S0012162206000831 16608549

[B358] Kostovic I. Judas M. (2010). The development of the subplate and thalamocortical connections in the human foetal brain. *Acta Paediatr.* 99 1119–1127. 10.1111/j.1651-2227.2010.01811.x 20367617

[B359] Kostovic I. Judas M. Rados M. Hrabac P. (2002). Laminar organization of the human fetal cerebrum revealed by histochemical markers and magnetic resonance imaging. *Cereb. Cortex* 12 536–544. 10.1093/cercor/12.5.536 11950771

[B360] Kostovic I. Judas M. Sedmak G. (2011). Developmental history of the subplate zone, subplate neurons and interstitial white matter neurons: relevance for schizophrenia. *Int. J. Dev. Neurosci.* 29 193–205. 10.1016/j.ijdevneu.2010.09.005 20883772

[B361] Kostovic I. Rakic P. (1990). Developmental history of the transient subplate zone in the visual and somatosensory cortex of the macaque monkey and human brain. *J. Comp. Neurol.* 297 441–470. 10.1002/cne.902970309 2398142

[B362] Kracer B. Hintz S. R. Van Meurs K. P. Lee H. C. (2014). Hypothermia therapy for neonatal hypoxic ischemic encephalopathy in the state of California. *J. Pediatr.* 165 267–273. 10.1016/j.jpeds.2014.04.052 24929331 PMC4111956

[B363] Krajnc D. Norton H. N. Hadjiconstantinou M. (1996). Glutamate, glutamine and glutamine synthetase in the neonatal rat brain following hypoxia. *Brain Res.* 707 134–137. 10.1016/0006-8993(95)01372-5 8866724

[B364] Krajnc D. Wemlinger T. A. Neff N. H. Hadjiconstantinou M. (1994). Neonatal hypoxia: early neurotransmitter responses and the consequences of treatment with GM1 ganglioside. *J. Pharmacol. Exp. Ther.* 271 1299–1305. 7996438

[B365] Krueger S. R. Ghisu G. P. Cinelli P. Gschwend T. P. Osterwalder T. Wolfer D. P. (1997). Expression of neuroserpin, an inhibitor of tissue plasminogen activator, in the developing and adult nervous system of the mouse. *J. Neurosci.* 17 8984–8996. 9364046 10.1523/JNEUROSCI.17-23-08984.1997 PMC6573583

[B366] Krystosek A. Seeds N. W. (1981a). Plasminogen activator release at the neuronal growth cone. *Science* 213 1532–1534. 7197054 10.1126/science.7197054

[B367] Krystosek A. Seeds N. W. (1981b). Plasminogen activator secretion by granule neurons in cultures of developing cerebellum. *Proc. Natl. Acad. Sci. U.S.A.* 78 7810–7814. 6950420 10.1073/pnas.78.12.7810 PMC349361

[B368] Kuan C. Lawrence D. A. Yang D. E. Adhami F. (2010). Plasminogen Activator Inhibitor Amelioration of Newborn Hypoxic Ischemic Brain Injury. US 20100286053.

[B369] L’Abee C. de Vries L. S. van der Grond J. Groenendaal F. (2005). Early diffusion-weighted MRI and 1H-magnetic resonance spectroscopy in asphyxiated full-term neonates. *Biol. Neonate* 88 306–312. 10.1159/000087628 16113525

[B370] Lafemina M. J. Sheldon R. A. Ferriero D. M. (2006). Acute hypoxia-ischemia results in hydrogen peroxide accumulation in neonatal but not adult mouse brain. *Pediatr. Res.* 59 680–683. 10.1203/01.pdr.0000214891.35363.6a 16627881

[B371] Lai M. C. Yang S. N. (2011). Perinatal hypoxic-ischemic encephalopathy. *J. Biomed. Biotechnol.* 2011:609813 10.1155/2011/609813 PMC3010686 21197402

[B372] Lam J. S. Anderson E. M. Hao Y. (2014). LPS quantitation procedures. *Methods Mol. Biol.* 1149 375–402. 10.1007/978-1-4939-0473-0_31 24818921

[B373] Laptook A. R. Shankaran S. Ambalavanan N. Carlo W. A. McDonald S. A. Higgins R. D. (2009). Outcome of term infants using apgar scores at 10 minutes following hypoxic-ischemic encephalopathy. *Pediatrics* 124 1619–1626. 10.1542/peds.2009-0934 19948631 PMC2821195

[B374] Laroia N. McBride L. Baggs R. Guillet R. (1997). Dextromethorphan ameliorates effects of neonatal hypoxia on brain morphology and seizure threshold in rats. *Dev. Brain Res.* 100 29–34. 10.1016/S0165-3806(97)00018-7 9174243

[B375] Larroche J. C. (1977). *Developmental Pathology of the Neonate.* Amsterdam: Excerpta Medica.

[B376] Larroque B. Breart G. Kaminski M. Dehan M. Andre M. Burguet A. (2004). Survival of very preterm infants: epipage, a population based cohort study. *Arch. Dis. Child. Fetal Neonatal Ed.* 89 F139–F144. 10.1136/adc.2002.020396 14977898 PMC1756022

[B377] Lawn J. E. Cousens S. Zupan J. Lancet Neonatal Survival Steering Team. (2005). 4 million neonatal deaths: when? Where? Why? *Lancet* 365 891–900. 15752534 10.1016/S0140-6736(05)71048-5

[B378] Lebeurrier N. Liot G. Lopez-Atalaya J. Fernandez-Monreal M. Sonderegger P. Vivien D. (2005a). Neuroprotective activity of neuroserpin against NMDA receptor-mediated excitotoxicity. *J. Cereb. Blood Flow Metab.* 25 S455.

[B379] Lebeurrier N. Liot G. Lopez-Atalaya J. P. Orset C. Fernandez-Monreal M. Sonderegger P. (2005b). The brain-specific tissue-type plasminogen activator inhibitor, neuroserpin, protects neurons against excitotoxicity both in vitro and in vivo. *Mol. Cell. Neurosci.* 30 552–558. 16209928 10.1016/j.mcn.2005.09.005

[B380] Lee A. C. Kozuki N. Blencowe H. Vos T. Bahalim A. Darmstadt G. L. (2013). Intrapartum-related neonatal encephalopathy incidence and impairment at regional and global levels for 2010 with trends from 1990. *Pediatr. Res.* 74(Suppl. 1), 50–72. 10.1038/pr.2013.206 24366463 PMC3873711

[B381] Lee A. S. (1987). Coordinated regulation of a set of genes by glucose and calcium ionophores in mammalian cells. *Trends Biochem. Sci.* 12 20–23. 10.1016/0968-0004(87)90011-9

[B382] Lee A. S. (2001). The glucose-regulated proteins: stress induction and clinical applications. *Trends Biochem. Sci.* 26 504–510. 10.1016/S0968-0004(01)01908-9 11504627

[B383] Lee A. S. (2005). The ER chaperone and signaling regulator GRP78/BiP as a monitor of endoplasmic reticulum stress. *Methods* 35 373–381. 10.1016/j.ymeth.2004.10.010 15804610

[B384] Lee B. S. Woo C. Kim S. Kim K. (2010). Long-term neuroprotective effect of postischemic hypothermia in a neonatal rat model of severe hypoxic ischemic encephalopathy: a comparative study on the duration and depth of hypothermia. *Pediatr. Res.* 68 303–308. 10.1203/00006450-201011001-00592 20606598

[B385] Lee T. W. Coates L. C. Birch N. P. (2008). Neuroserpin regulates N-cadherin-mediated cell adhesion independently of its activity as an inhibitor of tissue plasminogen activator. *J. Neurosci. Res.* 86 1243–1253. 10.1002/jnr.21592 18092357

[B386] Lee T. W. Tsang V. W. Birch N. P. (2015a). Physiological and pathological roles of tissue plasminogen activator and its inhibitor neuroserpin in the nervous system. *Front. Cell. Neurosci.* 9:396 10.3389/fncel.2015.00396 PMC4602146 26528129

[B387] Lee T. W. Yang A. S. Brittain T. Birch N. P. (2015b). An analysis approach to identify specific functional sites in orthologous proteins using sequence and structural information: application to neuroserpin reveals regions that differentially regulate inhibitory activity. *Proteins* 83 135–152. 10.1002/prot.24711 25363759

[B388] Legido A. Clancy R. R. Berman P. H. (1991). Neurologic outcome after electroencephalographically proven neonatal seizures. *Pediatrics* 88 583–596. 1881741

[B389] Lehnardt S. Massillon L. Follett P. Jensen F. E. Ratan R. Rosenberg P. A. (2003). Activation of innate immunity in the CNS triggers neurodegeneration through a Toll-like receptor 4-dependent pathway. *Proc. Natl. Acad. Sci. U.S.A.* 100 8514–8519. 10.1073/pnas.1432609100 12824464 PMC166260

[B390] Lein E. S. Finney E. M. McQuillen P. S. Shatz C. J. (1999). Subplate neuron ablation alters neurotrophin expression and ocular dominance column formation. *Proc. Natl. Acad. Sci. U.S.A.* 96 13491–13495. 10.1073/pnas.96.23.13491 10557348 PMC23975

[B391] Leonardo C. C. Pennypacker K. R. (2009). Neuroinflammation and MMPs: potential therapeutic targets in neonatal hypoxic-ischemic injury. *J. Neuroinflammation* 6:13 10.1186/1742-2094-6-13 PMC2674036 19368723

[B392] Levene M. Grindulis H. Sands C. Moore J. (1986). Comparison of two methods of predicting outcome in perinatal asphyxia. *Lancet* 327 67–69. 10.1016/S0140-6736(86)90718-X 2867316

[B393] Leviton A. Gressens P. (2007). Neuronal damage accompanies perinatal white-matter damage. *Trends Neurosci.* 30 473–478. 10.1016/j.tins.2007.05.009 17765331

[B394] Leviton A. Paneth N. Reuss M. L. Susser M. Allred E. N. Dammann O. (1999). Maternal infection, fetal inflammatory response, and brain damage in very low birth weight infants. *Dev. Epidemiol. Network Invest. Pediatr. Res.* 46 566–575. 10.1203/00006450-199911000-00013 10541320

[B395] Li A. M. Chau V. Poskitt K. J. Sargent M. A. Lupton B. A. Hill A. (2009). White matter injury in term newborns with neonatal encephalopathy. *Pediatr. Res.* 61 85–89. 10.1203/PDR.0b013e31818912d2 18787422

[B396] Li C. Jackson R. M. (2002). Reactive species mechanisms of cellular hypoxia-reoxygenation injury. *Am. J. Physiol. Cell Physiol.* 282 C227–C241. 10.1152/ajpcell.00112.2001 11788333

[B397] Li M. Yu A. Zhang F. Dai G. Cheng H. Wang X. (2012). Treatment of one case of cerebral palsy combined with posterior visual pathway injury using autologous bone marrow mesenchymal stem cells. *J. Trans. Med.* 10:100 10.1186/1479-5876-10-100 PMC3479002 22607263

[B398] Li P. Nijhawan D. Budihardjo I. Srinivasula S. M. Ahmad M. Alnemri E. S. (1997). Cytochrome c and dATP-dependent formation of Apaf-1/caspase-9 complex initiates an apoptotic protease cascade. *Cell* 91 479–489. 10.1016/S0092-8674(00)80434-1 9390557

[B399] Liao Y. Cotten M. Tan S. Kurtzberg J. Cairo M. S. (2013). Rescuing the neonatal brain from hypoxic injury with autologous cord blood. *Bone Marrow Transplant.* 48 890–900. 10.1038/bmt.2012.169 22964590

[B400] Liauw L. van Wezel-Meijler G. Veen S. van Buchem M. A. van der Grond J. (2009). Do apparent diffusion coefficient measurements predict outcome in children with neonatal hypoxic-ischemic encephalopathy? *AJNR Am. J. Neuroradiol.* 30 264–270. 10.3174/ajnr.A1318 18842756 PMC7051373

[B401] Lieblein-Boff J. C. McKim D. B. Shea D. T. Wei P. Deng Z. Sawicki C. (2013). Neonatal *E. coli* infection causes neuro-behavioral deficits associated with hypomyelination and neuronal sequestration of iron. *J. Neurosci.* 33 16334–16345. 10.1523/JNEUROSCI.0708-13.2013 24107964 PMC3792468

[B402] Lin E. P. Miles L. Hughes E. A. McCann J. C. Vorhees C. V. McAuliffe J. J. (2014). A combination of mild hypothermia and sevoflurane affords long-term protection in a modified neonatal mouse model of cerebral hypoxia-ischemia. *Anesth. Analg.* 119 1158–1173. 10.1213/ANE.0000000000000262 24878681

[B403] Liu Y. Silverstein F. S. Skoff R. Barks J. D. (2002). Hypoxic-ischemic oligodendroglial injury in neonatal rat brain. *Pediatr. Res.* 51 25–33. 10.1203/00006450-200201000-00007 11756636

[B404] Lochner J. E. Honigman L. S. Grant W. F. Gessford S. K. Hansen A. B. Silverman M. A. (2006). Activity-dependent release of tissue plasminogen activator from the dendritic spines of hippocampal neurons revealed by live-cell imaging. *J. Neurobiol.* 66 564–577. 10.1002/neu.20250 16555239

[B405] Lockshin R. A. Zakeri Z. (1994). Programmed cell death: early changes in metamorphosing cells. *Biochem. Cell Biol.* 72 589–596. 10.1139/o94-078 7654332

[B406] Lodish H. F. (1988). Transport of secretory and membrane glycoproteins from the rough endoplasmic reticulum to the Golgi. A rate-limiting step in protein maturation and secretion. *J. Biol. Chem.* 263 2107–2110. 3276683

[B407] Lorek A. Takei Y. Cady E. B. Wyatt J. S. Penrice J. Edwards A. D. (1994). Delayed (“secondary”) cerebral energy failure after acute hypoxia-ischemia in the newborn piglet: continuous 48-hour studies by phosphorus magnetic resonance spectroscopy. *Pediatr. Res.* 36 699–706. 10.1203/00006450-199412000-00003 7898977

[B408] Loron G. Olivier P. See H. Le Sache N. Angulo L. Biran V. (2011). Ciprofloxacin prevents myelination delay in neonatal rats subjected to *E. coli* sepsis. *Ann. Neurol.* 69 341–351. 10.1002/ana.22190 21387379

[B409] Lubics A. Reglödi D. Tamás A. Kiss P. Szalai M. Szalontay L. (2005). Neurological reflexes and early motor behavior in rats subjected to neonatal hypoxic–ischemic injury. *Behav. Brain Res.* 157 157–165. 10.1016/j.bbr.2004.06.019 15617782

[B410] Ma J. Tong Y. Yu D. Mao M. (2012a). Tissue plasminogen activator-independent roles of neuroserpin in the central nervous system. *Neural Regen. Res.* 7 146–151. 10.3969/j.issn.1673-5374.2012.02.012 25767491 PMC4354132

[B411] Ma J. Yu D. Tong Y. Mao M. (2012b). Effect of neuroserpin in a neonatal hypoxic-ischemic injury model ex vivo. *Biol. Res.* 45 357–362. 10.4067/S0716-97602012000400005 23558991

[B412] Magnani D. Hasenpusch-Theil K. Theil T. (2013). Gli3 controls subplate formation and growth of cortical axons. *Cereb. Cortex* 23 2542–2551. 10.1093/cercor/bhs237 22903314

[B413] Malaeb S. N. Davis J. M. Pinz I. M. Newman J. L. Dammann O. Rios M. (2014). Effect of sustained postnatal systemic inflammation on hippocampal volume and function in mice. *Pediatr. Res.* 76 363–369. 10.1038/pr.2014.106 25003911 PMC4167932

[B414] Malik G. K. Trivedi R. Gupta R. K. Hasan K. M. Hasan M. Gupta A. (2006). Serial quantitative diffusion tensor MRI of the term neonates with hypoxic-ischemic encephalopathy (HIE). *Neuropediatrics* 37 337–343. 10.1055/s-2007-964869 17357035

[B415] Mallard C. Davidson J. O. Tan S. Green C. R. Bennet L. Robertson N. J. (2013). Astrocytes and microglia in acute cerebral injury underlying cerebral palsy associated with preterm birth. *Pediatr. Res.* 75 234–240. 10.1038/pr.2013.188 24336433 PMC11908707

[B416] Mallard C. Welin A. K. Peebles D. Hagberg H. Kjellmer I. (2003). White matter injury following systemic endotoxemia or asphyxia in the fetal sheep. *Neurochem. Res.* 28 215–223. 10.1023/A:1022368915400 12608695

[B417] Mallard E. C. Williams C. E. Gunn A. J. Gunning M. I. Gluckman P. D. (1993). Frequent episodes of brief ischemia sensitize the fetal sheep brain to neuronal loss and induce striatal injury. *Pediatr. Res.* 33 61–65. 10.1203/00006450-199301000-00013 8433863

[B418] Marini A. M. Jiang X. Wu X. Pan H. Guo Z. Mattson M. P. (2007). Preconditioning and neurotrophins: a model for brain adaptation to seizures, ischemia and other stressful stimuli. *Amino Acids* 32 299–304. 10.1007/s00726-006-0414-y 16998712

[B419] Marin-Padilla M. (1996). Developmental neuropathology and impact of perinatal brain damage. I: hemorrhagic lesions of neocortex. *J. Neuropathol. Exp. Neurol.* 55 758–773. 10.1097/00005072-199607000-00002 8965092

[B420] Marin-Padilla M. (1997). Developmental neuropathology and impact of perinatal brain damage. II: white matter lesions of the neocortex. *J. Neuropathol. Exp. Neurol.* 56 219–235. 10.1097/00005072-199703000-00001 9056536

[B421] Marin-Padilla M. (1999). Developmental neuropathology and impact of perinatal brain damage. III: gray matter lesions of the neocortex. *J. Neuropathol. Exp. Neurol.* 58 407–429. 10.1097/00005072-199905000-00001 10331430

[B422] Marques-Smith A. Lyngholm D. Kaufmann A. K. Stacey J. A. Hoerder-Suabedissen A. Becker E. B. (2016). A transient translaminar GABAergic interneuron circuit connects thalamocortical recipient layers in neonatal somatosensory cortex. *Neuron* 89 536–549. 10.1016/j.neuron.2016.01.015 26844833 PMC4742537

[B423] Marret S. Mukendi R. Gadisseux J. F. Gressens P. Evrard P. (1995). Effect of ibotenate on brain development: an excitotoxic mouse model of microgyria and posthypoxic-like lesions. *J. Neuropathol. Exp. Neurol.* 54 358–370. 10.1097/00005072-199505000-00009 7745435

[B424] Martin L. J. Brambrink A. Koehler R. C. Traystman R. J. (1997). Primary sensory and forebrain motor systems in the newborn brain are preferentially damaged by hypoxia-ischemia. *J. Compar. Neurol.* 377 262–285. 10.1002/(SICI)1096-9861(19970113)377:2<262::AID-CNE8>3.0.CO;2-1 8986885

[B425] Martin N. Bossenmeyer-Pourie C. Koziel V. Jazi R. Audonnet S. Vert P. (2012). Non-injurious neonatal hypoxia confers resistance to brain senescence in aged male rats. *PLoS ONE* 7:e48828 10.1371/journal.pone.0048828 PMC3500249 23173039

[B426] Martin R. Mozet C. Martin H. Welt K. Engel C. Fitzl G. (2011). The effect of Ginkgo biloba extract (EGb 761) on parameters of oxidative stress in different regions of aging rat brains after acute hypoxia. *Aging Clin. Exp. Res.* 23 255–263. 10.3275/7229 20802257

[B427] Martinez-Biarge M. Diez-Sebastian J. Kapellou O. Gindner D. Allsop J. M. Rutherford M. A. (2011). Predicting motor outcome and death in term hypoxic-ischemic encephalopathy. *Neurology* 76 2055–2061. 10.1212/WNL.0b013e31821f442d 21670434 PMC3111238

[B428] McDonald F. B. Dempsey E. M. O’Halloran K. D. (2016). Early life exposure to chronic intermittent hypoxia primes increased susceptibility to hypoxia-induced weakness in rat sternohyoid muscle during adulthood. *Front. Physiol.* 7:69 10.3389/fphys.2016.00069 PMC4777899 26973537

[B429] McDonald J. W. Behrens M. I. Chung C. Bhattacharyya T. Choi D. W. (1997). Susceptibility to apoptosis is enhanced in immature cortical neurons. *Brain Res.* 759 228–232. 10.1016/S0006-8993(97)00248-5 9221941

[B430] McDonald J. W. Johnston M. V. (1992). Neuroprotective synergism of 2-amino-3-phosphonoproprionate (D,L-AP3) and MK-801 against ibotenate induced brain injury. *Neurosci. Lett.* 145 213–216. 10.1016/0304-3940(92)90025-3 1361225

[B431] McDonald J. W. Roeser N. F. Silverstein F. S. Johnston M. V. (1989a). Quantitative assessment of neuroprotection against NMDA-induced brain injury. *Exp. Neurol.* 106 289–296. 2687017 10.1016/0014-4886(89)90162-3

[B432] McDonald J. W. Silverstein F. S. Cardona D. Hudson C. Chen R. Johnston M. V. (1990). Systemic administration of MK-801 protects against N-methyl-D-aspartate- and quisqualate-mediated neurotoxicity in perinatal rats. *Neuroscience* 36 589–599. 10.1016/0306-4522(90)90002-L 2234402

[B433] McDonald J. W. Silverstein F. S. Johnston M. V. (1988). Neurotoxicity of N-methyl-D-aspartate is markedly enhanced in developing rat central nervous system. *Brain Res.* 459 200–203. 10.1016/0006-8993(88)90306-X 3048538

[B434] McDonald J. W. Silverstein F. S. Johnston M. V. (1989b). Neuroprotective effects of MK-801, TCP, PCP and CPP against N-methyl-D-aspartate induced neurotoxicity in an in vivo perinatal rat model. *Brain Res.* 490 33–40. 2667694 10.1016/0006-8993(89)90427-7

[B435] McDonald J. W. Uckele J. Silverstein F. S. Johnston M. V. (1989c). HA-966 (1-hydroxy-3-aminopyrrolidone-2) selectively reduces N-methyl-D-aspartate (n.d.)-mediated brain damage. *Neurosci. Lett.* 104 167–170. 2682393 10.1016/0304-3940(89)90349-2

[B436] McKinstry R. C. Miller J. H. Snyder A. Z. Mathur A. Schefft G. L. Almli C. R. (2002). A prospective, longitudinal diffusion tensor imaging study of brain injury in newborns. *Neurology* 59 824–833. 10.1212/WNL.59.6.824 12297561

[B437] McLean C. Ferriero D. (2004). Mechanisms of hypoxic-ischemic injury in the term infant. *Semin. Perinatol.* 28 425–432. 10.1053/j.semperi.2004.10.005 15693399

[B438] McPherson R. J. Juul S. E. (2010). Erythropoietin for infants with hypoxic-ischemic encephalopathy. *Curr. Opin. Pediatr.* 22 139–145. 10.1097/MOP.0b013e328336eb57 20090525 PMC2879270

[B439] McQuillen P. S. Ferriero D. M. (2004). Selective vulnerability in the developing central nervous system. *Pediatr. Neurol.* 30 227–235. 10.1016/j.pediatrneurol.2003.10.001 15087099

[B440] McQuillen P. S. Ferriero D. M. (2005). Perinatal subplate neuron injury: implications for cortical development and plasticity. *Brain Pathol.* 15 250–260. 10.1111/j.1750-3639.2005.tb00528.x 16196392 PMC8096042

[B441] McQuillen P. S. Sheldon R. A. Shatz C. J. Ferriero D. M. (2003). Selective vulnerability of subplate neurons after early neonatal hypoxia-ischemia. *J. Neurosci.* 23 3308–3315. 12716938 10.1523/JNEUROSCI.23-08-03308.2003 PMC6742293

[B442] McRae A. Gilland E. Bona E. Hagberg H. (1995). Microglia activation after neonatal hypoxic-ischemia. *Dev. Brain Res.* 84 245–252. 10.1016/0165-3806(94)00177-2 7743644

[B443] Meldrum B. (1990). Protection against ischaemic neuronal damage by drugs acting on excitatory neurotransmission. *Cerebrovasc. Brain Metab. Rev.* 2 27–57. 2169834

[B444] Ment L. R. Bada H. S. Barnes P. Grant P. E. Hirtz D. Papile L. A. (2002). Practice parameter: neuroimaging of the neonate: report of the quality standards subcommittee of the american academy of neurology and the practice committee of the child neurology society. *Neurology* 58 1726–1738. 10.1212/WNL.58.12.1726 12084869

[B445] Ment L. R. Duncan C. C. Ehrenkranz R. A. (1984). Perinatal cerebral infarction. *Ann. Neurol.* 16 559–568. 10.1002/ana.410160506 6508239

[B446] Ment L. R. Stewart W. B. Ardito T. A. Madri J. A. (1991). Beagle pup germinal matrix maturation studies. *Stroke* 22 390–395. 10.1161/01.STR.22.3.390 2003309

[B447] Mercuri E. Ricci D. Cowan F. M. Lessing D. Frisone M. F. Haataja L. (2000). Head growth in infants with hypoxic-ischemic encephalopathy: correlation with neonatal magnetic resonance imaging. *Pediatrics* 106(2 Pt 1), 235–243. 10.1542/peds.106.2.235 10920145

[B448] Miguel P. M. Schuch C. P. Rojas J. J. Carletti J. V. Deckmann I. Martinato L. H. M. (2015). Neonatal hypoxia-ischemia induces attention-deficit hyperactivity disorder-like behavior in rats. *Behav. Neurosci.* 129 309–320. 10.1037/bne0000063 26030430

[B449] Mikati M. A. El Hokayem J. A. El Sabban M. E. (2007). Effects of a single dose of erythropoietin on subsequent seizure susceptibility in rats exposed to acute hypoxia at P10. *Epilepsia* 48 175–181. 10.1111/j.1528-1167.2006.00900.x 17241225

[B450] Mikati M. A. Zeinieh M. P. Kurdi R. M. Harb S. A. El Hokayem J. A. Daderian R. H. (2005). Long-term effects of acute and of chronic hypoxia on behavior and on hippocampal histology in the developing brain. *Dev. Brain Res.* 157 98–102. 10.1016/j.devbrainres.2005.03.007 15939090

[B451] Mikhailenko V. A. Butkevich I. P. Bagaeva T. R. Makukhina G. V. Otellin V. A. (2009). Short- and long-term influences of hypoxia during early postnatal period of development on behavioral and hormonal responses in rats. *Neurosci. Lett.* 464 214–217. 10.1016/j.neulet.2009.08.047 19703525

[B452] Miller S. L. Wallace E. M. Walker D. W. (2012). Antioxidant therapies: a potential role in perinatal medicine. *Neuroendocrinology* 96 13–23. 10.1159/000336378 22377769

[B453] Miller S. P. Ramaswamy V. Michelson D. Barkovich A. J. Holshouser B. Wycliffe N. (2005). Patterns of brain injury in term neonatal encephalopathy. *J. Pediatr.* 146 453–460. 10.1016/j.jpeds.2004.12.026 15812446

[B454] Miller S. P. Weiss J. Barnwell A. Ferriero D. M. Latal-Hajnal B. Ferrer-Rogers A. (2002). Seizure-associated brain injury in term newborns with perinatal asphyxia. *Neurology* 58 542–548. 10.1212/WNL.58.4.542 11865130

[B455] Miranda E. Lomas D. (2006). Neuroserpin: a serpin to think about. *Cell. Mol. Life Sci.* 63 709–722. 10.1007/s00018-005-5077-4 16465451 PMC11136441

[B456] Mishra O. P. Delivoria-Papadopoulos M. (1989). Lipid peroxidation in developing fetal guinea pig brain during normoxia and hypoxia. *Brain Res. Dev. Brain Res.* 45 129–135. 10.1016/0165-3806(89)90014-X 2917406

[B457] Mishra O. P. Delivoria-Papadopoulos M. (1999). Cellular mechanisms of hypoxic injury in the developing brain. *Brain Res. Bull.* 48 233–238. 10.1016/S0361-9230(98)00170-1 10229330

[B458] Mitha A. Foix-L’Helias L. Arnaud C. Marret S. Vieux R. Aujard Y. (2013). Neonatal infection and 5-year neurodevelopmental outcome of very preterm infants. *Pediatrics* 132 e372–80 10.1542/peds.2012-3979 23878051

[B459] Mohsenifar A. Lotfi A. S. Ranjbar B. Allameh A. Zaker F. Hasani L. (2007). A study of the oxidation-induced conformational and functional changes in neuroserpin. *Iran. Biomed. J.* 11 41–46. 18051703

[B460] Molnár Z. Adams R. Blakemore C. (1998). Mechanisms underlying the early establishment of thalamocortical connections in the rat. *J. Neurosci.* 18 5723–5745. 9671663 10.1523/JNEUROSCI.18-15-05723.1998 PMC6793053

[B461] Moorcraft J. Bolas N. M. Ives N. K. Ouwerkerk R. Smyth J. Rajagopalan B. (1991). Global and depth resolved phosphorus magnetic resonance spectroscopy to predict outcome after birth asphyxia. *Arch. Dis. Child* 66 1119–1123. 10.1136/adc.66.10_Spec_No.1119 1750759 PMC1590291

[B462] Mortola J. P. (1999). How newborn mammals cope with hypoxia. *Respir. Physiol.* 116 95–103. 10.1016/S0034-5687(99)00038-9 10487295

[B463] Mortola J. P. Naso L. (1998). Thermogenesis in newborn rats after prenatal or postnatal hypoxia. *J. Appl. Physiol.* 85 84–90. 9655759 10.1152/jappl.1998.85.1.84

[B464] Munuswamy-Ramanujam G. Dai E. Liu L. Shnabel M. Sun Y. M. Bartee M. (2010). Neuroserpin, a thrombolytic serine protease inhibitor (serpin), blocks transplant vasculopathy with associated modification of T-helper cell subsets. *Thromb. Haemost.* 103 545–555. 10.1160/TH09-07-0441 20135065

[B465] Muramatsu K. Fukuda A. Togari H. Wada Y. Nishino H. (1997). Vulnerability to cerebral hypoxic-ischemic insult in neonatal but not in adult rats is in parallel with disruption of the blood-brain barrier. *Stroke* 28 2281–2288; discussion 2288–2289. 10.1161/01.str.28.11.2281 9368577

[B466] Murray D. M. Bala P. O’Connor C. M. Ryan C. A. Connolly S. Boylan G. B. (2010). The predictive value of early neurological examination in neonatal hypoxic-ischaemic encephalopathy and neurodevelopmental outcome at 24 months. *Dev. Med. Child Neurol.* 52 e55–e59. 10.1111/j.1469-8749.2009.03550.x 20041933

[B467] Murray D. M. Boylan G. B. Ryan C. A. Connolly S. (2009). Early EEG findings in hypoxic-ischemic encephalopathy predict outcomes at 2 years. *Pediatrics* 124 e459–e467. 10.1542/peds.2008-2190 19706569

[B468] Murray D. M. O’Connor C. M. Ryan C. A. Korotchikova I. Boylan G. B. (2016). Early EEG grade and outcome at 5 years after mild neonatal hypoxic ischemic encephalopathy. *Pediatrics* 138 e20160659 10.1542/peds.2016-0659 27650049

[B469] Myers M. (1975). “Investigation of skin flap necrosis,” in *Skin Flaps*, eds Grabb W. C. Myers M. B. (Boston, MA: Little, Brown and Company), 3.

[B470] Nagel S. Papadakis M. Pfleger K. Grond-Ginsbach C. Buchan A. Wagner S. (2012). Microarray analysis of the global gene expression profile following hypothermia and transient focal cerebral ischemia. *Neuroscience* 208 109–122. 10.1016/j.neuroscience.2012.01.048 22366221

[B471] Nakajima W. Ishida A. Lange M. S. Gabrielson K. L. Wilson M. A. Martin L. J. (2000). Apoptosis has a prolonged role in the neurodegeneration after hypoxic ischemia in the newborn rat. *J. Neurosci.* 20 7994–8004. 11050120 10.1523/JNEUROSCI.20-21-07994.2000 PMC6772742

[B472] Nakajima W. Ishida A. Takada G. (1997). Magnesium attenuates a striatal dopamine increase induced by anoxia in the neonatal rat brain: an in vivo microdialysis study. *Pediatr. Res.* 41 809–814. 10.1203/00006450-199706000-00003 9167193

[B473] Nalivaeva N. N. Fisk L. Aviles R. M. C. Plesneva S. A. Zhuravin I. A. Turner A. J. (2003). Effects of prenatal hypoxia on expression of amyloid precursor protein and metallopeptidases in the rat brain. *Lett. Peptide Sci.* 10 455–462. 10.1007/BF02442577

[B474] Nash K. B. Bonifacio S. L. Glass H. C. Sullivan J. E. Barkovich A. J. Ferriero D. M. (2011). Video-EEG monitoring in newborns with hypoxic-ischemic encephalopathy treated with hypothermia. *Neurology* 76 556–562. 10.1212/WNL.0b013e31820af91a 21300971 PMC3053178

[B475] Navarova J. Schmidtova M. Ujhazy E. Dubovicky M. Mach M. (2006). Selected biochemical variables in a model of neonatal anoxia in rats. *Neuro Endocrinol. Lett.* 27(Suppl. 2), 78–81. 17159785

[B476] Neil J. Miller J. Mukherjee P. Huppi P. S. (2002). Diffusion tensor imaging of normal and injured developing human brain - a technical review. *NMR Biomed.* 15 543–552. 10.1002/nbm.784 12489100

[B477] Ness J. K. Romanko M. J. Rothstein R. P. Wood T. L. Levison S. W. (2001). Perinatal hypoxia-ischemia induces apoptotic and excitotoxic death of periventricular white matter oligodendrocyte progenitors. *Dev. Neurosci.* 23 203–208. 10.1159/000046144 11598321

[B478] Neufeld M. D. Frigon C. Graham A. S. Mueller B. A. (2005). Maternal infection and risk of cerebral palsy in term and preterm infants. *J. Perinatol.* 25 108–113. 10.1038/sj.jp.7211219 15538398

[B479] Nguyen V. McQuillen P. S. (2010). AMPA and metabotropic excitoxicity explain subplate neuron vulnerability. *Neurobiol. Dis.* 37 195–207. 10.1016/j.nbd.2009.10.002 19822212 PMC2789448

[B480] Nicole O. Docagne F. Ali C. Margaill I. Carmeliet P. MacKenzie E. T. (2001). The proteolytic activity of tissue-plasminogen activator enhances NMDA receptor-mediated signaling. *Nat. Med.* 7 59–64. 10.1038/83358 11135617

[B481] Nijboer C. H. Groenendaal F. Kavelaars A. Hagberg H. H. van Bel F. Heijnen C. J. (2007). Gender-specific neuroprotection by 2-iminobiotin after hypoxia-ischemia in the neonatal rat via a nitric oxide independent pathway. *J. Cereb. Blood Flow Metab.* 27 282–292. 10.1038/sj.jcbfm.9600342 16736041

[B482] Nijboer C. H. van der Kooij M. A. van Bel F. Ohl F. Heijnen C. J. Kavelaars A. (2010). Inhibition of the JNK/AP-1 pathway reduces neuronal death and improves behavioral outcome after neonatal hypoxic–ischemic brain injury. *Brain Behav. Immun.* 24 812–821. 10.1016/j.bbi.2009.09.008 19766183

[B483] Nikolov N. M. Cunningham A. J. (2003). Mild therapeutic hypothermia to improve the neurologic outcome after cardiac arrest. *Surv. Anesthesiol.* 47 219–220. 10.1097/01.sa.0000087691.31092.12

[B484] Nobuta H. Ghiani C. A. Paez P. M. Spreuer V. Dong H. Korsak R. A. (2012). STAT3-mediated astrogliosis protects myelin development in neonatal brain injury. *Ann. Neurol.* 72 750–765. 10.1002/ana.23670 22941903 PMC3514566

[B485] Noh M. R. Kim S. K. Sun W. Park S. K. Choi H. C. Lim J. H. (2006). Neuroprotective effect of topiramate on hypoxic ischemic brain injury in neonatal rats. *Exp. Neurol.* 201 470–478. 10.1016/j.expneurol.2006.04.038 16884714

[B486] Northington F. J. Chavez-Valdez R. Martin L. J. (2011). Neuronal cell death in neonatal hypoxia-ischemia. *Ann. Neurol.* 69 743–758. 10.1002/ana.22419 21520238 PMC4000313

[B487] Northington F. J. Ferriero D. M. Graham E. M. Traystman R. J. Martin L. J. (2001). Early neurodegeneration after hypoxia-ischemia in neonatal rat is necrosis while delayed neuronal death is apoptosis. *Neurobiol. Dis.* 8 207–219. 10.1006/nbdi.2000.0371 11300718

[B488] Northington F. J. Graham E. M. Martin L. J. (2005). Apoptosis in perinatal hypoxic–ischemic brain injury: how important is it and should it be inhibited? *Brain Res. Rev.* 50 244–257. 10.1016/j.brainresrev.2005.07.003 16216332

[B489] Northington F. J. Zelaya M. E. O’Riordan D. P. Blomgren K. Flock D. L. Hagberg H. (2007). Failure to complete apoptosis following neonatal hypoxia-ischemia manifests as “continuum” phenotype of cell death and occurs with multiple manifestations of mitochondrial dysfunction in rodent forebrain. *Neuroscience* 149 822–833. 10.1016/j.neuroscience.2007.06.060 17961929 PMC3947608

[B490] Novikoff A. B. (1976). The endoplasmic reticulum: a cytochemist’s view (a review). *Proc. Natl. Acad. Sci. U.S.A.* 73 2781–2787. 10.1073/pnas.73.8.2781 183210 PMC430742

[B491] O’Brien J. S. Sampson E. L. (1965). Myelin Membrane: a molecular abnormality. *Science* 150 1613–1614. 10.1126/science.150.3703.1613 5866661

[B492] O’Connell B. Moritz K. Walker D. Dickinson H. (2013). Treatment of pregnant spiny mice at mid gestation with a synthetic glucocorticoid has sex-dependent effects on placental glycogen stores. *Placenta* 34 932–940. 10.1016/j.placenta.2013.06.310 23896029

[B493] Oeschger F. M. Wang W. Z. Lee S. Garcia-Moreno F. Goffinet A. M. Arbones M. L. (2012). Gene expression analysis of the embryonic subplate. *Cereb. Cortex* 22 1343–1359. 10.1093/cercor/bhr197 21862448 PMC4972418

[B494] Oguni H. Sugama M. Osawa M. (2008). Symptomatic parieto-occipital epilepsy as sequela of perinatal asphyxia. *Pediatr. Neurol.* 38 345–352. 10.1016/j.pediatrneurol.2007.10.016 18410851

[B495] Okiyoneda T. Harada K. Takeya M. Yamahira K. Wada I. Shuto T. (2004). Delta F508 CFTR pool in the endoplasmic reticulum is increased by calnexin overexpression. *Mol. Biol. Cell* 15 563–574. 10.1091/mbc.E03-06-0379 14595111 PMC329241

[B496] Okusa C. Oeschger F. Ginet V. Wang W. Z. Hoerder-Suabedissen A. Matsuyama T. (2014). Subplate in a rat model of preterm hypoxia-ischemia. *Ann. Clin. Transl. Neurol.* 1 679–691. 10.1002/acn3.97 25493282 PMC4241795

[B497] Omouendze P. L. Henry V. J. Porte B. Dupré N. Carmeliet P. Gonzalez B. J. (2013). Hypoxia-ischemia or excitotoxin-induced tissue plasminogen activator-dependent gelatinase activation in mice neonate brain microvessels. *PLoS ONE* 8:e71263 10.1371/journal.pone.0071263 PMC3735506 23940734

[B498] Osterwalder T. Cinelli P. Baici A. Pennella A. Krueger S. R. Schrimpf S. P. (1998). The axonally secreted serine proteinase inhibitor, neuroserpin, inhibits plasminogen activators and plasmin but not thrombin. *J. Biol. Chem.* 273 2312–2321. 10.1074/jbc.273.4.2312 9442076

[B499] Osterwalder T. Contartese J. Stoeckli E. T. Kuhn T. B. Sonderegger P. (1996). Neuroserpin, an axonally secreted serine protease inhibitor. *EMBO J.* 15 2944–2953. 8670795 PMC450235

[B500] Otoya R. E. Seltzer A. M. Donoso A. O. (1997). Acute and long-lasting effects of neonatal hypoxia on ( )-3-[125 I] MK-801 binding to NMDA brain receptors. *Exp. Neurol.* 148 92–99. 10.1006/exnr.1997.6612 9400422

[B501] Ozer E. A. Yilmaz O. Akhisaroglu M. Tuna B. Bakiler A. R. Ozer E. (2002). Heat shock protein 70 expression in neonatal rats after hypoxic stress. *J. Matern Fetal Neonatal Med.* 12 112–117. 10.1080/jmf.12.2.112.117 12420841

[B502] Palmer C. Vannucci R. C. Towfighi J. (1990). Reduction of perinatal hypoxic-ischemic brain damage with allopurinol. *Pediatr. Res.* 27(4 Pt 1), 332–336. 10.1203/00006450-199004000-00003 2342827

[B503] Pang Y. Rodts-Palenik S. Cai Z. Bennett W. A. Rhodes P. G. (2005). Suppression of glial activation is involved in the protection of IL-10 on maternal *E. coli* induced neonatal white matter injury. *Brain Res. Dev. Brain Res.* 157 141–149. 10.1016/j.devbrainres.2005.03.015 15878785

[B504] Park D. Shin K. Choi E. Choi Y. Jang J. Kim J. (2015). Protective effects of N-acetyl-l-cysteine in human oligodendrocyte progenitor cells and restoration of motor function in neonatal rats with hypoxic-ischemic encephalopathy. *Evid. Based Complement. Alternat. Med.* 2015:764251 10.1155/2015/764251 PMC4396975 25918547

[B505] Park W. S. Sung S. I. Ahn S. Y. Yoo H. S. Sung D. K. Im G. H. (2015). Hypothermia augments neuroprotective activity of mesenchymal stem cells for neonatal hypoxic-ischemic encephalopathy. *PLoS ONE* 10:e0120893 10.1371/journal.pone.0120893 PMC4376738 25816095

[B506] Parolini O. Alviano F. Bergwerf I. Boraschi D. De Bari C. De Waele P. (2010). Toward cell therapy using placenta-derived cells: disease mechanisms, cell biology, preclinical studies, and regulatory aspects at the round table. *Stem Cells Dev.* 19 143–154. 10.1089/scd.2009.0404 19947828

[B507] Partanen E. Kujala T. Naatanen R. Liitola A. Sambeth A. Huotilainen M. (2013). Learning-induced neural plasticity of speech processing before birth. *Proc. Natl. Acad. Sci. U.S.A.* 110 15145–15150. 10.1073/pnas.1302159110 23980148 PMC3773755

[B508] Pauliah S. S. Shankaran S. Wade A. Cady E. B. Thayyil S. (2013). Therapeutic hypothermia for neonatal encephalopathy in low- and middle-income countries: a systematic review and meta-analysis. *PLoS ONE* 8:e58834 10.1371/journal.pone.0058834 PMC3602578 23527034

[B509] Paulose C. S. Chathu F. Khan S. R. Krishnakumar A. (2008). Neuroprotective role of *Bacopa monnieri* extract in epilepsy and effect of glucose supplementation during hypoxia: glutamate receptor gene expression. *Neurochem. Res.* 33 1663–1671. 10.1007/s11064-007-9513-8 17940877

[B510] Pazaiti A. Soubasi V. Spandou E. Karkavelas G. Georgiou T. Karalis P. (2009). Evaluation of long-lasting sensorimotor consequences following neonatal hypoxic-ischemic brain injury in rats: the neuroprotective role of MgSO4. *Neonatology* 95 33–40. 10.1159/000151753 18787335

[B511] Pazos M. Cinquina V. Gomez A. Layunta R. Santos M. Fernández-Ruiz J. (2012). Cannabidiol administration after hypoxia–ischemia to newborn rats reduces long-term brain injury and restores neurobehavioral function. *Neuropharmacology* 63 776–783. 10.1016/j.neuropharm.2012.05.034 22659086

[B512] Pedraza M. Hoerder-Suabedissen A. Albert-Maestro M. A. Molnár Z. De Carlos J. A. (2014). Extracortical origin of some murine subplate cell populations. *Proc. Natl. Acad. Sci. U.S.A.* 111 8613–8618. 10.1073/pnas.1323816111 24778253 PMC4060693

[B513] Peebles D. M. Miller S. Newman J. P. Scott R. Hanson M. A. (2003). The effect of systemic administration of lipopolysaccharide on cerebral haemodynamics and oxygenation in the 0.*65* gestation ovine fetus in utero. *BJOG* 110 735–743. 10.1111/j.1471-0528.2003.02152.x 12892685

[B514] Pelham H. R. (1989). Heat shock and the sorting of luminal ER proteins. *EMBO J.* 8 3171–3176. 2684638 10.1002/j.1460-2075.1989.tb08475.x PMC401431

[B515] Pellitteri R. Catania M. V. Bonaccorso C. M. Ranno E. Dell’Albani P. Zaccheo D. (2014). Viability of olfactory ensheathing cells after hypoxia and serum deprivation: Implication for therapeutic transplantation. *J. Neurosci. Res.* 92 1757–1766. 10.1002/jnr.23442 24975631

[B516] Peng Z. Li J. Li Y. Yang X. Feng S. Han S. (2013). Downregulation of miR-181b in mouse brain following ischemic stroke induces neuroprotection against ischemic injury through targeting heat shock protein A5 and ubiquitin carboxyl-terminal hydrolase isozyme L1. *J. Neurosci. Res.* 91 1349–1362. 10.1002/jnr.23255 23900885

[B517] Penrice J. Lorek A. Cady E. B. Amess P. N. Wylezinska M. Cooper C. E. (1997). Proton magnetic resonance spectroscopy of the brain during acute hypoxia-ischemia and delayed cerebral energy failure in the newborn piglet. *Pediatr. Res.* 41 795–802. 10.1203/00006450-199706000-00001 9167191

[B518] Pereira L. O. Arteni N. S. Petersen R. C. da Rocha A. P. Achaval M. Netto C. A. (2007). Effects of daily environmental enrichment on memory deficits and brain injury following neonatal hypoxia-ischemia in the rat. *Neurobiol. Learn. Mem.* 87 101–108. 10.1016/j.nlm.2006.07.003 16931063

[B519] Perera P. N. D. Hu Q. Tang J. Li L. Barnhart M. Doycheva D. M. (2014). Delayed remote ischemic postconditioning improves long term sensory motor deficits in a neonatal hypoxic ischemic rat model. *PLoS ONE* 9:e90258 10.1371/journal.pone.0090258 PMC3938659 24587303

[B520] Perlman J. M. (1997). Intrapartum hypoxic-ischemic cerebral injury and subsequent cerebral palsy: medicolegal issues. *Pediatrics* 99 851–859. 10.1542/peds.99.6.851 9164779

[B521] Perlman J. M. (2006). Summary proceedings from the neurology group on hypoxic-ischemic encephalopathy. *Pediatrics* 117(Suppl. 1), S28–S33. 10.1542/peds.2005-0620E 16777819

[B522] Perlman J. M. Risser R. (1993). Severe fetal acidemia: neonatal neurologic features and short-term outcome. *Pediatr. Neurol.* 9 277–282. 10.1016/0887-8994(93)90063-I 8216539

[B523] Perrin R. G. Rutka J. T. Drake J. M. Meltzer H. Hellman J. Jay V. (1997). Management and outcomes of posterior fossa subdural hematomas in neonates. *Neurosurgery* 40 1190–1199; discussion 1199–1200. 10.1097/00006123-199706000-00016 9179892

[B524] Petersson K. H. Pinar H. Stopa E. G. Faris R. A. Sadowska G. B. Hanumara R. C. (2002). White matter injury after cerebral ischemia in ovine fetuses. *Pediatr. Res.* 51 768–776. 10.1203/00006450-200206000-00019 12032276

[B525] Pfeffer S. R. Rothman J. E. (1987). Biosynthetic protein transport and sorting by the endoplasmic reticulum and Golgi. *Annu. Rev. Biochem.* 56 829–852. 10.1146/annurev.bi.56.070187.004145 3304148

[B526] Placencia F. X. Kong L. Weisman L. E. (2009). Treatment of methicillin-resistant *Staphylococcus aureus* in neonatal mice: lysostaphin versus vancomycin. *Pediatr. Res.* 65 420–424. 10.1203/PDR.0b013e3181994a53 19127212

[B527] Poggi S. H. Park J. Toso L. Abebe D. Roberson R. Woodard J. E. (2005). No phenotype associated with established lipopolysaccharide model for cerebral palsy. *Am. J. Obstet. Gynecol.* 192 727–733. 10.1016/j.ajog.2004.12.053 15746664

[B528] Pogledic I. Kostovic I. Fallet-Bianco C. Adle-Biassette H. Gressens P. Verney C. (2014). Involvement of the subplate zone in preterm infants with periventricular white matter injury. *Brain Pathol.* 24 128–141. 10.1111/bpa.12096 25003178 PMC8029368

[B529] Portera-Cailliau C. Price D. L. Martin L. J. (1997a). Excitotoxic neuronal death in the immature brain is an apoptosis-necrosis morphological continuum. *J. Comp. Neurol.* 378 70–87. 10.1002/(sici)1096-9861(19970203)378:1<10::aid-cne4>3.0.co;2-n 9120055

[B530] Portera-Cailliau C. Price D. L. Martin L. J. (1997b). Non-NMDA and NMDA receptor-mediated excitotoxic neuronal deaths in adult brain are morphologically distinct: further evidence for an apoptosis-necrosis continuum. *J. Comp. Neurol.* 378 88–104. 9120056

[B531] Pourie G. Blaise S. Trabalon M. Nedelec E. Gueant J. L. Daval J. L. (2006). Mild, non-lesioning transient hypoxia in the newborn rat induces delayed brain neurogenesis associated with improved memory scores. *Neuroscience* 140 1369–1379. 10.1016/j.neuroscience.2006.02.083 16650606

[B532] Price D. J. Aslam S. Tasker L. Gillies K. (1997). Fates of the earliest generated cells in the developing murine neocortex. *J. Comp. Neurol.* 377 414–422. 10.1002/(SICI)1096-9861(19970120)377:3<414::AID-CNE8>3.0.CO;2-5 8989655

[B533] Pryds O. Greisen G. Lou H. Friis-Hansen B. (1990). Vasoparalysis associated with brain damage in asphyxiated term infants. *J. Pediatr.* 117(1 Pt 1), 119–125. 10.1016/s0022-3476(05)72459-8 2115079

[B534] Pu Y. Garg A. Corby R. Gao J. H. Zeng C. M. Pu Y. (2008). A positive correlation between alpha-glutamate and glutamine on brain 1H-MR spectroscopy and neonatal seizures in moderate and severe hypoxic-ischemic encephalopathy. *AJNR Am. J. Neuroradiol.* 29 216 10.3174/ajnr.A0798 PMC8119007 17974609

[B535] Pulera M. R. Adams L. M. Liu H. Santos D. G. Nishimura R. N. Yang F. (1998). Apoptosis in a neonatal rat model of cerebral hypoxia-ischemia. *Stroke* 29 2622–2630. 10.1161/01.STR.29.12.2622 9836776

[B536] Puyal J. Vaslin A. Mottier V. Clarke P. G. (2009). Postischemic treatment of neonatal cerebral ischemia should target autophagy. *Ann. Neurol.* 66 378–389. 10.1002/ana.21714 19551849

[B537] Qian Z. Gilbert M. E. Colicos M. A. Kandel E. R. Kuhl D. (1993). Tissue-plasminogen activator is induced as an immediate–early gene during seizure, kindling and long-term potentiation. *Nature* 361 453–457. 10.1038/361453a0 8429885

[B538] Rabi Y. Rabi D. Yee W. (2007). Room air resuscitation of the depressed newborn: a systematic review and meta-analysis. *Resuscitation* 72 353–363. 10.1016/j.resuscitation.2006.06.134 17240032

[B539] Rakhade S. N. Jensen F. E. (2009). Epileptogenesis in the immature brain: emerging mechanisms. *Nat. Rev. Neurol.* 5 380–391. 10.1038/nrneurol.2009.80 19578345 PMC2822660

[B540] Rakhade S. N. Klein P. M. Huynh T. Hilario-Gomez C. Kosaras B. Rotenberg A. (2011). Development of later life spontaneous seizures in a rodent model of hypoxia-induced neonatal seizures. *Epilepsia* 52 753–765. 10.1111/j.1528-1167.2011.02992.x 21366558 PMC3071424

[B541] Rastogi R. N. Prichard J. S. Lowden J. A. (1968). Elevation of phosphorus levels in serum and decreased rain content of gangliosides in rats following neonatal asphyxia. *Pediatr. Res.* 2 125–130. 10.1203/00006450-196803000-00008 5694911

[B542] Raveendran A. T. Skaria P. C. (2013). Learning and cognitive deficits in hypoxic neonatal rats intensified by BAX mediated apoptosis: protective role of glucose, oxygen, and epinephrine. *Int. J. Neurosci.* 123 80–88. 10.3109/00207454.2012.731457 22998365

[B543] Reddy K. Mallard C. Guan J. Marks K. Bennet L. Gunning M. (1998). Maturational change in the cortical response to hypoperfusion injury in the fetal sheep. *Pediatr. Res.* 43 674–682. 10.1203/00006450-199805000-00017 9585015

[B544] Redline R. W. O’Riordan M. A. (2000). Placental lesions associated with cerebral palsy and neurologic impairment following term birth. *Arch. Pathol. Lab. Med.* 124 1785–1791. 11100058 10.5858/2000-124-1785-PLAWCP

[B545] Rennie J. M. Chorley G. Boylan G. B. Pressler R. Nguyen Y. Hooper R. (2004). Non-expert use of the cerebral function monitor for neonatal seizure detection. *Arch. Dis. Child. Fetal Neonatal Ed.* 89 F37–F40. 10.1136/fn.89.1.f37 14711852 PMC1721641

[B546] Represa A. Tremblay E. Ben-Ari Y. (1989). Transient increase of NMDA-binding sites in human hippocampus during development. *Neurosci. Lett.* 99 61–66. 10.1016/0304-3940(89)90265-6 2568608

[B547] Ricagno S. Caccia S. Sorrentino G. Antonini G. Bolognesi M. (2009). Human neuroserpin: structure and time-dependent inhibition. *J. Mol. Biol.* 388 109–121. 10.1016/j.jmb.2009.02.056 19265707

[B548] Ricagno S. Pezzullo M. Barbiroli A. Manno M. Levantino M. Santangelo M. G. (2010). Two latent and two hyperstable polymeric forms of human neuroserpin. *Biophys. J.* 99 3402–3411. 10.1016/j.bpj.2010.09.021 21081089 PMC2980742

[B549] Rice J. E. Vannucci R. C. Brierley J. B. (1981). The influence of immaturity on hypoxic-ischemic brain damage in the rat. *Ann. Neurol.* 9 131–141. 10.1002/ana.410090206 7235629

[B550] Richardson B. Korkola S. Asano H. Challis J. Polk D. Fraser M. (1996). Regional blood flow and the endocrine response to sustained hypoxemia in the preterm ovine fetus. *Pediatr. Res.* 40 337–343. 10.1203/00006450-199608000-00024 8827787

[B551] Richardson B. S. Bocking A. D. (1998). Metabolic and circulatory adaptations to chronic hypoxia in the fetus. *Comp. Biochem. Physiol. A Mol. Integr. Physiol.* 119 717–723. 10.1016/S1095-6433(98)01010-1 9683411

[B552] Richardson B. S. Carmichael L. Homan J. Johnston L. Gagnon R. (1996). Fetal cerebral, circulatory, and metabolic responses during heart rate decelerations with umbilical cord compression. *Am. J. Obstet. Gynecol.* 175(4 Pt 1), 929–936. 10.1016/S0002-9378(96)80027-5 8885750

[B553] Richer L. P. Shevell M. I. Miller S. P. (2001). Diagnostic profile of neonatal hypotonia: an 11-year study. *Pediatr. Neurol.* 25 32–37. 10.1016/S0887-8994(01)00277-6 11483393

[B554] Richetto J. Calabrese F. Meyer U. Riva M. A. (2013). Prenatal versus postnatal maternal factors in the development of infection-induced working memory impairments in mice. *Brain Behav. Immun.* 33 190–200. 10.1016/j.bbi.2013.07.006 23876745

[B555] Riikonen R. S. Kero P. O. Simell O. G. (1992). Excitatory amino acids in cerebrospinal fluid in neonatal asphysia. *Pediatr. Neurol.* 8 37–40. 10.1016/0887-8994(92)90050-9 1348414

[B556] Rijken D. Wijngaards G. Welbergen J. (1980). Relationship between tissue plasminogen activator and the activators in blood and vascular wall. *Thromb. Res.* 18 815–830. 10.1016/0049-3848(80)90204-2 7191151

[B557] Rijken D. Wijngaards G. Zaal-de Jong M. Welbergen J. (1979). Purification and partial characterization of plasminogen activator from human uterine tissue. *Biochim. Biophys. Acta* 580 140–153. 10.1016/0005-2795(79)90205-8 121055

[B558] Robertson C. Finer N. (1985). Term infants with hypoxic-ischemic encephalopathy: outcome at 3.5 years. *Dev. Med. Child Neurol.* 27 473–484. 10.1111/j.1469-8749.1985.tb04571.x 4029517

[B559] Robertson C. Finer N. Grace M. (1989). School performance of survivors of neonatal encephalopathy associated with birth asphyxia at term. *J. Pediatr.* 114 753–760. 10.1016/S0022-3476(89)80132-5 2469789

[B560] Robertson J. D. Enoksson M. Suomela M. Zhivotovsky B. Orrenius S. (2002). Caspase-2 acts upstream of mitochondria to promote cytochrome c release during etoposide-induced apoptosis. *J. Biol. Chem.* 277 29803–29809. 10.1074/jbc.M204185200 12065594

[B561] Robertson N. J. Cowan F. M. Cox I. J. Edwards A. D. (2002). Brain alkaline intracellular pH after neonatal encephalopathy. *Ann. Neurol.* 52 732–742. 10.1002/ana.10365 12447926

[B562] Robertson N. J. Cox I. J. Cowan F. M. Counsell S. J. Azzopardi D. Edwards A. D. (1999). Cerebral intracellular lactic alkalosis persisting months after neonatal encephalopathy measured by magnetic resonance spectroscopy. *Pediatr. Res.* 46 287–296. 10.1203/00006450-199909000-00007 10473043

[B563] Robertson N. J. Iwata O. (2007). Bench to bedside strategies for optimizing neuroprotection following perinatal hypoxia–ischaemia in high and low resource settings. *Early Hum. Dev.* 83 801–811. 10.1016/j.earlhumdev.2007.09.015 17964091

[B564] Robinson S. Li Q. Dechant A. Cohen M. L. (2006). Neonatal loss of gamma-aminobutyric acid pathway expression after human perinatal brain injury. *J. Neurosurg.* 104 6(Suppl.), 396–408. 16776375 10.3171/ped.2006.104.6.396 PMC1762128

[B565] Rocha-Ferreira E. Hristova M. (2015). Antimicrobial peptides and complement in neonatal hypoxia-ischemia induced brain damage. *Front. Immunol.* 6:56 10.3389/fimmu.2015.00056 PMC4325932 25729383

[B566] Rodewald A. K. Onderdonk A. B. Warren H. B. Kasper D. L. (1992). Neonatal mouse model of group B streptococcal infection. *J. Infect. Dis.* 166 635–639. 10.1093/infdis/166.3.635 1500748

[B567] Rodriguez-Alvarez N. Jimenez-Mateos E. M. Dunleavy M. Waddington J. L. Boylan G. B. Henshall D. C. (2015). Effects of hypoxia-induced neonatal seizures on acute hippocampal injury and later-life seizure susceptibility and anxiety-related behavior in mice. *Neurobiol. Dis.* 83 100–114. 10.1016/j.nbd.2015.08.023 26341542

[B568] Rodríguez-González R. Agulla J. Pérez-Mato M. Sobrino T. Castillo J. (2011a). Neuroprotective effect of neuroserpin in rat primary cortical cultures after oxygen and glucose deprivation and tPA. *Neurochem. Int.* 58 337–343. 10.1016/j.neuint.2010.12.006 21163314

[B569] Rodríguez-González R. Millán M. Sobrino T. Miranda E. Brea D. de la Ossa N. P. (2011b). The natural tissue plasminogen activator inhibitor neuroserpin and acute ischaemic stroke outcome. *Thromb. Haemost.* 105 421–429. 10.1160/TH10-09-0621 21174006

[B570] Rodríguez-González R. Sobrino T. Rodríguez-Yáñez M. Millán M. Brea D. Miranda E. (2011c). Association between neuroserpin and molecular markers of brain damage in patients with acute ischemic stroke. *J. Transl. Med.* 9:58 10.1186/1479-5876-9-58 PMC3113955 21569344

[B571] Rodts-Palenik S. Wyatt-Ashmead J. Pang Y. Thigpen B. Cai Z. Rhodes P. (2004). Maternal infection-induced white matter injury is reduced by treatment with interleukin-10. *Am. J. Obstet. Gynecol.* 191 1387–1392. 10.1016/j.ajog.2004.06.093 15507970

[B572] Rogalska J. Caputa M. (2010). Neonatal asphyxia under hyperthermic conditions alters HPA axis function in juvenile rats. *Neurosci. Lett.* 472 68–72. 10.1016/j.neulet.2010.01.060 20122989

[B573] Rogalska J. Caputa M. Wentowska K. Nowakowska A. (2004). Stress-induced behaviour in juvenile rats: effects of neonatal asphyxia, body temperature and chelation of iron. *Behav. Brain Res.* 154 321–329. 10.1016/j.bbr.2004.02.020 15313019

[B574] Rogalska J. Danielisova V. Caputa M. (2006). Effect of neonatal body temperature on postanoxic, potentially neurotoxic iron accumulation in the rat brain. *Neurosci. Lett.* 393 249–254. 10.1016/j.neulet.2005.09.085 16289321

[B575] Rogalska J. Kang P. Wotherspoon W. Macleod M. R. Lai M. (2009). Effect of hyperthermia and anoxia on glucocorticoid and mineralocorticoid receptor expression in neonatal rat hippocampus. *Neurosci. Lett.* 450 196–200. 10.1016/j.neulet.2008.11.033 19028552

[B576] Rognlien A. G. W. Wollen E. J. Atneosen-Åsegg M. Saugstad O. D. (2014). Increased expression of inflammatory genes in the neonatal mouse brain after hyperoxic reoxygenation. *Pediatr. Res.* 77 326–333. 10.1038/pr.2014.193 25423075

[B577] Roohey T. Raju T. N. Moustogiannis A. N. (1997). Animal models for the study of perinatal hypoxic-ischemic encephalopathy: a critical analysis. *Early Hum. Dev.* 47 115–146. 10.1016/S0378-3782(96)01773-2 9039963

[B578] Rorke L. B. (1992). Anatomical features of the developing brain implicated in pathogenesis of hypoxic-ischemic injury. *Brain Pathol.* 2 211–221. 10.1111/j.1750-3639.1992.tb00694.x 1343836

[B579] Roth S. C. Edwards A. D. Cady E. B. Delpy D. T. Wyatt J. S. Azzopardi D. (1992). Relation between cerebral oxidative metabolism following birth asphyxia, and neurodevelopmental outcome and brain growth at one year. *Dev. Med. Child Neurol.* 34 285–295. 10.1111/j.1469-8749.1992.tb11432.x 1572514

[B580] Rotstein M. Bassan H. Kariv N. Speiser Z. Harel S. Gozes I. (2006). NAP enhances neurodevelopment of newborn apolipoprotein E-deficient mice subjected to hypoxia. *J. Pharmacol. Exp. Ther.* 319 332–339. 10.1124/jpet.106.106898 16822898

[B581] Rousset C. I. Chalon S. Cantagrel S. Bodard S. Andres C. Gressens P. (2006). Maternal exposure to LPS induces hypomyelination in the internal capsule and programmed cell death in the deep gray matter in newborn rats. *Pediatr. Res.* 59 428–433. 10.1203/01.pdr.0000199905.08848.55 16492984

[B582] Rousset C. I. Kassem J. Aubert A. Planchenault D. Gressens P. Chalon S. (2013). Maternal exposure to lipopolysaccharide leads to transient motor dysfunction in neonatal rats. *Dev. Neurosci.* 35 172–181. 10.1159/000346579 23445561

[B583] Ruiz C. C. U. Rosado-de-Castro P. H. Mendez-Otero R. Pimentel-Coelho P. M. (2017). “Mesenchymal stromal cell therapy for neonatal hypoxic-ischemic encephalopathy,” in *Neurological Regeneration*, ed. Van Pham P. (Berlin: Springer), 105–120.

[B584] Ruiz S. Diep D. Gore A. Panopoulos A. D. Montserrat N. Plongthongkum N. (2012). Identification of a specific reprogramming-associated epigenetic signature in human induced pluripotent stem cells. *Proc. Natl. Acad. Sci. U.S.A.* 109 16196–16201. 10.1073/pnas.1202352109 22991473 PMC3479609

[B585] Ruth V. J. Raivio K. O. (1988). Perinatal brain damage: predictive value of metabolic acidosis and the Apgar score. *BMJ* 297 24–27. 10.1136/bmj.297.6640.24 2457406 PMC1834158

[B586] Rutherford M. Counsell S. Allsop J. Boardman J. Kapellou O. Larkman D. (2004). Diffusion-weighted magnetic resonance imaging in term perinatal brain injury: a comparison with site of lesion and time from birth. *Pediatrics* 114 1004–1014. 10.1542/peds.2004-0222 15466098

[B587] Rutherford M. A. Pennock J. M. Counsell S. J. Mercuri E. Cowan F. M. Dubowitz L. M. (1998). Abnormal magnetic resonance signal in the internal capsule predicts poor neurodevelopmental outcome in infants with hypoxic-ischemic encephalopathy. *Pediatrics* 102(2 Pt 1), 323–328. 10.1542/peds.102.2.323 9685433

[B588] Saadani-Makki F. Kannan S. Lu X. Janisse J. Dawe E. Edwin S. (2008). Intrauterine administration of endotoxin leads to motor deficits in a rabbit model: a link between prenatal infection and cerebral palsy. *Am. J. Obstet. Gynecol.* 199 651.e1–651.e7 10.1016/j.ajog.2008.06.090 PMC2913549 18845289

[B589] Sabir H. Osredkar D. Maes E. Wood T. Thoresen M. (2016). Xenon combined with therapeutic hypothermia is not neuroprotective after severe hypoxia-ischemia in neonatal rats. *PLoS ONE* 11:e0156759 10.1371/journal.pone.0156759 PMC4890818 27253085

[B590] Sakurada O. Kennedy C. Jehle J. Brown J. D. Carbin G. L. Sokoloff L. (1978). Measurement of local cerebral blood flow with iodo [14C] antipyrine. *Am. J. Physiol.* 234 H59–H66. 623275 10.1152/ajpheart.1978.234.1.H59

[B591] Salhab W. A. Perlman J. M. Silver L. Broyles R. S. (2004a). Necrotizing enterocolitis and neurodevelopmental outcome in extremely low birth weight infants < 1000 g. *J. Perinatol.* 24 534–540. 15254558 10.1038/sj.jp.7211165

[B592] Salhab W. A. Wyckoff M. H. Laptook A. R. Perlman J. M. (2004b). Initial hypoglycemia and neonatal brain injury in term infants with severe fetal acidemia. *Pediatrics* 114 361–366. 15286217 10.1542/peds.114.2.361

[B593] Samaiya P. K. Krishnamurthy S. (2015). Characterization of mitochondrial bioenergetics in neonatal anoxic model of rats. *J. Bioenerg. Biomembr.* 47 217–222. 10.1007/s10863-015-9603-2 25637096

[B594] Sampath D. Shmueli D. White A. M. Raol Y. H. (2015). Flupirtine effectively prevents development of acute neonatal seizures in an animal model of global hypoxia. *Neurosci. Lett.* 607 46–51. 10.1016/j.neulet.2015.09.005 26365409 PMC4631640

[B595] Sanches E. Arteni N. Nicola F. Boisserand L. Willborn S. Netto C. (2013). Early hypoxia–ischemia causes hemisphere and sex-dependent cognitive impairment and histological damage. *Neuroscience* 237 208–215. 10.1016/j.neuroscience.2013.01.066 23395861

[B596] Sanches E. F. Arteni N. S. Spindler C. Moysés F. Siqueira I. R. Perry M. L. (2012). Effects of pre-and postnatal protein malnutrition in hypoxic–ischemic rats. *Brain Res.* 1438 85–92. 10.1016/j.brainres.2011.12.024 22244305

[B597] Santiago A. R. Baptista F. I. Santos P. F. Cristovao G. Ambrosio A. F. Cunha R. A. (2014). Role of microglia adenosine A(2A) receptors in retinal and brain neurodegenerative diseases. *Mediators Inflamm.* 2014:465694 10.1155/2014/465694 PMC4124703 25132733

[B598] Sato Y. Hayakawa M. Iwata O. Okumura A. Kato T. Hayakawa F. (2008). Delayed neurological signs following isolated parasagittal injury in asphyxia at term. *Eur. J. Paediatr. Neurol. Soc.* 12 359–365. 10.1016/j.ejpn.2007.10.003 18054507

[B599] Saunders N. R. Dreifuss J. J. Dziegielewska K. M. Johansson P. A. Habgood M. D. Mollgard K. (2014). The rights and wrongs of blood-brain barrier permeability studies: a walk through 100 years of history. *Front. Neurosci.* 8:404 10.3389/fnins.2014.00404 PMC4267212 25565938

[B600] Saunders N. R. Habgood M. D. Dziegielewska K. M. (1999). Barrier mechanisms in the brain, II. Immature brain. *Clin. Exp. Pharmacol. Physiol.* 26 85–91. 10.1046/j.1440-1681.1999.02987.x 10065326

[B601] Saunders N. R. Liddelow S. A. Dziegielewska K. M. (2012). Barrier mechanisms in the developing brain. *Front. Pharmacol.* 3:46 10.3389/fphar.2012.00046 PMC3314990 22479246

[B602] Savman K. Blennow M. Gustafson K. Tarkowski E. Hagberg H. (1998). Cytokine response in cerebrospinal fluid after birth asphyxia. *Pediatr. Res.* 43 746–751. 10.1203/00006450-199806000-00006 9621983

[B603] Schanne F. A. Kane A. B. Young E. E. Farber J. L. (1979). Calcium dependence of toxic cell death: a final common pathway. *Science* 206 700–702. 10.1126/science.386513 386513

[B604] Scheepens A. Wassink G. Piersma M. J. Van de Berg W. D. Blanco C. E. (2003). A delayed increase in hippocampal proliferation following global asphyxia in the neonatal rat. *Brain Res. Dev. Brain Res.* 142 67–76. 10.1016/S0165-3806(03)00032-4 12694945

[B605] Schrimpf S. P. Bleiker A. J. Brecevic L. Kozlov S. V. Berger P. Osterwalder T. (1997). Human neuroserpin (PI12): cDNA cloning and chromosomal localization to 3q26. *Genomics* 40 55–62. 10.1006/geno.1996.4514 9070919

[B606] Schroder M. Kaufman R. J. (2005). ER stress and the unfolded protein response. *Mutat. Res.* 569 29–63. 10.1016/j.mrfmmm.2004.06.056 15603751

[B607] Schulze C. Firth J. A. (1992). Interendothelial junctions during blood-brain barrier development in the rat: morphological changes at the level of individual tight junctional contacts. *Brain Res. Dev. Brain Res.* 69 85–95. 10.1016/0165-3806(92)90125-G 1424091

[B608] Seidler F. J. Slotkin T. A. (1990). Effects of acute hypoxia on neonatal rat brain: regionally selective, long-term alterations in catecholamine levels and turnover. *Brain Res. Bull.* 24 157–161. 10.1016/0361-9230(90)90200-J 2157528

[B609] Semple B. D. Blomgren K. Gimlin K. Ferriero D. M. Noble-Haeusslein L. J. (2013). Brain development in rodents and humans: identifying benchmarks of maturation and vulnerability to injury across species. *Progress Neurobiol.* 106 1–16. 10.1016/j.pneurobio.2013.04.001 PMC3737272 23583307

[B610] Shah D. K. Lavery S. Doyle L. W. Wong C. McDougall P. Inder T. E. (2006). Use of 2-channel bedside electroencephalogram monitoring in term-born encephalopathic infants related to cerebral injury defined by magnetic resonance imaging. *Pediatrics* 118 47–55. 10.1542/peds.2005-1294 16818548

[B611] Shah P. S. (2010). Hypothermia: a systematic review and meta-analysis of clinical trials. *Semin. Fetal Neonatal Med.* 15 238–246. 10.1016/j.siny.2010.02.003 20211588

[B612] Shah P. S. Beyene J. To T. Ohlsson A. Perlman M. (2006). Postasphyxial hypoxic-ischemic encephalopathy in neonates: outcome prediction rule within 4 hours of birth. *Arch. Pediatr. Adolesc. Med.* 160 729–736. 10.1001/archpedi.160.7.729 16818839

[B613] Shah P. S. Ohlsson A. Perlman M. (2007). Hypothermia to treat neonatal hypoxic ischemic encephalopathy: systematic review. *Arch. Pediatr. Adolesc. Med.* 161 951–958. 10.1001/archpedi.161.10.951 17909138

[B614] Shalak L. Perlman J. M. (2004). Hypoxic–ischemic brain injury in the term infant-current concepts. *Early Hum. Dev.* 80 125–141. 10.1016/j.earlhumdev.2004.06.003 15500993

[B615] Shankaran S. Laptook A. Wright L. L. Ehrenkranz R. A. Donovan E. F. Fanaroff A. A. (2002). Whole-body hypothermia for neonatal encephalopathy: animal observations as a basis for a randomized, controlled pilot study in term infants. *Pediatrics* 110 377–385. 10.1542/peds.110.2.377 12165594

[B616] Shankaran S. Laptook A. R. Ehrenkranz R. A. Tyson J. E. McDonald S. A. Donovan E. F. (2005). Whole-body hypothermia for neonates with hypoxic–ischemic encephalopathy. *N. Engl. J. Med.* 353 1574–1584. 10.1056/NEJMcps050929 16221780

[B617] Shankaran S. Pappas A. McDonald S. A. Vohr B. R. Hintz S. R. Yolton K. (2012). Childhood outcomes after hypothermia for neonatal encephalopathy. *N. Engl. J. Med.* 366 2085–2092. 10.1056/NEJMoa1112066 22646631 PMC3459579

[B618] Shankaran S. Woldt E. Koepke T. Bedard M. P. Nandyal R. (1991). Acute neonatal morbidity and long-term central nervous system sequelae of perinatal asphyxia in term infants. *Early Hum. Dev.* 25 135–148. 10.1016/0378-3782(91)90191-5 1713544

[B619] Sharma R. (2014). Prevention of Frey syndrome with superficial temporal fascia interpositioning: a retrospective study. *Int. J. Oral Maxillofac. Surg.* 43 413–417. 10.1016/j.ijom.2013.12.001 24461587

[B620] Sheldon R. A. Sedik C. Ferriero D. M. (1998). Strain-related brain injury in neonatal mice subjected to hypoxia–ischemia. *Brain Res.* 810 114–122. 10.1016/S0006-8993(98)00892-0 9813271

[B621] Shellhaas R. A. Soaita A. I. Clancy R. R. (2007). Sensitivity of amplitude-integrated electroencephalography for neonatal seizure detection. *Pediatrics* 120 770–777. 10.1542/peds.2007-0514 17908764

[B622] Shi L. Smith S. E. Malkova N. Tse D. Su Y. Patterson P. H. (2009). Activation of the maternal immune system alters cerebellar development in the offspring. *Brain Behav. Immun.* 23 116–123. 10.1016/j.bbi.2008.07.012 18755264 PMC2614890

[B623] Shimomura C. Ohta H. (1988). Behavioral abnormalities and seizure susceptibility in rat after neonatal anoxia. *Brain Dev.* 10 160–163. 10.1016/S0387-7604(88)80020-2 3407852

[B624] Sidhu R. S. Tuor U. I. Del Bigio M. R. (1997). Nuclear condensation and fragmentation following cerebral hypoxia-ischemia occurs more frequently in immature than older rats. *Neurosci. Lett.* 223 129–132. 10.1016/S0304-3940(97)13426-7 9089690

[B625] Simpson E. A. Murray L. Paukner A. Ferrari P. F. (2014). The mirror neuron system as revealed through neonatal imitation: presence from birth, predictive power and evidence of plasticity. *Philos. Trans. R. Soc. Lond. B Biol. Sci.* 369:20130289 10.1098/rstb.2013.0289 PMC4006187 24778381

[B626] Sinclair D. B. Campbell M. Byrne P. Prasertsom W. Robertson C. M. (1999). EEG and long-term outcome of term infants with neonatal hypoxic-ischemic encephalopathy. *Clin. Neurophysiol.* 110 655–659. 10.1016/S1388-2457(99)00010-3 10378734

[B627] Singh D. Kumar P. Majumdar S. Narang A. (2004). Effect of phenobarbital on free radicals in neonates with hypoxic ischemic encephalopathy–a randomized controlled trial. *J. Perinat. Med.* 32 278–281. 10.1515/JPM.2004.052 15188805

[B628] Slotkin T. Lappi S. McCook E. Lorber B. Seidler F. (1995). Loss of neonatal hypoxia tolerance after prenatal nicotine exposure: implications for sudden infant death syndrome. *Brain Res. Bull.* 38 69–75. 10.1016/0361-9230(95)00073-N 7552377

[B629] Slotkin T. A. Cowdery T. S. Orband L. Pachman S. Whitmore W. L. (1986). Effects of neonatal hypoxia on brain development in the rat: immediate and long-term biochemical alterations in discrete regions. *Brain Res.* 374 63–74. 10.1016/0006-8993(86)90395-1 3719331

[B630] Smith P. L. Hagberg H. Naylor A. S. Mallard C. (2014). Neonatal peripheral immune challenge activates microglia and inhibits neurogenesis in the developing murine hippocampus. *Dev. Neurosci.* 36 119–131. 10.1159/000359950 24642725

[B631] Soares D. P. Law M. (2009). Magnetic resonance spectroscopy of the brain: review of metabolites and clinical applications. *Clin. Radiol.* 64 12–21. 10.1016/j.crad.2008.07.002 19070693

[B632] Sokoloff L. Reivich M. Kennedy C. Des Rosiers M. Patlak C. S. Pettigrew K. E. A. (1977). The [14C] deoxyglucose method for the measurement of local cerebral glucose utilization: theory, procedure, and normal values in the conscious and anesthetized albino rat1. *J. Neurochem.* 28 897–916. 10.1111/j.1471-4159.1977.tb10649.x 864466

[B633] Soul J. S. Robertson R. L. Tzika A. A. du Plessis A. J. Volpe J. J. (2001). Time course of changes in diffusion-weighted magnetic resonance imaging in a case of neonatal encephalopathy with defined onset and duration of hypoxic-ischemic insult. *Pediatrics* 108 1211–1214. 10.1542/peds.108.5.1211 11694704

[B634] Speiser Z. Katzir O. Rehavi M. Zabarski T. Cohen S. (1998). Sparing by rasagiline (TVP-1012) of cholinergic functions and behavior in the postnatal anoxia rat. *Pharmacol. Biochem. Behav.* 60 387–393. 10.1016/S0091-3057(97)00603-5 9632221

[B635] Speiser Z. Uziel J. Defrin-Assa R. Gitter S. Urca G. (1991). Different behavioral deficits are induced by anoxia/hypoxia in neonatal and senescent rats: blockade by MK-801. *Behav. Brain Res.* 42 181–186. 10.1016/S0166-4328(05)80009-9 2059331

[B636] Spraggins Y. Seidler F. J. Slotkin T. A. (1994). Cocaine exacerbates hypoxia-lnduced cell damage in the developing brain: effects on ornithine decarboxylase activity and protein synthesis. *Neonatology* 66 254–266. 10.1159/000244115 7873692

[B637] Srinivasakumar P. Zempel J. Wallendorf M. Lawrence R. Inder T. Mathur A. (2013). Therapeutic hypothermia in neonatal hypoxic ischemic encephalopathy: electrographic seizures and magnetic resonance imaging evidence of injury. *J. Pediatr.* 163 465–470. 10.1016/j.jpeds.2013.01.041 23452588

[B638] Staley K. J. Soldo B. L. Proctor W. R. (1995). Ionic mechanisms of neuronal excitation by inhibitory GABAA receptors. *Science* 269 977–981. 10.1126/science.7638623 7638623

[B639] Starkov A. A. Chinopoulos C. Fiskum G. (2004). Mitochondrial calcium and oxidative stress as mediators of ischemic brain injury. *Cell Calcium* 36 257–264. 10.1016/j.ceca.2004.02.012 15261481

[B640] Steinman K. J. Gorno-Tempini M. L. Glidden D. V. Kramer J. H. Miller S. P. Barkovich A. J. (2009). Neonatal watershed brain injury on magnetic resonance imaging correlates with verbal IQ at 4 years. *Pediatrics* 123 1025–1030. 10.1542/peds.2008-1203 19255035 PMC2718837

[B641] Stewart P. A. Hayakawa K. (1994). Early ultrastructural changes in blood-brain barrier vessels of the rat embryo. *Brain Res. Dev. Brain Res.* 78 25–34. 10.1016/0165-3806(94)90005-1 8004771

[B642] Stiller R. von Mering R. Konig V. Huch A. Huch R. (2002). How well does reflectance pulse oximetry reflect intrapartum fetal acidosis? *Am. J. Obst. Gynecol.* 186 1351–1357. 10.1067/mob.2002.122411 12066121

[B643] Stoll B. J. Hansen N. I. Adams-Chapman I. Fanaroff A. A. Hintz S. R. Vohr B. (2004). Neurodevelopmental and growth impairment among extremely low-birth-weight infants with neonatal infection. *JAMA* 292 2357–2365. 10.1001/jama.292.19.2357 15547163

[B644] Stolp H. B. Liddelow S. A. Saunders N. R. (2016). Editorial: ontogeny and phylogeny of brain barrier mechanisms. *Front. Neurosci.* 10:41 10.3389/fnins.2016.00041 PMC4754436 26909020

[B645] Stonestreet B. S. Burgess G. H. Cserr H. F. (1992). Blood-brain barrier integrity and brain water and electrolytes during hypoxia/hypercapnia and hypotension in newborn piglets. *Brain Res.* 590 263–270. 10.1016/0006-8993(92)91104-M 1422834

[B646] Stridh L. Mottahedin A. Johansson M. E. Valdez R. C. Northington F. Wang X. (2013). Toll-like receptor-3 activation increases the vulnerability of the neonatal brain to hypoxia-ischemia. *J. Neurosci.* 33 12041–12051. 10.1523/JNEUROSCI.0673-13.2013 23864690 PMC3713735

[B647] Stritt C. Stern S. Harting K. Manke T. Sinske D. Schwarz H. (2009). Paracrine control of oligodendrocyte differentiation by SRF-directed neuronal gene expression. *Nat. Neurosci.* 12 418–427. 10.1038/nn.2280 19270689

[B648] Stroemer R. P. Rothwell N. J. (1998). Exacerbation of ischemic brain damage by localized striatal injection of interleukin-1beta in the rat. *J. Cereb. Blood Flow Metab.* 18 833–839. 10.1097/00004647-199808000-00003 9701344

[B649] Strunk T. Hartel C. Temming P. Matzke N. Zimmer J. Schultz C. (2008). Erythropoietin inhibits cytokine production of neonatal and adult leukocytes. *Acta Paediatr.* 97 16–20. 10.1111/j.1651-2227.2007.00560.x 18052997

[B650] Strunk T. Inder T. Wang X. Burgner D. Mallard C. Levy O. (2014). Infection-induced inflammation and cerebral injury in preterm infants. *Lancet Infect. Dis.* 14 751–762. 10.1016/S1473-3099(14)70710-8 24877996 PMC4125363

[B651] Suarez-Sola M. L. Gonzalez-Delgado F. J. Pueyo-Morlans M. Medina-Bolivar O. C. Hernandez-Acosta N. C. Gonzalez-Gomez M. (2009). Neurons in the white matter of the adult human neocortex. *Front. Neuroanat.* 3:7 10.3389/neuro.05.007.2009 PMC2697018 19543540

[B652] Svedin P. Hagberg H. Savman K. Zhu C. Mallard C. (2007). Matrix metalloproteinase-9 gene knock-out protects the immature brain after cerebral hypoxia-ischemia. *J. Neurosci.* 27 1511–1518. 10.1523/JNEUROSCI.4391-06.2007 17301159 PMC6673738

[B653] Svedin P. Kjellmer I. Welin A. K. Blad S. Mallard C. (2005). Maturational effects of lipopolysaccharide on white-matter injury in fetal sheep. *J. Child Neurol.* 20 960–964. 10.1177/08830738050200120501 16417842

[B654] Tagin M. A. Woolcott C. G. Vincer M. J. Whyte R. K. Stinson D. A. (2012). Hypothermia for neonatal hypoxic ischemic encephalopathy: an updated systematic review and meta-analysis. *Arch. Pediatr. Adolesc. Med.* 166 558–566. 10.1001/archpediatrics.2011.1772 22312166

[B655] Tahraoui S. L. Marret S. Bodenant C. Leroux P. Dommergues M. A. Evrard P. (2001). Central role of microglia in neonatal excitotoxic lesions of the murine periventricular white matter. *Brain Pathol.* 11 56–71. 10.1111/j.1750-3639.2001.tb00381.x 11145204 PMC8098534

[B656] Tajiri N. Kaneko Y. Shinozuka K. Ishikawa H. Yankee E. McGrogan M. (2013). Stem cell recruitment of newly formed host cells via a successful seduction? Filling the gap between neurogenic niche and injured brain site. *PLoS ONE* 8:e74857 10.1371/journal.pone.0074857 PMC3762783 24023965

[B657] Takada S. dos Santos Haemmerle C. Motta-Teixeira L. Machado-Nils A. Lee V. Takase L. (2015). Neonatal anoxia in rats: hippocampal cellular and subcellular changes related to cell death and spatial memory. *Neuroscience* 284 247–259. 10.1016/j.neuroscience.2014.08.054 25305666

[B658] Takada S. H. Motta-Teixeira L. C. Machado-Nils A. V. Lee V. Y. Sampaio C. A. Polli R. S. (2016). Impact of neonatal anoxia on adult rat hippocampal volume, neurogenesis and behavior. *Behav. Brain Res.* 296 331–338. 10.1016/j.bbr.2015.08.039 26416672

[B659] Takada T. Takata K. Ashihara E. (2016). Inhibition of monocarboxylate transporter 1 suppresses the proliferation of glioblastoma stem cells. *J. Physiol. Sci.* 66 387–396. 10.1007/s12576-016-0435-6 26902636 PMC10717967

[B660] Takahashi S. Tanaka H. Oki J. (1999). Development of spinal motoneurons in rats after a neonatal hypoxic insult. *Pediatr. Neurol.* 21 715–720. 10.1016/S0887-8994(99)00080-6 10580883

[B661] Takao K. Miyakawa T. (2015). Genomic responses in mouse models greatly mimic human inflammatory diseases. *Proc. Natl. Acad. Sci. U.S.A.* 112 1167–1172. 10.1073/pnas.1401965111 25092317 PMC4313832

[B662] Takeoka M. Soman T. B. Yoshii A. Caviness V. S. Jr. Gonzalez R. G. Grant P. E. (2002). Diffusion-weighted images in neonatal cerebral hypoxic-ischemic injury. *Pediatr. Neurol.* 26 274–281. 10.1016/S0887-8994(01)00403-9 11992754

[B663] Talos D. M. Fishman R. E. Park H. Folkerth R. D. Follett P. L. Volpe J. J. (2006). Developmental regulation of alpha-amino-3-hydroxy-5-methyl-4-isoxazole-propionic acid receptor subunit expression in forebrain and relationship to regional susceptibility to hypoxic/ischemic injury. I. Rodent cerebral white matter and cortex. *J. Comp. Neurol.* 497 42–60. 10.1002/cne.20972 16680782 PMC4313670

[B664] Tanaka H. Takahashi S. Miyamoto A. Oki J. Cho K. Okuno A. (1995). Effects of neonatal hypoxia on brainstem cholinergic neurons-pedunculopontine nucleus and laterodorsal tegmental nucleus. *Brain Dev.* 17 264–270. 10.1016/0387-7604(95)00043-B 7503389

[B665] Tang Y. Pacary E. Freret T. Divoux D. Petit E. Schumann-Bard P. (2006). Effect of hypoxic preconditioning on brain genomic response before and following ischemia in the adult mouse: identification of potential neuroprotective candidates for stroke. *Neurobiol. Dis.* 21 18–28. 10.1016/j.nbd.2005.06.002 16040250

[B666] Tao F. Lu S. D. Zhang L. M. Huang Y. L. Sun F. Y. (2001). Role of excitatory amino acid transporter 1 in neonatal rat neuronal damage induced by hypoxia-ischemia. *Neuroscience* 102 503–513. 10.1016/S0306-4522(00)00485-1 11226689

[B667] Teesalu T. Kulla A. Simisker A. Sirén V. Lawrence D. A. Asser T. (2004). Tissue plasminogen activator and neuroserpin are widely expressed in the human central nervous system. *Thromb. Haemost.* 92 358–368. 10.1160/th02-12-0310 15269833

[B668] Thompson C. Puterman A. Linley L. Hann F. Elst C. V. D. Molteno C. (1997). The value of a scoring system for hypoxic ischaemic encephalopathy in predicting neurodevelopmental outcome. *Acta Paediatr.* 86 757–761. 10.1111/j.1651-2227.1997.tb08581.x 9240886

[B669] Thompson D. K. Warfield S. K. Carlin J. B. Pavlovic M. Wang H. X. Bear M. (2007). Perinatal risk factors altering regional brain structure in the preterm infant. *Brain* 130(Pt 3), 667–677. 10.1093/brain/awl277 17008333

[B670] Thoresen M. Hobbs C. E. Wood T. Chakkarapani E. Dingley J. (2009). Cooling combined with immediate or delayed xenon inhalation provides equivalent long-term neuroprotection after neonatal hypoxia-ischemia. *J. Cereb. Blood Flow Metab.* 29 707–714. 10.1038/jcbfm.2008.163 19142190

[B671] Ting P. Yamaguchi S. Bacher J. D. Killens R. H. Myers R. E. (1983). Hypoxic-ischemic cerebral necrosis in midgestational sheep fetuses: physiopathologic correlations. *Exp. Neurol.* 80 227–245. 10.1016/0014-4886(83)90019-5 6403369

[B672] Tjärnlund-Wolf A. Olsson S. Jood K. Blomstrand C. Jern C. (2011). No evidence for an association between genetic variation at the SERPINI1 locus and ischemic stroke. *J. Neurol.* 258 1885 10.1007/s00415-011-6022-0 21487809

[B673] Toet M. C. Hellstrom-Westas L. Groenendaal F. Eken P. de Vries L. S. (1999). Amplitude integrated EEG 3 and 6 hours after birth in full term neonates with hypoxic-ischaemic encephalopathy. *Arch. Dis. Child. Fetal Neonatal Ed.* 81 F19–F23. 10.1136/fn.81.1.f19 10375357 PMC1720950

[B674] Toet M. C. van der Meij W. de Vries L. S. Uiterwaal C. S. van Huffelen K. C. (2002). Comparison between simultaneously recorded amplitude integrated electroencephalogram (cerebral function monitor) and standard electroencephalogram in neonates. *Pediatrics* 109 772–779. 10.1542/peds.109.5.772 11986435

[B675] Tolner E. A. Hochman D. W. Hassinen P. Otahal J. Gaily E. Haglund M. M. (2011). Five percent CO(2) is a potent, fast-acting inhalation anticonvulsant. *Epilepsia* 52 104–114. 10.1111/j.1528-1167.2010.02731.x 20887367 PMC3017646

[B676] Tomimatsu T. Fukuda H. Endoh M. Mu J. Watanabe N. Kohzuki M. (2002). Effects of neonatal hypoxic–ischemic brain injury on skilled motor tasks and brainstem function in adult rats. *Brain Res.* 926 108–117. 10.1016/S0006-8993(01)03311-X 11814412

[B677] Toso L. Poggi S. Park J. Einat H. Roberson R. Dunlap V. (2005). Inflammatory-mediated model of cerebral palsy with developmental sequelae. *Am. J. Obstet. Gynecol.* 193(3 Pt 2), 933–941. 10.1016/j.ajog.2005.05.072 16157090

[B678] Towfighi J. Yager J. Housman C. Vannucci R. (1991). Neuropathology of remote hypoxic-ischemic damage in the immature rat. *Acta Neuropathol.* 81 578–587. 10.1007/BF00310141 1858486

[B679] Towfighi J. Zec N. Yager J. Housman C. Vannucci R. C. (1995). Temporal evolution of neuropathologic changes in an immature rat model of cerebral hypoxia: a light microscopic study. *Acta Neuropathol.* 90 375–386. 10.1007/BF00315011 8546028

[B680] Tran T. V. Weisman L. E. (2004). Dexamethasone effects on group B streptococcal infection in newborn rats. *Pediatr. Infect. Dis. J.* 23 47–52. 10.1097/01.inf.0000105107.76541.ee 14743046

[B681] Tremblay E. Roisin M. P. Represa A. Charriaut-Marlangue C. Ben-Ari Y. (1988). Transient increased density of NMDA binding sites in the developing rat hippocampus. *Brain Res.* 461 393–396. 10.1016/0006-8993(88)90275-2 2902905

[B682] Triulzi F. Parazzini C. Righini A. (2006). Patterns of damage in the mature neonatal brain. *Pediatr. Radiol.* 36 608–620. 10.1007/s00247-006-0203-5 16770665

[B683] Trommer B. L. Groothuis D. R. Pasternak J. F. (1987). Quantitative analysis of cerebral vessels in the newborn puppy: the structure of germinal matrix vessels may predispose to hemorrhage. *Pediatr. Res.* 22 23–28. 10.1203/00006450-198707000-00007 3627867

[B684] Tu Y. F. Tsai Y. S. Wang L. W. Wu H. C. Huang C. C. Ho C. J. (2011). Overweight worsens apoptosis, neuroinflammation and blood-brain barrier damage after hypoxic ischemia in neonatal brain through JNK hyperactivation. *J. Neuroinflammation* 8:40 10.1186/1742-2094-8-40 PMC3090337 21518436

[B685] Tuor U. Chumas P. Del Bigio M. (1995). Prevention of hypoxic-ischemic damage with dexamethasone is dependent on age and not influenced by fasting. *Exp. Neurol.* 132 116–122. 10.1016/0014-4886(95)90065-9 7720820

[B686] Tuor U. I. Grewal D. (1994). Autoregulation of cerebral blood flow: influence of local brain development and postnatal age. *Am. J. Physiol.* 267(6 Pt 2), H2220–H2228. 7810721 10.1152/ajpheart.1994.267.6.H2220

[B687] Uehara T. Tabuchi M. Mori E. (1999). Risk factors for silent cerebral infarcts in subcortical white matter and basal ganglia. *Stroke* 30 378–382. 10.1161/01.STR.30.2.378 9933274

[B688] Ujhazy E. Schmidtova M. Dubovicky M. Navarova J. Brucknerova I. Mach M. (2006). Neurobehavioural changes in rats after neonatal anoxia: effect of antioxidant stobadine pretreatment. *Neuro Endocrinol. Lett.* 27(Suppl. 2), 82–85. 17159786

[B689] Valiente M. Obenauf A. C. Jin X. Chen Q. Zhang X. H. Lee D. J. (2014). Serpins promote cancer cell survival and vascular co-option in brain metastasis. *Cell* 156 1002–1016. 10.1016/j.cell.2014.01.040 24581498 PMC3988473

[B690] van der Worp H. B. Sena E. S. Donnan G. A. Howells D. W. Macleod M. R. (2007). Hypothermia in animal models of acute ischaemic stroke: a systematic review and meta-analysis. *Brain* 130(Pt 12), 3063–3074. 10.1093/brain/awm083 17478443

[B691] van Laerhoven H. de Haan T. R. Offringa M. Post B. van der Lee J. H. (2013). Prognostic tests in term neonates with hypoxic-ischemic encephalopathy: a systematic review. *Pediatrics* 131 88–98. 10.1542/peds.2012-1297 23248219

[B692] Vandenberg R. J. Ryan R. M. (2013). Mechanisms of glutamate transport. *Physiol. Rev.* 93 1621–1657. 10.1152/physrev.00007.2013 24137018

[B693] Vannucci R. Connor J. Mauger D. Palmer C. Smith M. Towfighi J. (1999). Rat model of perinatal hypoxic-ischemic brain damage. *J. Neurosci. Res.* 55 158–163. 10.1002/(SICI)1097-4547(19990115)55:2<158::AID-JNR3>3.0.CO;2-1 9972818

[B694] Vannucci R. C. Brucklacher R. M. Vannucci S. J. (2005). Glycolysis and perinatal hypoxic-ischemic brain damage. *Dev. Neurosci.* 27 185–190. 10.1159/000085991 16046853

[B695] Vannucci R. C. Christensen M. A. Stein D. T. (1989). Regional cerebral glucose utilization in the immature rat: effect of hypoxia-ischemia. *Pediatr. Res.* 26 208–214. 10.1203/00006450-198909000-00011 2587122

[B696] Vannucci R. C. Christensen M. A. Yager J. Y. (1993). Nature, time-course, and extent of cerebral edema in perinatal hypoxic-ischemic brain damage. *Pediatr. Neurol.* 9 29–34. 10.1016/0887-8994(93)90006-X 8452596

[B697] Vannucci R. C. Duffy T. E. (1976). Carbohydrate metabolism in fetal and neonatal rat brain during anoxia and recovery. *Am. J. Physiol.* 230 1269–1275. 1275068 10.1152/ajplegacy.1976.230.5.1269

[B698] Vannucci R. C. Lyons D. T. Vasta F. (1988). Regional cerebral blood flow during hypoxia-ischemia in immature rats. *Stroke* 19 245–250. 10.1161/01.STR.19.2.245 3344541

[B699] Vannucci R. C. Vannucci S. J. (2005). Perinatal hypoxic-ischemic brain damage: evolution of an animal model. *Dev. Neurosci.* 27 81–86. 10.1159/000085978 16046840

[B700] Vannucci R. C. Yager J. Y. (1992). Glucose, lactic acid, and perinatal hypoxic-ischemic brain damage. *Pediatr. Neurol.* 8 3–12. 10.1016/0887-8994(92)90045-Z 1558572

[B701] Vannucci S. J. Hagberg H. (2004). Hypoxia-ischemia in the immature brain. *J. Exp. Biol.* 207(Pt 18), 3149–3154. 10.1242/jeb.01064 15299036

[B702] Vannucci S. J. Vannucci R. C. (1980). Glycogen metabolism in neonatal rat brain during anoxia and recovery. *J. Neurochem.* 34 1100–1105. 10.1111/j.1471-4159.1980.tb09946.x 7373299

[B703] Vargas-Origel A. Espinosa-Garcia J. O. Muniz-Quezada E. Vargas-Nieto M. A. Aguilar-Garcia G. (2004). Prevention of hypoxic-ischemic encephalopathy with high-dose, early phenobarbital therapy. *Gac. Med. Mex.* 140 147–153. 15162947

[B704] Venerosi A. Cutuli D. Chiarotti F. Calamandrei G. (2006). C-section birth per se or followed by acute global asphyxia altered emotional behaviour in neonate and adult rats. *Behav. Brain Res.* 168 56–63. 10.1016/j.bbr.2005.10.010 16310869

[B705] Vento M. Asensi M. Sastre J. Garcia-Sala F. Pallardo F. V. Vina J. (2001a). Resuscitation with room air instead of 100% oxygen prevents oxidative stress in moderately asphyxiated term neonates. *Pediatrics* 107 642–647. 11335737 10.1542/peds.107.4.642

[B706] Vento M. Asensi M. Sastre J. Garcia-Sala F. Vina J. (2001b). Six years of experience with the use of room air for the resuscitation of asphyxiated newly born term infants. *Biol. Neonate* 79 261–267. 11275663 10.1159/000047103

[B707] Verma P. K. Panerai R. B. Rennie J. M. Evans D. H. (2000). Grading of cerebral autoregulation in preterm and term neonates. *Pediatr. Neurol.* 23 236–242. 10.1016/S0887-8994(00)00184-3 11033287

[B708] Vermeulen R. J. Fetter W. P. Hendrikx L. Van Schie P. E. van der Knaap M. S. Barkhof F. (2003). Diffusion-weighted MRI in severe neonatal hypoxic ischaemia: the white cerebrum. *Neuropediatrics* 34 72–76. 10.1055/s-2003-39599 12776227

[B709] Vincer M. J. Allen A. C. Joseph K. S. Stinson D. A. Scott H. Wood E. (2006). Increasing prevalence of cerebral palsy among very preterm infants: a population-based study. *Pediatrics* 118 e1621–e1626. 10.1542/peds.2006-1522 17074842

[B710] Vohr B. R. Wright L. L. Dusick A. M. Mele L. Verter J. Steichen J. J. (2000). Neurodevelopmental and functional outcomes of extremely low birth weight infants in the National Institute of Child Health and Human Development Neonatal Research Network, 1993-1994. *Pediatrics* 105 1216–1226. 10.1542/peds.105.6.1216 10835060

[B711] Volpe A. R. Petrone M. E. De Vizio W. Davies R. M. Proskin H. M. (1996). A review of plaque, gingivitis, calculus and caries clinical efficacy studies with a fluoride dentifrice containing triclosan and PVM/MA copolymer. *J. Clin. Den.* 7(Suppl.), S1–S14. 9238866

[B712] Volpe J. (ed.). (1995). “Hypoxic-ischemic encephalopathy: neuropathology and pathogenesis,” in *Neurology of the Newborn*, (London: W B Saunders), 279–313.

[B713] Volpe J. (2001). *Hypoxic-Ischemic Encephalopathy in Neurology of the Newborn.* Philadelphia, PA: WB Saunders.

[B714] Volpe J. J. (1977). Neonatal seizures. *Clin. Perinatol.* 4 43–63. 322917

[B715] Volpe J. J. (1989). Intraventricular hemorrhage in the premature infant–current concepts. Part I. *Ann. Neurol.* 25 3–11. 10.1002/ana.410250103 2913926

[B716] Volpe J. J. (2008). *Neurology of the Newborn.* Philadelphia, PA: Elsevier Health Sciences.

[B717] Volpe J. J. (2009). The encephalopathy of prematurity–brain injury and impaired brain development inextricably intertwined. *Semin. Pediatr. Neurol.* 16 167–178. 10.1016/j.spen.2009.09.005 19945651 PMC2799246

[B718] Volpe J. J. (2012). Neonatal encephalopathy: an inadequate term for hypoxic–ischemic encephalopathy. *Ann. Neurol.* 72 156–166. 10.1002/ana.23647 22926849

[B719] Waddell J. Hanscom M. Shalon Edwards N. McKenna M. C. McCarthy M. M. (2016). Sex differences in cell genesis, hippocampal volume and behavioral outcomes in a rat model of neonatal HI. *Exp. Neurol.* 275(Pt 2), 285–295. 10.1016/j.expneurol.2015.09.003 26376217 PMC4688089

[B720] Walsh B. H. Murray D. M. Boylan G. B. (2011). The use of conventional EEG for the assessment of hypoxic ischaemic encephalopathy in the newborn: a review. *Clin. Neurophysiol.* 122 1284–1294. 10.1016/j.clinph.2011.03.032 21550844

[B721] Wang C. T. Lin H. J. Cheng B. C. Lin M. T. Chang C. P. (2015). Attenuating systemic inflammatory markers in simulated high-altitude exposure by heat shock protein 70-mediated hypobaric hypoxia preconditioning in rats. *J. Formos. Med. Assoc.* 114 328–338. 10.1016/j.jfma.2012.11.015 25839766

[B722] Wang L. Goldstein F. C. Veledar E. Levey A. I. Lah J. J. Meltzer C. C. (2009). Alterations in cortical thickness and white matter integrity in mild cognitive impairment measured by whole-brain cortical thickness mapping and diffusion tensor imaging. *Am. J. Neuroradiol.* 30 893–899. 10.3174/ajnr.A1484 19279272 PMC2901819

[B723] Wang L. S. Zhou J. Shao X. M. Tang X. C. (2002). Huperzine A attenuates cognitive deficits and brain injury in neonatal rats after hypoxia–ischemia. *Brain Res.* 949 162–170. 10.1016/S0006-8993(02)02977-3 12213312

[B724] Wang S. Zhang X. Q. Song C. G. Xiao T. Zhao M. Zhu G. (2015). In vivo effects of bumetanide at brain concentrations incompatible with NKCC1 inhibition on newborn DGC structure and spontaneous EEG seizures following hypoxia-induced neonatal seizures. *Neurosci.* 286 203–215. 10.1016/j.neuroscience.2014.11.031 25463517

[B725] Wang X. Hagberg H. Nie C. Zhu C. Ikeda T. Mallard C. (2007a). Dual role of intrauterine immune challenge on neonatal and adult brain vulnerability to hypoxia-ischemia. *J. Neuropathol. Exp. Neurol.* 66 552–561. 17549015 10.1097/01.jnen.0000263870.91811.6f

[B726] Wang X. Hagberg H. Zhu C. Jacobsson B. Mallard C. (2007b). Effects of intrauterine inflammation on the developing mouse brain. *Brain Res.* 1144 180–185. 10.1016/j.brainres.2007.01.083 17320062

[B727] Wang X. Rousset C. I. Hagberg H. Mallard C. (2006). Lipopolysaccharide-induced inflammation and perinatal brain injury. *Semin. Fetal Neonatal Med.* 11 343–353. 10.1016/j.siny.2006.04.002 16793357

[B728] Wang X. Zhu C. Qiu L. Hagberg H. Sandberg M. Blomgren K. (2003). Activation of ERK1/2 after neonatal rat cerebral hypoxia-ischaemia. *J. Neurochem.* 86 351–362. 10.1046/j.1471-4159.2003.01838.x 12871576

[B729] Wannier-Morino P. Rager G. Sonderegger P. Grabs D. (2003). Expression of neuroserpin in the visual cortex of the mouse during the developmental critical period. *Eur. J. Neurosci.* 17 1853–1860. 10.1046/j.1460-9568.2003.02628.x 12752785

[B730] Ward P. Counsell S. Allsop J. Cowan F. Shen Y. Edwards D. (2006). Reduced fractional anisotropy on diffusion tensor magnetic resonance imaging after hypoxic-ischemic encephalopathy. *Pediatrics* 117 e619–e630. 10.1542/peds.2005-0545 16510613

[B731] Wasterlain C. G. (1979). Does anoxemia play a role in the effects of neonatal seizures on brain growth? An experimental study in the rat. *Eur. Neurol.* 18 222–229. 10.1159/000115080 488139

[B732] Watanabe S. Fukuda T. Ueko S. (1980). Changes in electroencephalogram of the rat following olfactory bulbectomy. *Tohoku J. Exp. Med.* 130 41–48. 10.1620/tjem.130.41 7189303

[B733] Weeke L. C. Boylan G. B. Pressler R. M. Hallberg B. Blennow M. Toet M. C. (2016). Role of EEG background activity, seizure burden and MRI in predicting neurodevelopmental outcome in full-term infants with hypoxic-ischaemic encephalopathy in the era of therapeutic hypothermia. *Eur. J. Paediatr. Neurol.* 20 855–864. 10.1016/j.ejpn.2016.06.003 27370316

[B734] Welsh F. A. O’Connor M. J. Marcy V. R. Spatacco A. J. Johns R. L. (1982). Factors limiting regeneration of ATP following temporary ischemia in cat brain. *Stroke* 13 234–242. 10.1161/01.STR.13.2.234 7064195

[B735] Westphal O. (1965). Bacterial lipopolysaccharide-extraction with phenol water and further application of procedure. *Methods Carbohydr. Chem.* 1 83–91.

[B736] White A. R. Cappai R. (2003). Neurotoxicity from glutathione depletion is dependent on extracellular trace copper. *J. Neurosci. Res.* 71 889–897. 10.1002/jnr.10537 12605416

[B737] Wigglesworth J. S. Pape K. E. (1978). An integrated model for haemorrhagic and ischaemic lesions in the newborn brain. *Early Hum. Dev.* 2 179–199. 10.1016/0378-3782(78)90010-5 569048

[B738] Williams C. E. Gunn A. J. Mallard C. Gluckman P. D. (1992). Outcome after ischemia in the developing sheep brain: an electroencephalographic and histological study. *Ann. Neurol.* 31 14–21. 10.1002/ana.410310104 1543346

[B739] Wilson-Costello D. Friedman H. Minich N. Siner B. Taylor G. Schluchter M. (2007). Improved neurodevelopmental outcomes for extremely low birth weight infants in 2000-2002. *Pediatrics* 119 37–45. 10.1542/peds.2006-1416 17200269

[B740] Woo T. U. Beale J. M. Finlay B. L. (1991). Dual fate of subplate neurons in a rodent. *Cereb. Cortex* 1 433–443. 10.1093/cercor/1.5.433 1822751

[B741] Wood P. L. (1995). Microglia as a unique cellular target in the treatment of stroke: potential neurotoxic mediators produced by activated microglia. *Neurol. Res.* 17 242–248. 10.1080/01616412.1995.11740321 7477737

[B742] Wu J. Echeverry R. Guzman J. Yepes M. (2010). Neuroserpin protects neurons from ischemia-induced plasmin-mediated cell death independently of tissue-type plasminogen activator inhibition. *Am. J. Pathol.* 177 2576–2584. 10.2353/ajpath.2010.100466 20864675 PMC2966813

[B743] Wu J. Kaufman R. J. (2006). From acute ER stress to physiological roles of the Unfolded Protein Response. *Cell Death Differ.* 13 374–384. 10.1038/sj.cdd.4401840 16397578

[B744] Wyatt J. S. Edwards A. D. Azzopardi D. Reynolds E. O. (1989). Magnetic resonance and near infrared spectroscopy for investigation of perinatal hypoxic-ischaemic brain injury. *Arch. Dis. Child.* 64 953–963. 10.1136/adc.64.7_Spec_No.953 2673061 PMC1590085

[B745] Xiong M. Li J. Ma S. Yang Y. Zhou W. (2013). Effects of hypothermia on oligodendrocyte precursor cell proliferation, differentiation and maturation following hypoxia ischemia in vivo and in vitro. *Exp. Neurol.* 247 720–729. 10.1016/j.expneurol.2013.03.015 23524193

[B746] Yager J. Towfighi J. Vannucci R. (1993). Influence of mild hypothermia on hypoxic-ischemic brain damage in the immature rat. *Pediatr. Res.* 34 525–529. 10.1203/00006450-199310000-00029 8255688

[B747] Yager J. Y. Ashwal S. (2009). Animal models of perinatal hypoxic-ischemic brain damage. *Pediatr. Neurol.* 40 156–167. 10.1016/j.pediatrneurol.2008.10.025 19218028

[B748] Yager J. Y. Brucklacher R. M. Vannucci R. C. (1991). Cerebral oxidative metabolism and redox state during hypoxia-ischemia and early recovery in immature rats. *Am. J. Physiol.* 261(4 Pt 2), H1102–H1108. 1928392 10.1152/ajpheart.1991.261.4.H1102

[B749] Yager J. Y. Brucklacher R. M. Vannucci R. C. (1996). Paradoxical mitochondrial oxidation in perinatal hypoxic-ischemic brain damage. *Brain Res.* 712 230–238. 10.1016/0006-8993(95)01423-3 8814897

[B750] Yager J. Y. Heitjan D. F. Towfighi J. Vannucci R. C. (1992). Effect of insulin-induced and fasting hypoglycemia on perinatal hypoxic-ischemic brain damage. *Pediatr. Res.* 31 138–142. 10.1203/00006450-199202000-00009 1542541

[B751] Yamamoto H. Inaba S. Nishiura Y. Kishi F. Kawakami Y. (1985). Acute inhalation of cigarette smoke augments hypoxic chemosensitivity in humans. *J. Appl. Physiol.* 58 717–723. 3920193 10.1152/jappl.1985.58.3.717

[B752] Yamamoto H. Kato T. (1986). The effect of neonatal anoxia on brain cholecystokinin-8-like immunoreactivity and monoamine levels of mature rats. *Brain Res.* 391 285–288. 10.1016/0165-3806(86)90294-4 2421854

[B753] Yamamoto K. Yamamoto Y. Fujimiya T. Okae M. (1986). Effects of ethanol on the blood gas and acid-base state in hypoxic rats. *Jpn. J. Legal Med.* 40 357–360. 3099038

[B754] Yang D. Kuan C. (2015). Anti-tissue Plasminogen Activator (tPA) as an Effective Therapy of Neonatal Hypoxia–Ischemia with and without Inflammation. *CNS Neurosci. Ther.* 21 367–373. 10.1111/cns.12365 25475942 PMC4376575

[B755] Yang D. Li S. Y. Yeung C. M. Chang R. C. C. So K. F. Wong D. (2012). *Lycium barbarum* extracts protect the brain from blood-brain barrier disruption and cerebral edema in experimental stroke. *PLoS ONE* 7:e33596 10.1371/journal.pone.0033596 PMC3306421 22438957

[B756] Yang D. Sun Y. Y. Lin X. Baumann J. M. Warnock M. Lawrence D. A. (2013a). Taming neonatal hypoxic-ischemic brain injury by intranasal delivery of plasminogen activator inhibitor-1. *Stroke* 44 2623–2627. 10.1161/STROKEAHA.113.001233 23881953 PMC4021397

[B757] Yang D. Sun Y. Y. Nemkul N. Baumann J. M. Shereen A. Dunn R. S. (2013b). Plasminogen activator inhibitor-1 mitigates brain injury in a rat model of infection-sensitized neonatal hypoxia-ischemia. *Cereb. Cortex* 23 1218–1229. 10.1093/cercor/bhs115 22556277 PMC3615353

[B758] Yao L. Kan E. M. Kaur C. Dheen S. T. Hao A. Lu J. (2013). Notch-1 signaling regulates microglia activation via NF-kappaB pathway after hypoxic exposure in vivo and in vitro. *PLoS ONE* 8:e78439 10.1371/journal.pone.0078439 PMC3819391 24223152

[B759] Yazaki M. Liepnieks J. J. Murrell J. R. Takao M. Guenther B. Piccardo P. (2001). Biochemical characterization of a neuroserpin variant associated with hereditary dementia. *Am. J. Pathol.* 158 227–233. 10.1016/S0002-9440(10)63961-2 11141496 PMC1850267

[B760] Yepes M. (2015). Tissue-type plasminogen activator is a neuroprotectant in the central nervous system. *Front. Cell. Neurosci.* 9:304 10.3389/fncel.2015.00304 PMC4538299 26347605

[B761] Yepes M. Lawrence D. A. (2004a). Neuroserpin: a selective inhibitor of tissue-type plasminogen activator in the central nervous system. *Thromb. Haemost.* 91 457–464. 10.1160/th03-12-0766 14983220

[B762] Yepes M. Lawrence D. A. (2004b). New functions for an old enzyme: nonhemostatic roles for tissue-type plasminogen activator in the central nervous system. *Exp. Biol. Med.* 229 1097–1104. 10.1177/153537020422901103 15564435

[B763] Yepes M. Lawrence D. A. (2004c). Tissue-type plasminogen activator and neuroserpin: a well-balanced act in the nervous system? *Trends Cardiovasc. Med.* 14 173–180. 15261888 10.1016/j.tcm.2004.03.004

[B764] Yepes M. Sandkvist M. Coleman T. A. Moore E. Wu J. Y. Mitola D. (2002). Regulation of seizure spreading by neuroserpin and tissue-type plasminogen activator is plasminogen-independent. *J. Clin. Invest.* 109 1571–1578. 10.1172/JCI0214308 12070304 PMC151009

[B765] Yepes M. Sandkvist M. Wong M. K. Coleman T. A. Smith E. Cohan S. L. (2000). Neuroserpin reduces cerebral infarct volume and protects neurons from ischemia-induced apoptosis. *Blood* 96 569–576. 10887120

[B766] Yoon B. H. Kim C. J. Romero R. Jun J. K. Park K. H. Choi S. T. (1997). Experimentally induced intrauterine infection causes fetal brain white matter lesions in rabbits. *Am. J. Obstet. Gynecol.* 177 797–802. 10.1016/S0002-9378(97)70271-0 9369822

[B767] You Y. Kaur C. (2000). Expression of induced nitric oxide synthase in amoeboid microglia in postnatal rats following an exposure to hypoxia. *Neurosci. Lett.* 279 101–104. 10.1016/S0304-3940(99)00967-2 10674631

[B768] Yuan T. M. Yu H. M. Gu W. Z. Li J. P. (2005). White matter damage and chemokine induction in developing rat brain after intrauterine infection. *J. Perinatal Med.* 33 415–422. 10.1515/JPM.2005.074 16238536

[B769] Zanelli S. Goodkin H. P. Kowalski S. Kapur J. (2014). Impact of transient acute hypoxia on the developing mouse EEG. *Neurobiol. Dis.* 68 37–46. 10.1016/j.nbd.2014.03.005 24636798 PMC4422060

[B770] Zeinieh M. P. Talhouk R. S. El-Sabban M. E. Mikati M. A. (2010). Differential expression of hippocampal connexins after acute hypoxia in the developing brain. *Brain Dev.* 32 810–817. 10.1016/j.braindev.2009.11.003 20034754

[B771] Zeinstra E. Fock J. M. Begeer J. H. van Weerden T. W. Maurits N. M. Zweens M. J. (2001). The prognostic value of serial EEG recordings following acute neonatal asphyxia in full-term infants. *Eur. J. Paediatr. Neurol.* 5 155–160. 10.1053/ejpn.2001.0496 11587379

[B772] Zhang C. P. Zhu L. L. Zhao T. Zhao H. Huang X. Ma X. (2006). Characteristics of neural stem cells expanded in lowered oxygen and the potential role of hypoxia-inducible factor-1Alpha. *Neurosignals* 15 259–265. 10.1159/000103385 17551265

[B773] Zhang Q. Ding Y. Yao Y. Yu Y. Yang L. Cui H. (2013). Creating rat model for hypoxic brain damage in neonates by oxygen deprivation. *PLoS ONE* 8:e83589 10.1371/journal.pone.0083589 PMC3866139 24358300

[B774] Zhang Q. Yuan L. Liu D. Wang J. Wang S. Zhang Q. (2014). Hydrogen sulfide attenuates hypoxia-induced neurotoxicity through inhibiting microglial activation. *Pharmacol. Res.* 84 32–44. 10.1016/j.phrs.2014.04.009 24788079

[B775] Zhang X. Zhang Q. Li W. Nie D. Chen W. Xu C. (2014). Therapeutic effect of human umbilical cord mesenchymal stem cells on neonatal rat hypoxic–ischemic encephalopathy. *J. Neurosci. Res.* 92 35–45. 10.1002/jnr.23304 24265136

[B776] Zhang Y. He K. Wang F. Li X. Liu D. (2015). Notch-1 signaling regulates astrocytic proliferation and activation after hypoxia exposure. *Neurosci. Lett.* 603 12–18. 10.1016/j.neulet.2015.07.009 26182882

[B777] Zhang Z. Zhang L. Yepes M. Jiang Q. Li Q. Arniego P. (2002). Adjuvant treatment with neuroserpin increases the therapeutic window for tissue-type plasminogen activator administration in a rat model of embolic stroke. *Circulation* 106 740–745. 10.1161/01.CIR.0000023942.10849.41 12163437

[B778] Zhao B. Q. Tejima E. Lo E. H. (2007). Neurovascular proteases in brain injury, hemorrhage and remodeling after stroke. *Stroke* 38(Suppl. 2), 748–752. 10.1161/01.STR.0000253500.32979.d1 17261731

[B779] Zheng T. Weiss M. D. (2013). Neonatal transplant in hypoxic injury. *Methods Mol. Biol.* 1059 147–156. 10.1007/978-1-62703-574-3_13 23934841

[B780] Zheng X. R. Zhang S. S. Yin F. Tang J. L. Yang Y. J. Wang X. (2012). Neuroprotection of VEGF-expression neural stem cells in neonatal cerebral palsy rats. *Behav. Brain Res.* 230 108–115. 10.1016/j.bbr.2012.01.026 22342488

[B781] Zhou C. Lippman J. J. Sun H. Jensen F. E. (2011). Hypoxia-induced neonatal seizures diminish silent synapses and long-term potentiation in hippocampal CA1 neurons. *J. Neurosci.* 31 18211–18222. 10.1523/JNEUROSCI.4838-11.2011 22171027 PMC3282023

[B782] Zhu C. Kang W. Xu F. Cheng X. Zhang Z. Jia L. (2009). Erythropoietin improved neurologic outcomes in newborns with hypoxic-ischemic encephalopathy. *Pediatrics* 124 e218–e226. 10.1542/peds.2008-3553 19651565

[B783] Zhu C. Xu F. Wang X. Shibata M. Uchiyama Y. Blomgren K. (2006). Different apoptotic mechanisms are activated in male and female brains after neonatal hypoxia-ischaemia. *J. Neurochem.* 96 1016–1027. 10.1111/j.1471-4159.2005.03639.x 16412092

